# Supplementary material for: Factors associated with positive user experience with primary healthcare providers in Mexico: a multilevel modelling approach using national cross-sectional data
Source: BMJ Open. 2020 Jan 14;10(1):e029818. doi: 10.1136/bmjopen-2019-029818 (PMC7045193; doi:10.1136/bmjopen-2019-029818)
Supplement: Supplementary data [file bmjopen-2019-029818supp001.pdf]

Supplemental Information, **Factors associated with positive user experience with primary healthcare providers in Mexico: A multi-level modeling approach using national cross-sectional data**

Supplemental Table 1: Characteristics of Respondents for Incomplete and Complete Cases

	Incomplete (N = 1592)	Complete (N = 25745)	Total (N = 27337)
Sex of Patient			
Female	1123 (71%)	18313 (71%)	19436 (71%)
Male	469 (29%)	7432 (29%)	7901 (29%)
Age			
Bottom Quartile (18 – 33)	346 (22%)	6384 (25%)	6730 (25%)
25-50 Quartile (34 – 48)	367 (23%)	6261 (24%)	6628 (24%)
50-75 Quartile (49 – 62)	420 (26%)	6555 (25%)	6975 (26%)
Top Quartile (63 – 99)	459 (29%)	6545 (25%)	7004 (26%)
Education			
Without Schooling	272 (18%)	4309 (17%)	4581 (17%)
Completed Primary School	818 (53%)	13792 (54%)	14610 (54%)
High School or higher	460 (30%)	7644 (30%)	8104 (30%)
Type of consultation:			
Medical consultation	1393 (88%)	23113 (90%)	24506 (90%)
PREVENIMS (preventive care consultation by nurses)	171 (11%)	2023 (8%)	2194 (8%)
Dental consultation	28 (2%)	609 (2%)	637 (2%)
ICD 10 Code (Grouped)			
Endocrine, nutritional and metabolic diseases	291 (20%)	4843 (19%)	5134 (19%)
Diseases of the circulatory system	314 (22%)	5128 (20%)	5442 (20%)
Diseases of the respiratory system	131 (9%)	2352 (9%)	2483 (9%)
Diseases of the digestive system	84 (6%)	1705 (7%)	1789 (7%)
Diseases of the musculoskeletal system and connective tissue	88 (6%)	1692 (7%)	1780 (7%)
Pregnancy, childbirth and the puerperium	85 (6%)	1846 (7%)	1931 (7%)
Symptoms, signs and abnormal clinical and laboratory findings, not elsewhere classified	99 (7%)	1683 (7%)	1782 (7%)
Injury, poisoning and certain other consequences of external causes	71 (5%)	1515 (6%)	1586 (6%)
Factors influencing health status and contact with health services	104 (7%)	1831 (7%)	1935 (7%)
Other	158 (11%)	3150 (12%)	3308 (12%)

Supplemental Figure 1: Distribution of Inverse Probability Weights for Incomplete and Complete Cases (N=25,745)

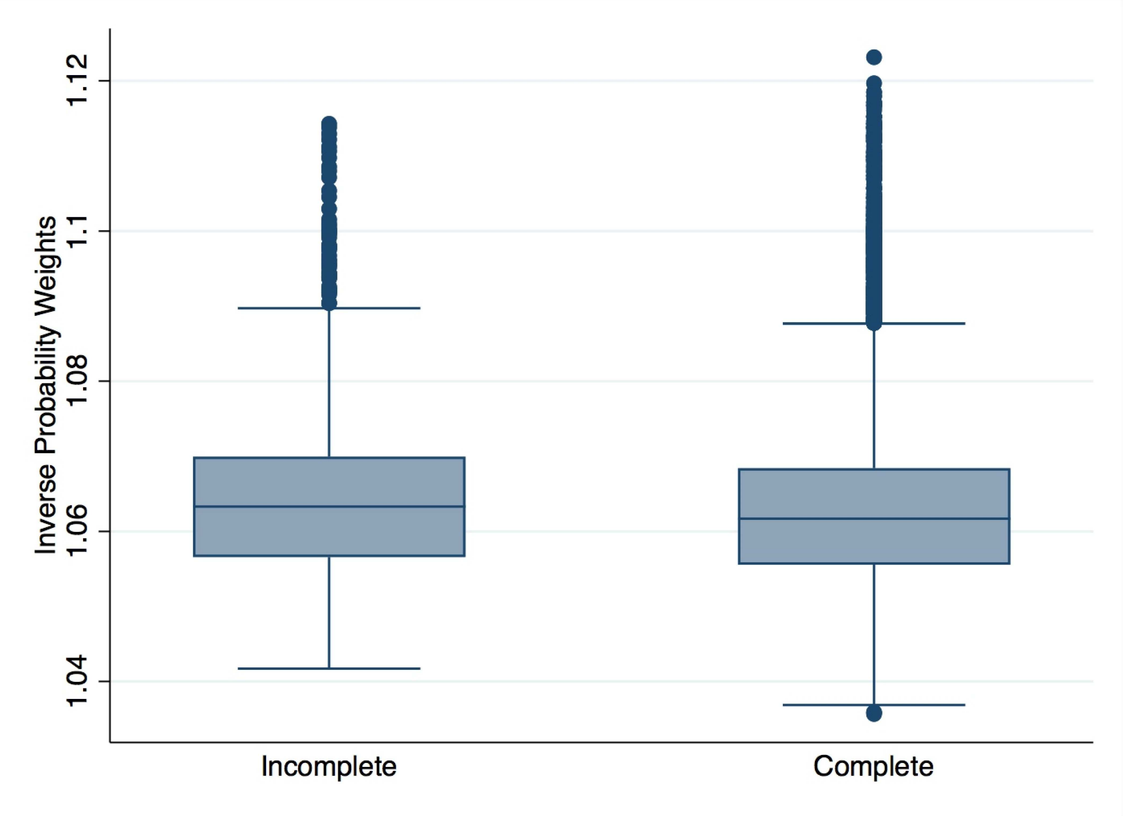

Supplemental Figure 2: Formulas

ICC:

Level 2:

$$\frac{\widehat{\tau_2^2} + \widehat{\tau_3^2}}{\widehat{\tau_2^2} + \widehat{\tau_3^2} + \frac{\pi^2}{3}}$$

Level 3:

$$\frac{\widehat{\tau_3^2}}{\widehat{\tau_2^2} + \widehat{\tau_3^2} + \frac{\pi^2}{3}}$$

IOR:

Level 2:

$$\left[\exp\left(\widehat{\alpha_2} + \sqrt{2\widehat{\tau_2^2}} * \Phi^{-1}(0.1)\right), \exp\left(\widehat{\alpha_2} + \sqrt{2\widehat{\tau_2^2}} * \Phi^{-1}(0.9)\right)\right]$$

Level 3:

$$\left[\exp\left(\widehat{\alpha_3} + \sqrt{2\widehat{\tau_3^2}} * \Phi^{-1}(0.1)\right), \exp\left(\widehat{\alpha_3} + \sqrt{2\widehat{\tau_3^2}} * \Phi^{-1}(0.9)\right)\right]$$

Population Average Adjusted Odds Ratios:

Level 2:

$$\exp(\widehat{\alpha_{PA,2}}) = \exp\left(\frac{\widehat{\alpha_2}}{\sqrt{1 + \left(16^2 * \frac{3}{(15 * \pi)^2}\right) * \widehat{\tau_2^2}}}\right)$$

Level 3:

$$\exp(\widehat{\alpha_{PA,3}}) = \exp\left(\frac{\widehat{\alpha_3}}{\sqrt{1 + \left(16^2 * \frac{3}{(15 * \pi)^2}\right) * \widehat{\tau_3^2}}}\right)$$

Where the subscript denotes the level of the variable, and level 2 variances (facility-level) are conditional on the third level (state-level), $\widehat{\tau^2}$ is the estimated group variance, and $\hat{\alpha}$ is an estimated regression coefficient. Φ represents the cumulative distribution function of the standard normal distribution.

Supplemental Table 2: Models 1-4 for all outcomes

Please note coefficients are not population-adjusted

```
---------------------------------------------------------------------------------------------
*I) Flat Model with Individual Covariates for Communication
Note: Stage 1 is sampled with replacement; further stages will be ignored for variance
      estimation.

      pweight: <none>
          VCE: linearized
  Single unit: missing
     Strata 1: <one>
         SU 1: unidmed
        FPC 1: <zero>
     Weight 1: facility_weight
     Strata 2: <one>
         SU 2: <observations>
        FPC 2: <zero>
     Weight 2: rescaled_patient_weights
(running logit on estimation sample)

Survey: Logistic regression

Number of strata   =         1                Number of obs     =     27,337
Number of PSUs     =       319                Population size   = 68,852.018
                                              Subpop. no. obs   =     25,745
                                              Subpop. size      = 64,704.336
                                              Design df         =        318
                                              F(  17,    302)   =       5.89
                                              Prob > F          =     0.0000

--------------------------------------------------------------------------------------------
                           |             Linearized
             Communication |      Coef.   Std. Err.      t    P>|t|     [95% Conf. Interval]
---------------------------+----------------------------------------------------------------
                 age_quart |
           25-50 Quartile  |   .0721386   .0519142     1.39   0.166         -.03    .1742772
           50-75 Quartile  |   .1310581   .0623401     2.10   0.036      .008407    .2537091
             Top Quartile  |   .2501662   .0746586     3.35   0.001      .103279    .3970534
                           |
                  sex_male |    .176233   .0417178     4.22   0.000     .0941551    .2583108
                           |
                 education |
 Completed Primary School  |  -.1652057    .065023    -2.54   0.012    -.2931353   -.0372761
    High School or higher  |  -.2300984   .0663483    -3.47   0.001    -.3606354   -.0995614
                           |
                 icd10_bin |
Endocrine, nutritional ..  |   .0034759   .0622057     0.06   0.955    -.1189108    .1258626
Diseases of the respira..  |  -.0665038   .0706778    -0.94   0.347     -.205559    .0725515
Diseases of the digesti..  |  -.1280427   .1026104    -1.25   0.213    -.3299238    .0738384
Diseases of the musculo..  |  -.2116943   .0894267    -2.37   0.019     -.387637   -.0357515
Pregnancy, childbirth a..  |   .2507037   .0971022     2.58   0.010     .0596597    .4417477
Symptoms, signs and abn..  |  -.1040915   .0953784    -1.09   0.276    -.2917439    .0835608
Injury, poisoning and c..  |  -.1512712   .0883329    -1.71   0.088     -.325062    .0225196
Factors influencing hea..  |   .1223723   .1084912     1.13   0.260    -.0910789    .3358235
                    Other  |   -.114026    .071712    -1.59   0.113    -.2551159     .027064
                           |
                   service |
PREVENIMS (preventive ..)  |   .0203624   .0829673     0.25   0.806    -.1428716    .1835965
```

```
     Dental consultation  |   .2658778   .1404628     1.89   0.059     -.010476    .5422315
                          |
                    _cons |   1.204737   .1144617    10.53   0.000     .9795395    1.429935
------------------------------------------------------------------------------------------
*II) Add RE for Facility for Communication
(running melogit on estimation sample)

Survey: Mixed-effects logistic regression

Number of strata   =         1                 Number of obs     =     27,337
Number of PSUs     =       319                 Population size   = 68,852.018
                                               Subpop. no. obs   =     25,745
                                               Subpop. size      = 64,704.336
                                               Design df         =        318
                                               F(  17,    302)   =       5.52
                                               Prob > F          =     0.0000

------------------------------------------------------------------------------------------
                          |             Linearized
            Communication |      Coef.   Std. Err.      t    P>|t|     [95% Conf. Interval]
--------------------------+---------------------------------------------------------------
                          |
                age_quart |
          25-50 Quartile  |    .074767   .0537437     1.39   0.165    -.0309712    .1805052
          50-75 Quartile  |     .13805   .0603951     2.29   0.023     .0192256    .2568743
            Top Quartile  |   .2533195   .0717119     3.53   0.000     .1122298    .3944093
                          |
                 sex_male |   .1643417   .0422591     3.89   0.000      .081199    .2474844
                          |
                education |
 Completed Primary School |  -.1223788   .0634736    -1.93   0.055      -.24726    .0025024
    High School or higher |  -.1935813   .0634676    -3.05   0.002    -.3184508   -.0687119
                          |
                icd10_bin |
Endocrine, nutritional .. |   .0138917   .0639729     0.22   0.828    -.1119718    .1397552
Diseases of the respira.. |  -.0694817   .0749529    -0.93   0.355    -.2169481    .0779846
Diseases of the digesti.. |  -.1320547   .1037108    -1.27   0.204    -.3361007    .0719913
Diseases of the musculo.. |  -.2054198   .0910969    -2.25   0.025    -.3846486   -.0261909
Pregnancy, childbirth a.. |   .2578102   .0993497     2.59   0.010     .0623445     .453276
Symptoms, signs and abn.. |  -.1182738   .0980881    -1.21   0.229    -.3112573    .0747098
Injury, poisoning and c.. |  -.1355994    .091086    -1.49   0.138    -.3148066    .0436079
Factors influencing hea.. |   .0986902    .105488     0.94   0.350    -.1088524    .3062328
                    Other |  -.1227894   .0723211    -1.70   0.091    -.2650776    .0194988
                          |
                  service |
PREVENIMS (preventive ..) |   .0239104   .0816092     0.29   0.770    -.1366518    .1844726
     Dental consultation  |   .3227617   .1441316     2.24   0.026     .0391897    .6063337
                          |
                    _cons |   1.277797   .1201587    10.63   0.000      1.04139    1.514203
--------------------------+---------------------------------------------------------------
unidmed                   |
                var(_cons)|   .2059121   .0336714                      .1492653    .2840565
------------------------------------------------------------------------------------------
*III) Facility-level Effects for Communication
(running melogit on estimation sample)

Survey: Mixed-effects logistic regression

Number of strata   =         1                 Number of obs     =     27,337
Number of PSUs     =       319                 Population size   = 68,852.018
                                               Subpop. no. obs   =     25,745
```

```
                                                Subpop. size      = 64,704.336
                                                Design df         =        318
                                                F(  22,    297)   =       6.62
                                                Prob > F          =     0.0000

------------------------------------------------------------------------------------------
                           |             Linearized
             Communication |      Coef.   Std. Err.      t    P>|t|     [95% Conf. Interval]
---------------------------+--------------------------------------------------------------
                           |
                 age_quart |
             25-50 Quartile  |   .0745965   .0536839     1.39   0.166    -.0310239    .1802169
             50-75 Quartile  |   .1418883    .060469     2.35   0.020     .0229184    .2608581
               Top Quartile  |    .258148   .0721074     3.58   0.000     .1162801    .4000158
                           |
                  sex_male |   .1617564   .0423419     3.82   0.000     .0784507     .245062
                           |
                 education |
   Completed Primary School  |  -.1165275   .0630907    -1.85   0.066    -.2406555    .0076005
      High School or higher  |  -.1842632   .0633158    -2.91   0.004    -.3088341   -.0596924
                           |
                 icd10_bin |
 Endocrine, nutritional ..  |   .0145716   .0636643     0.23   0.819    -.1106849    .1398282
 Diseases of the respira..  |  -.0718691   .0747377    -0.96   0.337     -.218912    .0751738
 Diseases of the digesti..  |  -.1301095   .1034579    -1.26   0.209     -.333658     .073439
 Diseases of the musculo..  |  -.2021127   .0909805    -2.22   0.027    -.3811125   -.0231129
 Pregnancy, childbirth a..  |    .261052   .0993929     2.63   0.009     .0655012    .4566028
 Symptoms, signs and abn..  |  -.1214599   .0977301    -1.24   0.215    -.3137392    .0708193
 Injury, poisoning and c..  |  -.1308762   .0909873    -1.44   0.151    -.3098893     .048137
 Factors influencing hea..  |   .0939097   .1055378     0.89   0.374    -.1137307    .3015502
                     Other  |  -.1229068   .0723339    -1.70   0.090    -.2652202    .0194066
                           |
                   service |
 PREVENIMS (preventive ..)  |   .0204962    .081476     0.25   0.802    -.1398039    .1807964
       Dental consultation  |   .3235917   .1438569     2.25   0.025     .0405601    .6066232
                           |
                 fac_pop01 |   -1.12813   .2994017    -3.77   0.000    -1.717189    -.539072
         fac_consultrms01 |   .1366501   .2653941     0.51   0.607    -.3855001    .6588003
              fac_diabetes |   .1797619   .0824758     2.18   0.030     .0174947    .3420291
               fac_nurserm |  -.1329903   .1386105    -0.96   0.338    -.4056998    .1397192
             fac_nutrition |   -.064361   .0632269    -1.02   0.309    -.1887569     .060035
                     _cons |   1.398286     .13636    10.25   0.000     1.130005    1.666568
---------------------------+--------------------------------------------------------------
unidmed                    |
                 var(_cons)|    .181121   .0300568                          .1306692    .2510523
------------------------------------------------------------------------------------------
*IV) Add RE for States for Communication
Note: Stage 1 is sampled with replacement; further stages will be ignored for variance
      estimation.

      pweight: <none>
          VCE: linearized
  Single unit: missing
     Strata 1: <one>
         SU 1: entidad
        FPC 1: <zero>
     Weight 1: state_weight
     Strata 2: <one>
         SU 2: unidmed
        FPC 2: <zero>
     Weight 2: facility_weight
```

```
     Strata 3: <one>
         SU 3: <observations>
        FPC 3: <zero>
     Weight 3: rescaled_patient_weights
(running melogit on estimation sample)

Survey: Mixed-effects logistic regression

Number of strata   =         1                Number of obs     =     27,337
Number of PSUs     =        32                Population size   = 68,852.018
                                              Subpop. no. obs   =     25,745
                                              Subpop. size      = 64,704.336
                                              Design df         =         31
                                              F(  22,     10)   =      13.90
                                              Prob > F          =     0.0001

--------------------------------------------------------------------------------------------
                           |             Linearized
             Communication |      Coef.   Std. Err.      t    P>|t|     [95% Conf. Interval]
---------------------------+----------------------------------------------------------------
                           |
                 age_quart |
           25-50 Quartile  |   .0738536   .0644009     1.15   0.260    -.0574928       .2052
           50-75 Quartile  |   .1445787   .0650096     2.22   0.034     .0119908    .2771666
             Top Quartile  |   .2612484   .0713457     3.66   0.001     .1157378     .406759
                           |
                  sex_male |   .1662661   .0300724     5.53   0.000     .1049331    .2275991
                           |
                 education |
 Completed Primary School  |  -.1035327   .0603783    -1.71   0.096    -.2266751    .0196097
    High School or higher  |  -.1703817   .0528894    -3.22   0.003    -.2782504   -.0625131
                           |
                 icd10_bin |
Endocrine, nutritional ..  |   .0206051    .072258     0.29   0.777    -.1267661    .1679763
Diseases of the respira..  |  -.0619315   .0669224    -0.93   0.362    -.1984205    .0745576
Diseases of the digesti..  |  -.1292431   .0904469    -1.43   0.163    -.3137108    .0552246
Diseases of the musculo..  |  -.1966096    .107644    -1.83   0.077     -.416151    .0229318
Pregnancy, childbirth a..  |   .2657592   .1218505     2.18   0.037     .0172436    .5142748
Symptoms, signs and abn..  |  -.1181645   .1080201    -1.09   0.282    -.3384729    .1021439
Injury, poisoning and c..  |  -.1324661   .0924996    -1.43   0.162    -.3211202    .0561881
Factors influencing hea..  |   .1104231   .1053465     1.05   0.303    -.1044326    .3252788
                    Other  |  -.1211925   .0796557    -1.52   0.138    -.2836515    .0412665
                           |
                   service |
PREVENIMS (preventive ..)  |   .0194082   .0895295     0.22   0.830    -.1631884    .2020047
     Dental consultation   |   .3171764   .1299256     2.44   0.021     .0521915    .5821614
                           |
                 fac_pop01 |  -1.173962   .4244646    -2.77   0.009    -2.039664   -.3082611
          fac_consultrms01 |   .5061666   .3509444     1.44   0.159    -.2095892    1.221922
              fac_diabetes |   .0251968   .0756337     0.33   0.741    -.1290591    .1794527
               fac_nurserm |  -.1401575   .1134644    -1.24   0.226    -.3715697    .0912548
             fac_nutrition |   -.000692   .0854003    -0.01   0.994     -.174867     .173483
                     _cons |   1.360566   .1373071     9.91   0.000     1.080526    1.640605
---------------------------+----------------------------------------------------------------
entidad                    |
                 var(_cons)|   .1037982   .0371667                      .0500072    .2154505
---------------------------+----------------------------------------------------------------
entidad>unidmed            |
                 var(_cons)|   .1064024   .0311615                      .0585527    .1933555
--------------------------------------------------------------------------------------------
*V) Add state poverty level for Communication
```

```
(running melogit on estimation sample)

Survey: Mixed-effects logistic regression

Number of strata   =         1                Number of obs     =     27,337
Number of PSUs     =        32                Population size   = 68,852.018
                                              Subpop. no. obs   =     25,745
                                              Subpop. size      = 64,704.336
                                              Design df         =         31
                                              F(  23,      9)   =      12.02
                                              Prob > F          =     0.0003

---------------------------------------------------------------------------------------------
                            |             Linearized
              Communication |      Coef.   Std. Err.      t    P>|t|     [95% Conf. Interval]
----------------------------+----------------------------------------------------------------
                            |
                  age_quart |
            25-50 Quartile  |   .0738168   .0643873     1.15   0.260    -.0575019    .2051356
            50-75 Quartile  |    .144545   .0649773     2.22   0.034     .0120228    .2770672
              Top Quartile  |   .2612314   .0713038     3.66   0.001     .1158063    .4066565
                            |
                   sex_male |   .1661988   .0300511     5.53   0.000     .1049092    .2274884
                            |
                  education |
 Completed Primary School   |  -.1034459   .0603547    -1.71   0.097    -.2265401    .0196483
    High School or higher   |  -.1704332   .0528333    -3.23   0.003    -.2781874   -.0626791
                            |
                  icd10_bin |
Endocrine, nutritional ..   |   .0205323   .0722378     0.28   0.778    -.1267977    .1678623
Diseases of the respira..   |  -.0619767   .0669156    -0.93   0.362     -.198452    .0744987
Diseases of the digesti..   |  -.1293143   .0904143    -1.43   0.163    -.3137155     .055087
Diseases of the musculo..   |   -.196657   .1076437    -1.83   0.077    -.4161979    .0228839
Pregnancy, childbirth a..   |   .2656754    .121806     2.18   0.037     .0172505    .5141003
Symptoms, signs and abn..   |  -.1183117   .1078812    -1.10   0.281    -.3383369    .1017135
Injury, poisoning and c..   |  -.1324501   .0925091    -1.43   0.162    -.3211235    .0562234
Factors influencing hea..   |   .1102726   .1052054     1.05   0.303    -.1042952    .3248403
                    Other   |  -.1212962   .0796063    -1.52   0.138    -.2836544    .0410619
                            |
                    service |
PREVENIMS (preventive ..)   |   .0193896   .0895162     0.22   0.830    -.1631799    .2019592
      Dental consultation   |   .3171689   .1299158     2.44   0.021      .052204    .5821339
                            |
                  fac_pop01 |  -1.174488   .4249714    -2.76   0.010    -2.041223   -.3077532
          fac_consultrms01 |   .5082048   .3527097     1.44   0.160    -.2111514    1.227561
               fac_diabetes |   .0250445   .0757642     0.33   0.743    -.1294775    .1795666
                fac_nurserm |  -.1392225     .11319    -1.23   0.228     -.370075      .09163
              fac_nutrition |  -.0012475   .0857079    -0.01   0.988    -.1760498    .1735549
            state_poverty01 |   .1140767   .2403637     0.47   0.638    -.3761483    .6043017
                      _cons |   1.310954   .2084665     6.29   0.000     .8857833    1.736124
----------------------------+----------------------------------------------------------------
entidad                     |
                  var(_cons)|   .1028885   .0372656                      .0491533    .2153676
----------------------------+----------------------------------------------------------------
entidad>unidmed             |
                  var(_cons)|   .1064125   .0311536                      .0585704    .1933336
---------------------------------------------------------------------------------------------
*I) Flat Model with Individual Covariates for Share_concerns
Note: Stage 1 is sampled with replacement; further stages will be ignored for variance
      estimation.
```

```
      pweight: <none>
          VCE: linearized
  Single unit: missing
     Strata 1: <one>
         SU 1: unidmed
        FPC 1: <zero>
     Weight 1: facility_weight
     Strata 2: <one>
         SU 2: <observations>
        FPC 2: <zero>
     Weight 2: rescaled_patient_weights
(running logit on estimation sample)

Survey: Logistic regression

Number of strata   =         1                Number of obs     =     27,337
Number of PSUs     =       319                Population size   = 68,852.018
                                              Subpop. no. obs   =     25,745
                                              Subpop. size      = 64,704.336
                                              Design df         =        318
                                              F(  17,    302)   =      34.31
                                              Prob > F          =     0.0000

-------------------------------------------------------------------------------------------
                           |             Linearized
            Share_concerns |      Coef.   Std. Err.      t    P>|t|     [95% Conf. Interval]
---------------------------+---------------------------------------------------------------
                 age_quart |
           25-50 Quartile  |  -.1963475   .0819595    -2.40   0.017    -.3575989   -.0350961
           50-75 Quartile  |   -.216171   .0921544    -2.35   0.020    -.3974804   -.0348616
             Top Quartile  |  -.2414114   .1092795    -2.21   0.028    -.4564137   -.0264092
                           |
                  sex_male |   .0664078   .0667259     1.00   0.320    -.0648722    .1976878
                           |
                 education |
 Completed Primary School  |  -.0382979   .0763811    -0.50   0.616     -.188574    .1119781
    High School or higher  |   .0470606   .0890162     0.53   0.597    -.1280745    .2221956
                           |
                 icd10_bin |
Endocrine, nutritional ..  |  -.0009121   .1026431    -0.01   0.993    -.2028574    .2010332
Diseases of the respira..  |   .1319851   .1402913     0.94   0.348    -.1440313    .4080016
Diseases of the digesti..  |  -.1700052   .1398109    -1.22   0.225    -.4450764    .1050659
Diseases of the musculo..  |  -.2259857   .1274163    -1.77   0.077    -.4766712    .0246999
Pregnancy, childbirth a..  |  -.0919085   .1409401    -0.65   0.515    -.3692014    .1853844
Symptoms, signs and abn..  |  -.1118509   .1397744    -0.80   0.424    -.3868504    .1631486
Injury, poisoning and c..  |  -.4820752   .1186258    -4.06   0.000    -.7154658   -.2486845
Factors influencing hea..  |  -.9587249   .1114824    -8.60   0.000    -1.178061   -.7393887
                    Other  |  -.1475031   .1128718    -1.31   0.192     -.369573    .0745667
                           |
                   service |
PREVENIMS (preventive ..)  |   -1.27743   .0846704   -15.09   0.000    -1.444015   -1.110845
      Dental consultation  |   .0949897   .1941323     0.49   0.625    -.2869563    .4769358
                           |
                     _cons |     2.8449   .1702974    16.71   0.000     2.509848    3.179951
-------------------------------------------------------------------------------------------
*II) Add RE for Facility for Share_concerns
(running melogit on estimation sample)

Survey: Mixed-effects logistic regression

Number of strata   =         1                Number of obs     =     27,337
```

```
Number of PSUs     =       319                 Population size   = 68,852.018
                                               Subpop. no. obs   =     25,745
                                               Subpop. size      = 64,704.336
                                               Design df         =        318
                                               F(  17,    302)   =      33.45
                                               Prob > F          =     0.0000

---------------------------------------------------------------------------------------------
                           |             Linearized
            Share_concerns |      Coef.   Std. Err.      t    P>|t|     [95% Conf. Interval]
---------------------------+-----------------------------------------------------------------
                           |
                 age_quart |
           25-50 Quartile  |  -.1977085   .0832451    -2.38   0.018    -.3614893   -.0339277
           50-75 Quartile  |  -.2030779   .0908244    -2.24   0.026    -.3817707   -.0243852
             Top Quartile  |  -.2280504   .1091908    -2.09   0.038    -.4428781   -.0132227
                           |
                  sex_male |   .0632317   .0665015     0.95   0.342    -.0676069    .1940702
                           |
                 education |
 Completed Primary School  |  -.0133517    .077376    -0.17   0.863    -.1655852    .1388818
    High School or higher  |   .0839919    .089364     0.94   0.348    -.0918275    .2598112
                           |
                 icd10_bin |
Endocrine, nutritional ..  |  -.0003744   .1034186    -0.00   0.997    -.2038456    .2030968
Diseases of the respira..  |   .1421993   .1407525     1.01   0.313    -.1347245    .4191231
Diseases of the digesti..  |  -.1560012   .1416805    -1.10   0.272    -.4347508    .1227484
Diseases of the musculo..  |  -.2136876   .1277391    -1.67   0.095    -.4650081     .037633
Pregnancy, childbirth a..  |  -.0831523   .1408744    -0.59   0.555    -.3603158    .1940113
Symptoms, signs and abn..  |  -.1157391   .1400367    -0.83   0.409    -.3912546    .1597765
Injury, poisoning and c..  |  -.4752238   .1189693    -3.99   0.000    -.7092901   -.2411575
Factors influencing hea..  |  -.9576453   .1108565    -8.64   0.000     -1.17575   -.7395405
                    Other  |  -.1429192   .1136525    -1.26   0.209     -.366525    .0806866
                           |
                   service |
PREVENIMS (preventive ..)  |   -1.30918   .0861955   -15.19   0.000    -1.478766   -1.139595
      Dental consultation  |   .0919705   .1951279     0.47   0.638    -.2919343    .4758752
                           |
                     _cons |   2.919982   .1736988    16.81   0.000     2.578238    3.261726
---------------------------+-----------------------------------------------------------------
unidmed                    |
                var(_cons)|   .1902232   .0341005                       .133687    .2706686
---------------------------------------------------------------------------------------------
*III) Facility-level Effects for Share_concerns
(running melogit on estimation sample)

Survey: Mixed-effects logistic regression

Number of strata   =         1                 Number of obs     =     27,337
Number of PSUs     =       319                 Population size   = 68,852.018
                                               Subpop. no. obs   =     25,745
                                               Subpop. size      = 64,704.336
                                               Design df         =        318
                                               F(  22,    297)   =      25.49
                                               Prob > F          =     0.0000

---------------------------------------------------------------------------------------------
                           |             Linearized
            Share_concerns |      Coef.   Std. Err.      t    P>|t|     [95% Conf. Interval]
---------------------------+-----------------------------------------------------------------
                           |
```

```
                 age_quart |
             25-50 Quartile  |  -.1998499   .0837436    -2.39   0.018    -.3646113   -.0350884
             50-75 Quartile  |  -.1962364   .0910245    -2.16   0.032    -.3753228   -.0171501
               Top Quartile  |  -.2198729   .1105111    -1.99   0.047    -.4372981   -.0024477
                            |
                  sex_male |   .0557743   .0668492     0.83   0.405    -.0757483     .187297
                            |
                 education |
   Completed Primary School  |  -.0020449   .0774564    -0.03   0.979    -.1544367    .1503469
      High School or higher  |   .1026721   .0897995     1.14   0.254    -.0740041    .2793483
                            |
                 icd10_bin |
  Endocrine, nutritional ..  |  -.0002771     .10367    -0.00   0.998    -.2042429    .2036887
  Diseases of the respira..  |   .1375723   .1420331     0.97   0.333     -.141871    .4170156
  Diseases of the digesti..  |  -.1547567   .1421003    -1.09   0.277    -.4343323    .1248189
  Diseases of the musculo..  |  -.2124765   .1278028    -1.66   0.097    -.4639224    .0389694
  Pregnancy, childbirth a..  |  -.0819705   .1415853    -0.58   0.563    -.3605328    .1965918
  Symptoms, signs and abn..  |  -.1239627   .1402149    -0.88   0.377    -.3998288    .1519033
  Injury, poisoning and c..  |  -.4691149   .1192677    -3.93   0.000    -.7037683   -.2344615
  Factors influencing hea..  |  -.9743061   .1124096    -8.67   0.000    -1.195467   -.7531456
                      Other  |  -.1458597   .1144054    -1.27   0.203    -.3709467    .0792273
                            |
                   service |
  PREVENIMS (preventive ..)  |  -1.313699   .0862879   -15.22   0.000    -1.483467   -1.143932
        Dental consultation  |   .0965886    .195796     0.49   0.622    -.2886306    .4818079
                            |
                 fac_pop01 |  -.9535991   .3991065    -2.39   0.017    -1.738822   -.1683763
          fac_consultrms01 |  -.1106742   .3467813    -0.32   0.750    -.7929497    .5716013
              fac_diabetes |   .1862659   .0946715     1.97   0.050     4.16e-06    .3725275
               fac_nurserm |    .064487   .1392731     0.46   0.644    -.2095262    .3385002
             fac_nutrition |    -.01714     .07498    -0.23   0.819    -.1646596    .1303795
                     _cons |   3.054871    .183727    16.63   0.000     2.693397    3.416345
---------------------------+----------------------------------------------------------------
unidmed                    |
                 var(_cons)|   .1563907   .0314717                      .1052597    .2323589
--------------------------------------------------------------------------------------------
*IV) Add RE for States for Share_concerns
Note: Stage 1 is sampled with replacement; further stages will be ignored for variance
      estimation.

      pweight: <none>
          VCE: linearized
  Single unit: missing
     Strata 1: <one>
         SU 1: entidad
        FPC 1: <zero>
     Weight 1: state_weight
     Strata 2: <one>
         SU 2: unidmed
        FPC 2: <zero>
     Weight 2: facility_weight
     Strata 3: <one>
         SU 3: <observations>
        FPC 3: <zero>
     Weight 3: rescaled_patient_weights
(running melogit on estimation sample)

Survey: Mixed-effects logistic regression

Number of strata   =         1                  Number of obs     =     27,337
Number of PSUs     =        32                  Population size   = 68,852.018
```

```
                                                Subpop. no. obs   =     25,745
                                                Subpop. size      = 64,704.336
                                                Design df         =         31
                                                F(  22,     10)   =      25.24
                                                Prob > F          =     0.0000

------------------------------------------------------------------------------------
                           |             Linearized
            Share_concerns |      Coef.   Std. Err.      t    P>|t|     [95% Conf. Interval]
---------------------------+--------------------------------------------------------
                           |
                 age_quart |
           25-50 Quartile  |  -.1891676   .0672534    -2.81   0.008    -.3263319   -.0520033
           50-75 Quartile  |  -.1827254     .07314    -2.50   0.018    -.3318954   -.0335555
             Top Quartile  |  -.2018592   .0792979    -2.55   0.016    -.3635883   -.0401301
                           |
                  sex_male |   .0613961   .0618973     0.99   0.329    -.0648443    .1876365
                           |
                 education |
 Completed Primary School  |   .0091232   .1052271     0.09   0.931    -.2054889    .2237353
    High School or higher  |   .1288565   .1066328     1.21   0.236    -.0886226    .3463356
                           |
                 icd10_bin |
Endocrine, nutritional ..  |   .0101802   .1002269     0.10   0.920    -.1942339    .2145944
Diseases of the respira..  |   .1504957   .1590078     0.95   0.351    -.1738028    .4747942
Diseases of the digesti..  |  -.1543164   .1409729    -1.09   0.282    -.4418325    .1331998
Diseases of the musculo..  |  -.1992548    .111818    -1.78   0.085    -.4273091    .0287995
Pregnancy, childbirth a..  |  -.0697851   .1219999    -0.57   0.571    -.3186055    .1790352
Symptoms, signs and abn..  |  -.1263413   .1371278    -0.92   0.364    -.4060153    .1533326
Injury, poisoning and c..  |   -.475034   .1025005    -4.63   0.000    -.6840852   -.2659828
Factors influencing hea..  |  -.9570296   .1220179    -7.84   0.000    -1.205887   -.7081725
                    Other  |  -.1401664   .0923261    -1.52   0.139    -.3284668     .048134
                           |
                   service |
PREVENIMS (preventive ..)  |  -1.324286   .0595103   -22.25   0.000    -1.445658   -1.202914
      Dental consultation  |   .0855222    .199395     0.43   0.671    -.3211465    .4921908
                           |
                 fac_pop01 |  -.8513804   .3615294    -2.35   0.025    -1.588725   -.1140363
         fac_consultrms01  |  -.0722481   .4565006    -0.16   0.875    -1.003287    .8587909
              fac_diabetes |   .0805323   .1435956     0.56   0.579     -.212333    .3733975
               fac_nurserm |  -.0310571    .180736    -0.17   0.865    -.3996707    .3375565
             fac_nutrition |   .0720496   .1298929     0.55   0.583    -.1928687    .3369679
                     _cons |   3.028693   .2335348    12.97   0.000     2.552395     3.50499
---------------------------+--------------------------------------------------------
entidad                    |
                 var(_cons)|   .1183331   .0373483                       .062165    .2252507
---------------------------+--------------------------------------------------------
entidad>unidmed            |
                 var(_cons)|   .1041221   .0320072                       .055624    .1949051
------------------------------------------------------------------------------------
*V) Add state poverty level for Share_concerns
(running melogit on estimation sample)

Survey: Mixed-effects logistic regression

Number of strata   =         1                  Number of obs     =     27,337
Number of PSUs     =        32                  Population size   = 68,852.018
                                                Subpop. no. obs   =     25,745
                                                Subpop. size      = 64,704.336
                                                Design df         =         31
                                                F(  23,      9)   =      21.88
```

```
                                                Prob > F          =     0.0000

------------------------------------------------------------------------------------------
                          |             Linearized
           Share_concerns |      Coef.   Std. Err.      t    P>|t|     [95% Conf. Interval]
--------------------------+---------------------------------------------------------------
                          |
                age_quart |
           25-50 Quartile  |   -.188914   .0671811    -2.81   0.008    -.3259307   -.0518973
           50-75 Quartile  |  -.1824825   .0729888    -2.50   0.018    -.3313442   -.0336209
             Top Quartile  |  -.2016701   .0791763    -2.55   0.016    -.3631513    -.040189
                          |
                 sex_male |   .0615178   .0618745     0.99   0.328     -.064676    .1877116
                          |
                education |
  Completed Primary School  |   .0087758     .10525     0.08   0.934     -.205883    .2234346
     High School or higher  |   .1289782   .1066099     1.21   0.235     -.088454    .3464105
                          |
                icd10_bin |
Endocrine, nutritional ..  |   .0102353   .1002019     0.10   0.919    -.1941279    .2145984
Diseases of the respira..  |   .1504319   .1590145     0.95   0.351    -.1738804    .4747442
Diseases of the digesti..  |  -.1541799   .1410199    -1.09   0.283    -.4417918    .1334321
Diseases of the musculo..  |  -.1992044   .1118135    -1.78   0.085    -.4272496    .0288409
Pregnancy, childbirth a..  |  -.0696704   .1218989    -0.57   0.572    -.3182849    .1789441
Symptoms, signs and abn..  |  -.1260352   .1370289    -0.92   0.365    -.4055076    .1534372
Injury, poisoning and c..  |  -.4749992   .1025226    -4.63   0.000    -.6840954   -.2659029
Factors influencing hea..  |  -.9567239   .1218248    -7.85   0.000    -1.205187   -.7082605
                    Other  |  -.1399324    .092311    -1.52   0.140     -.328202    .0483371
                          |
                  service |
PREVENIMS (preventive ..)  |  -1.324235   .0594536   -22.27   0.000    -1.445492   -1.202979
      Dental consultation  |    .085601   .1994053     0.43   0.671    -.3210887    .4922907
                          |
                fac_pop01 |  -.8508376   .3611301    -2.36   0.025    -1.587367   -.1143078
         fac_consultrms01 |  -.0771689   .4577233    -0.17   0.867    -1.010702    .8563639
             fac_diabetes |   .0812226   .1437852     0.56   0.576    -.2120294    .3744745
              fac_nurserm |  -.0326914   .1811533    -0.18   0.858    -.4021559    .3367731
            fac_nutrition |   .0727493   .1299266     0.56   0.580    -.1922378    .3377364
          state_poverty01 |  -.2052001   .2465455    -0.83   0.412    -.7080329    .2976327
                    _cons |   3.117878   .2897188    10.76   0.000     2.526993    3.708764
--------------------------+---------------------------------------------------------------
entidad                   |
                var(_cons)|   .1154058   .0373783                      .0596138    .2234133
--------------------------+---------------------------------------------------------------
entidad>unidmed           |
                var(_cons)|   .1042867   .0320328                      .0557393    .1951178
------------------------------------------------------------------------------------------
*I) Flat Model with Individual Covariates for Resolve_doubts
Note: Stage 1 is sampled with replacement; further stages will be ignored for variance
      estimation.

      pweight: <none>
          VCE: linearized
  Single unit: missing
     Strata 1: <one>
         SU 1: unidmed
        FPC 1: <zero>
     Weight 1: facility_weight
     Strata 2: <one>
         SU 2: <observations>
        FPC 2: <zero>
```

```
     Weight 2: rescaled_patient_weights
(running logit on estimation sample)

Survey: Logistic regression

Number of strata   =         1                 Number of obs     =     27,337
Number of PSUs     =       319                 Population size   = 68,852.018
                                               Subpop. no. obs   =     25,745
                                               Subpop. size      = 64,704.336
                                               Design df         =        318
                                               F(  17,    302)   =       5.76
                                               Prob > F          =     0.0000

--------------------------------------------------------------------------------------------
                           |             Linearized
            Resolve_doubts |      Coef.   Std. Err.      t    P>|t|     [95% Conf. Interval]
---------------------------+----------------------------------------------------------------
                 age_quart |
           25-50 Quartile  |   -.093559   .0632403    -1.48   0.140    -.2179813    .0308633
           50-75 Quartile  |   -.023793   .0725288    -0.33   0.743      -.16649     .118904
             Top Quartile  |   .0377486   .0809958     0.47   0.641    -.1216067    .1971039
                           |
                  sex_male |  -.0073745   .0510991    -0.14   0.885    -.1079096    .0931605
                           |
                 education |
  Completed Primary School |   .0346189    .065023     0.53   0.595    -.0933106    .1625485
     High School or higher |   .1394226   .0782083     1.78   0.076    -.0144485    .2932937
                           |
                 icd10_bin |
Endocrine, nutritional ..  |  -.1484384   .0811892    -1.83   0.068    -.3081743    .0112974
Diseases of the respira..  |  -.0113877   .0885284    -0.13   0.898    -.1855631    .1627876
Diseases of the digesti..  |  -.2908084   .1049612    -2.77   0.006    -.4973146   -.0843022
Diseases of the musculo..  |  -.3015726   .1097798    -2.75   0.006     -.517559   -.0855861
Pregnancy, childbirth a..  |  -.1224683   .1018048    -1.20   0.230    -.3227643    .0778277
Symptoms, signs and abn..  |  -.5400882    .093885    -5.75   0.000    -.7248023   -.3553741
Injury, poisoning and c..  |  -.4325446   .1067835    -4.05   0.000    -.6426359   -.2224532
Factors influencing hea..  |   .1288747   .1372838     0.94   0.349    -.1412246    .3989739
                    Other  |  -.3547119   .0771014    -4.60   0.000    -.5064051   -.2030186
                           |
                   service |
PREVENIMS (preventive ..)  |   .1319719   .1027883     1.28   0.200    -.0702592    .3342029
      Dental consultation  |    .141035   .1606949     0.88   0.381    -.1751245    .4571945
                           |
                     _cons |   1.987299   .1189204    16.71   0.000     1.753329    2.221269
--------------------------------------------------------------------------------------------
*II) Add RE for Facility for Resolve_doubts
(running melogit on estimation sample)

Survey: Mixed-effects logistic regression

Number of strata   =         1                 Number of obs     =     27,337
Number of PSUs     =       319                 Population size   = 68,852.018
                                               Subpop. no. obs   =     25,745
                                               Subpop. size      = 64,704.336
                                               Design df         =        318
                                               F(  17,    302)   =       5.57
                                               Prob > F          =     0.0000

--------------------------------------------------------------------------------------------
                           |             Linearized
            Resolve_doubts |      Coef.   Std. Err.      t    P>|t|     [95% Conf. Interval]
```

```
----------------------------+----------------------------------------------------------------
                            |
                  age_quart |
            25-50 Quartile  |  -.1048848   .0673424    -1.56   0.120    -.2373777    .0276081
            50-75 Quartile  |  -.0497869   .0750062    -0.66   0.507    -.1973579    .0977842
              Top Quartile  |   -.011277   .0842838    -0.13   0.894    -.1771014    .1545474
                            |
                   sex_male |  -.0166598   .0516194    -0.32   0.747    -.1182185    .0848989
                            |
                  education |
 Completed Primary School   |   .0551933   .0640305     0.86   0.389    -.0707836    .1811701
    High School or higher   |   .1606116   .0759891     2.11   0.035     .0111067    .3101165
                            |
                  icd10_bin |
Endocrine, nutritional ..   |  -.1281452   .0830646    -1.54   0.124    -.2915708    .0352803
Diseases of the respira..   |  -.0277771   .0915074    -0.30   0.762    -.2078135    .1522593
Diseases of the digesti..   |   -.279813   .1090472    -2.57   0.011    -.4943581   -.0652679
Diseases of the musculo..   |  -.2940388   .1185829    -2.48   0.014    -.5273448   -.0607327
Pregnancy, childbirth a..   |  -.1172595   .1038886    -1.13   0.260    -.3216553    .0871364
Symptoms, signs and abn..   |   -.540188   .0955076    -5.66   0.000    -.7280947   -.3522813
Injury, poisoning and c..   |  -.3608253   .1091103    -3.31   0.001    -.5754946    -.146156
Factors influencing hea..   |   .0528295   .1375516     0.38   0.701    -.2177966    .3234557
                    Other   |  -.3751948   .0803828    -4.67   0.000    -.5333442   -.2170455
                            |
                    service |
PREVENIMS (preventive ..)   |   .2282418   .1137517     2.01   0.046     .0044407    .4520428
      Dental consultation   |    .187277   .1600202     1.17   0.243    -.1275551    .5021091
                            |
                      _cons |   2.121049   .1251103    16.95   0.000       1.8749    2.367197
----------------------------+----------------------------------------------------------------
unidmed                     |
                  var(_cons)|   .2965895   .0481751                        .21546    .4082674
---------------------------------------------------------------------------------------------
*III) Facility-level Effects for Resolve_doubts
(running melogit on estimation sample)

Survey: Mixed-effects logistic regression

Number of strata   =         1                Number of obs     =     27,337
Number of PSUs     =       319                Population size   = 68,852.018
                                              Subpop. no. obs   =     25,745
                                              Subpop. size      = 64,704.336
                                              Design df         =        318
                                              F(  22,    297)   =       5.49
                                              Prob > F          =     0.0000

---------------------------------------------------------------------------------------------
                            |             Linearized
             Resolve_doubts |      Coef.   Std. Err.      t    P>|t|     [95% Conf. Interval]
----------------------------+----------------------------------------------------------------
                            |
                  age_quart |
            25-50 Quartile  |  -.1056468   .0671555    -1.57   0.117    -.2377722    .0264785
            50-75 Quartile  |  -.0469628   .0745427    -0.63   0.529    -.1936219    .0996964
              Top Quartile  |   -.007258   .0838846    -0.09   0.931    -.1722969    .1577809
                            |
                   sex_male |   -.020024   .0518374    -0.39   0.700    -.1220117    .0819637
                            |
                  education |
 Completed Primary School   |   .0615639   .0639977     0.96   0.337    -.0643486    .1874764
    High School or higher   |   .1683225   .0757234     2.22   0.027     .0193404    .3173046
```

```
                           |
                 icd10_bin |
Endocrine, nutritional ..  |  -.1291846   .0836589    -1.54   0.124    -.2937794    .0354103
Diseases of the respira..  |   -.030711   .0919195    -0.33   0.739    -.2115581    .1501361
Diseases of the digesti..  |  -.2810543   .1088042    -2.58   0.010    -.4951214   -.0669873
Diseases of the musculo..  |  -.2940371   .1177309    -2.50   0.013     -.525667   -.0624072
Pregnancy, childbirth a..  |  -.1172918   .1038053    -1.13   0.259    -.3215239    .0869402
Symptoms, signs and abn..  |  -.5449934   .0955344    -5.70   0.000    -.7329528   -.3570341
Injury, poisoning and c..  |  -.3588143   .1088083    -3.30   0.001    -.5728894   -.1447393
Factors influencing hea..  |   .0482873   .1370837     0.35   0.725    -.2214184    .3179929
                    Other  |  -.3767199   .0806631    -4.67   0.000    -.5354206   -.2180191
                           |
                   service |
PREVENIMS (preventive ..)  |   .2237131   .1138271     1.97   0.050    -.0002363    .4476626
      Dental consultation  |   .1881435   .1598683     1.18   0.240    -.1263897    .5026766
                           |
                 fac_pop01 |  -.7013858   .4079466    -1.72   0.087    -1.504001    .1012295
          fac_consultrms01 |  -.2920155   .3627236    -0.81   0.421    -1.005657    .4216258
              fac_diabetes |   .1032776   .0955959     1.08   0.281    -.0848027     .291358
               fac_nurserm |  -.1110546   .1469678    -0.76   0.450    -.4002067    .1780975
             fac_nutrition |   .0866532   .0835638     1.04   0.301    -.0777545     .251061
                     _cons |   2.245155   .1484545    15.12   0.000     1.953078    2.537232
---------------------------+----------------------------------------------------------------
unidmed                    |
                 var(_cons)|   .2775229   .0488026                      .1963553    .3922429
--------------------------------------------------------------------------------------------
*IV) Add RE for States for Resolve_doubts
Note: Stage 1 is sampled with replacement; further stages will be ignored for variance
      estimation.

      pweight: <none>
          VCE: linearized
  Single unit: missing
     Strata 1: <one>
         SU 1: entidad
        FPC 1: <zero>
     Weight 1: state_weight
     Strata 2: <one>
         SU 2: unidmed
        FPC 2: <zero>
     Weight 2: facility_weight
     Strata 3: <one>
         SU 3: <observations>
        FPC 3: <zero>
     Weight 3: rescaled_patient_weights
(running melogit on estimation sample)

Survey: Mixed-effects logistic regression

Number of strata   =         1                Number of obs     =     27,337
Number of PSUs     =        32                Population size   = 68,852.018
                                              Subpop. no. obs   =     25,745
                                              Subpop. size      = 64,704.336
                                              Design df         =         31
                                              F(  22,     10)   =      11.27
                                              Prob > F          =     0.0002

--------------------------------------------------------------------------------------------
                           |             Linearized
             Resolve_doubts |      Coef.   Std. Err.      t    P>|t|     [95% Conf. Interval]
---------------------------+----------------------------------------------------------------
```

```
                           |
                 age_quart |
              25-50 Quartile  |  -.1099239   .0645072    -1.70   0.098    -.2414872    .0216394
              50-75 Quartile  |  -.0529968   .0758886    -0.70   0.490    -.2077725     .101779
                Top Quartile  |  -.0151998   .0726482    -0.21   0.836    -.1633668    .1329671
                           |
                  sex_male |  -.0160906   .0625494    -0.26   0.799     -.143661    .1114798
                           |
                 education |
   Completed Primary School  |   .0648781   .0520728     1.25   0.222     -.041325    .1710813
      High School or higher  |   .1682938   .0598304     2.81   0.008     .0462688    .2903187
                           |
                 icd10_bin |
  Endocrine, nutritional ..  |  -.1281272   .0935731    -1.37   0.181    -.3189709    .0627165
  Diseases of the respira..  |   -.038028   .1186624    -0.32   0.751    -.2800415    .2039855
  Diseases of the digesti..  |  -.2862091   .1537024    -1.86   0.072    -.5996873     .027269
  Diseases of the musculo..  |  -.2985614   .1326061    -2.25   0.032    -.5690134   -.0281094
  Pregnancy, childbirth a..  |  -.1198699   .1062169    -1.13   0.268    -.3365006    .0967608
  Symptoms, signs and abn..  |  -.5383298   .1008745    -5.34   0.000    -.7440647   -.3325948
  Injury, poisoning and c..  |  -.3501022   .1463964    -2.39   0.023    -.6486796   -.0515248
  Factors influencing hea..  |   .0319278   .1117272     0.29   0.777    -.1959413    .2597969
                      Other  |  -.3741038   .0917102    -4.08   0.000    -.5611479   -.1870597
                           |
                   service |
  PREVENIMS (preventive ..)  |   .2284354   .0882579     2.59   0.015     .0484322    .4084386
        Dental consultation  |      .2005   .1554239     1.29   0.207     -.116489    .5174891
                           |
                 fac_pop01 |  -.7551055   .3276321    -2.30   0.028    -1.423316   -.0868956
         fac_consultrms01 |   -.167094   .3567697    -0.47   0.643    -.8947307    .5605427
              fac_diabetes |    .111667   .0980932     1.14   0.264    -.0883954    .3117294
               fac_nurserm |  -.0246641   .1530908    -0.16   0.873    -.3368948    .2875665
             fac_nutrition |  -.0504709   .1351853    -0.37   0.711    -.3261831    .2252413
                     _cons |   2.322359   .1657151    14.01   0.000     1.984381    2.660337
---------------------------+----------------------------------------------------------------
entidad                    |
                 var(_cons)|   .1220106   .0330881                      .0701764     .212131
---------------------------+----------------------------------------------------------------
entidad>unidmed            |
                 var(_cons)|   .1805176   .0319137                      .1258716    .2588877
--------------------------------------------------------------------------------------------
*V) Add state poverty level for Resolve_doubts
(running melogit on estimation sample)

Survey: Mixed-effects logistic regression

Number of strata   =         1                  Number of obs     =     27,337
Number of PSUs     =        32                  Population size   = 68,852.018
                                                Subpop. no. obs   =     25,745
                                                Subpop. size      = 64,704.336
                                                Design df         =         31
                                                F(  23,      9)   =       9.80
                                                Prob > F          =     0.0006

--------------------------------------------------------------------------------------------
                           |             Linearized
            Resolve_doubts |      Coef.   Std. Err.      t    P>|t|     [95% Conf. Interval]
---------------------------+----------------------------------------------------------------
                           |
                 age_quart |
              25-50 Quartile  |  -.1098881   .0644738    -1.70   0.098    -.2413833    .0216072
              50-75 Quartile  |  -.0529422   .0758694    -0.70   0.490    -.2076789    .1017945
```

```
             Top Quartile  |  -.0151532   .0726566    -0.21   0.836    -.1633372    .1330308
                           |
                  sex_male |  -.0160394   .0625797    -0.26   0.799    -.1436717    .1115928
                           |
                 education |
  Completed Primary School |   .0648057   .0520615     1.24   0.223    -.0413743    .1709858
     High School or higher |   .1683599   .0598359     2.81   0.008     .0463237    .2903961
                           |
                 icd10_bin |
 Endocrine, nutritional .. |  -.1280744   .0935696    -1.37   0.181    -.3189108     .062762
 Diseases of the respira.. |  -.0380201    .118674    -0.32   0.751    -.2800573    .2040172
 Diseases of the digesti.. |  -.2861059   .1536502    -1.86   0.072    -.5994775    .0272658
 Diseases of the musculo.. |   -.298553   .1326011    -2.25   0.032    -.5689948   -.0281112
 Pregnancy, childbirth a.. |  -.1198024   .1062616    -1.13   0.268    -.3365243    .0969196
 Symptoms, signs and abn.. |  -.5381943   .1008591    -5.34   0.000    -.7438977   -.3324908
 Injury, poisoning and c.. |  -.3501081    .146396    -2.39   0.023    -.6486848   -.0515315
 Factors influencing hea.. |   .0320651   .1118723     0.29   0.776       -.1961    .2602303
                     Other |  -.3740188   .0916746    -4.08   0.000    -.5609904   -.1870473
                           |
                   service |
 PREVENIMS (preventive ..) |   .2284348   .0882488     2.59   0.015     .0484502    .4084194
      Dental consultation  |   .2004468   .1553923     1.29   0.207    -.1164778    .5173714
                           |
                 fac_pop01 |  -.7545314    .327681    -2.30   0.028    -1.422841   -.0862215
         fac_consultrms01  |  -.1695328   .3576691    -0.47   0.639    -.8990036     .559938
              fac_diabetes |   .1119431   .0978927     1.14   0.262    -.0877105    .3115966
               fac_nurserm |  -.0254535    .154103    -0.17   0.870    -.3397486    .2888417
             fac_nutrition |  -.0499346   .1353615    -0.37   0.715    -.3260062    .2261369
           state_poverty01 |  -.0947671   .3001921    -0.32   0.754     -.707013    .5174787
                     _cons |   2.363503   .2054285    11.51   0.000     1.944529    2.782478
---------------------------+----------------------------------------------------------------
entidad                    |
                 var(_cons)|   .1215241    .032831                       .0700435    .2108419
---------------------------+----------------------------------------------------------------
entidad>unidmed            |
                 var(_cons)|   .1805272    .031905                       .1258931     .258871
--------------------------------------------------------------------------------------------
*I) Flat Model with Individual Covariates for Global_rating
Note: Stage 1 is sampled with replacement; further stages will be ignored for variance
      estimation.

      pweight: <none>
          VCE: linearized
  Single unit: missing
     Strata 1: <one>
         SU 1: unidmed
        FPC 1: <zero>
     Weight 1: facility_weight
     Strata 2: <one>
         SU 2: <observations>
        FPC 2: <zero>
     Weight 2: rescaled_patient_weights
(running logit on estimation sample)

Survey: Logistic regression

Number of strata   =         1                 Number of obs     =     27,337
Number of PSUs     =       319                 Population size   = 68,852.018
                                               Subpop. no. obs   =     25,745
                                               Subpop. size      = 64,704.336
                                               Design df         =        318
```

```
                                                  F(  17,    302)   =       2.89
                                                  Prob > F          =     0.0001

------------------------------------------------------------------------------------------
                           |             Linearized
             Global_rating |      Coef.   Std. Err.      t    P>|t|     [95% Conf. Interval]
---------------------------+--------------------------------------------------------------
                 age_quart |
          25-50 Quartile  |   .0489217   .0536005     0.91   0.362    -.0565347    .1543781
          50-75 Quartile  |    .217218   .0572894     3.79   0.000     .1045039    .3299322
            Top Quartile  |   .2229387   .0676761     3.29   0.001     .0897893    .3560882
                           |
                  sex_male |    .065549    .037209     1.76   0.079     -.007658    .1387559
                           |
                 education |
  Completed Primary School  |   .1505519   .0574759     2.62   0.009     .0374707     .263633
     High School or higher  |   .3580737   .0761552     4.70   0.000     .2082421    .5079053
                           |
                 icd10_bin |
 Endocrine, nutritional ..  |  -.0229643   .0592606    -0.39   0.699    -.1395567    .0936281
 Diseases of the respira..  |  -.0762341   .0823095    -0.93   0.355     -.238174    .0857059
 Diseases of the digesti..  |  -.1447848   .0953839    -1.52   0.130    -.3324481    .0428785
 Diseases of the musculo..  |   .0346319   .0772459     0.45   0.654    -.1173457    .1866095
 Pregnancy, childbirth a..  |   .0801048    .077394     1.04   0.301    -.0721642    .2323737
 Symptoms, signs and abn..  |   .0791826   .0831464     0.95   0.342    -.0844039    .2427691
 Injury, poisoning and c..  |  -.1290744   .0985339    -1.31   0.191    -.3229352    .0647863
 Factors influencing hea..  |   .1299533   .0942587     1.38   0.169     -.055496    .3154027
                     Other  |  -.0355084   .0696345    -0.51   0.610    -.1725108    .1014941
                           |
                   service |
 PREVENIMS (preventive ..)  |   .0367234   .0786779     0.47   0.641    -.1180715    .1915183
       Dental consultation  |   .2130081   .1194187     1.78   0.075    -.0219424    .4479586
                           |
                     _cons |  -1.355186   .1089577   -12.44   0.000    -1.569555   -1.140817
------------------------------------------------------------------------------------------
*II) Add RE for Facility for Global_rating
(running melogit on estimation sample)

Survey: Mixed-effects logistic regression

Number of strata   =         1                Number of obs     =     27,337
Number of PSUs     =       319                Population size   = 68,852.018
                                                  Subpop. no. obs   =     25,745
                                                  Subpop. size      = 64,704.336
                                                  Design df         =        318
                                                  F(  17,    302)   =       3.55
                                                  Prob > F          =     0.0000

------------------------------------------------------------------------------------------
                           |             Linearized
             Global_rating |      Coef.   Std. Err.      t    P>|t|     [95% Conf. Interval]
---------------------------+--------------------------------------------------------------
                           |
                 age_quart |
          25-50 Quartile  |   .0347388   .0569718     0.61   0.542    -.0773505    .1468281
          50-75 Quartile  |   .2072743   .0556189     3.73   0.000     .0978469    .3167018
            Top Quartile  |   .1954014   .0614554     3.18   0.002     .0744908    .3163119
                           |
                  sex_male |   .0672075    .038077     1.77   0.079    -.0077073    .1421222
                           |
                 education |
```

```
 Completed Primary School  |   .1351317   .0523625     2.58   0.010     .0321109    .2381525
    High School or higher  |   .3097884   .0643747     4.81   0.000     .1831343    .4364425
                           |
                 icd10_bin |
Endocrine, nutritional ..  |  -.0404282   .0574374    -0.70   0.482    -.1534335    .0725772
Diseases of the respira..  |  -.0537951   .0838579    -0.64   0.522    -.2187814    .1111912
Diseases of the digesti..  |  -.1474168   .1003761    -1.47   0.143     -.344902    .0500683
Diseases of the musculo..  |   .0180007   .0781801     0.23   0.818    -.1358149    .1718162
Pregnancy, childbirth a..  |   .0850063   .0768226     1.11   0.269    -.0661384     .236151
Symptoms, signs and abn..  |   .0493855   .0848675     0.58   0.561    -.1175871    .2163582
Injury, poisoning and c..  |  -.1015919   .0985322    -1.03   0.303    -.2954493    .0922655
Factors influencing hea..  |   .1238484   .0922288     1.34   0.180    -.0576073    .3053041
                    Other  |  -.0453307   .0695495    -0.65   0.515     -.182166    .0915046
                           |
                   service |
PREVENIMS (preventive ..)  |   .0762168   .0761724     1.00   0.318    -.0736487    .2260823
      Dental consultation  |   .2069281    .123677     1.67   0.095    -.0364005    .4502567
                           |
                     _cons |  -1.388976   .1060743   -13.09   0.000    -1.597672    -1.18028
---------------------------+----------------------------------------------------------------
unidmed                    |
                var(_cons)|   .3686354   .0715938                         .2515654    .5401857
--------------------------------------------------------------------------------------------
*III) Facility-level Effects for Global_rating
(running melogit on estimation sample)

Survey: Mixed-effects logistic regression

Number of strata   =         1                 Number of obs     =     27,337
Number of PSUs     =       319                 Population size   = 68,852.018
                                               Subpop. no. obs   =     25,745
                                               Subpop. size      = 64,704.336
                                               Design df         =        318
                                               F(  22,    297)   =       3.23
                                               Prob > F          =     0.0000

--------------------------------------------------------------------------------------------
                           |             Linearized
             Global_rating |      Coef.   Std. Err.      t    P>|t|     [95% Conf. Interval]
---------------------------+----------------------------------------------------------------
                           |
                 age_quart |
           25-50 Quartile  |   .0346015   .0569551     0.61   0.544    -.0774549    .1466578
           50-75 Quartile  |   .2058203   .0556791     3.70   0.000     .0962744    .3153662
             Top Quartile  |   .1928738   .0613832     3.14   0.002     .0721052    .3136424
                           |
                  sex_male |   .0683643   .0380877     1.79   0.074    -.0065715       .1433
                           |
                 education |
 Completed Primary School  |   .1324671   .0525431     2.52   0.012     .0290911    .2358432
    High School or higher  |   .3057841   .0646895     4.73   0.000     .1785107    .4330575
                           |
                 icd10_bin |
Endocrine, nutritional ..  |    -.04062   .0574806    -0.71   0.480    -.1537103    .0724702
Diseases of the respira..  |  -.0532555   .0839624    -0.63   0.526    -.2184474    .1119364
Diseases of the digesti..  |   -.147092   .1004723    -1.46   0.144    -.3447665    .0505824
Diseases of the musculo..  |   .0169517   .0782781     0.22   0.829    -.1370566      .17096
Pregnancy, childbirth a..  |   .0855168   .0766488     1.12   0.265     -.065286    .2363196
Symptoms, signs and abn..  |   .0506526   .0848812     0.60   0.551     -.116347    .2176523
Injury, poisoning and c..  |  -.1017582   .0985484    -1.03   0.303    -.2956475    .0921311
Factors influencing hea..  |    .124955   .0923563     1.35   0.177    -.0567516    .3066615
```

```
                    Other |  -.0451579   .0695521    -0.65   0.517    -.1819983    .0916825
                          |
                  service |
PREVENIMS (preventive ..)  |   .0772609   .0762243     1.01   0.312    -.0727067    .2272285
      Dental consultation  |   .2051489   .1237612     1.66   0.098    -.0383453    .4486431
                          |
                fac_pop01 |  -.2575554    .413633    -0.62   0.534    -1.071358    .5562477
         fac_consultrms01 |   .3334109   .3959264     0.84   0.400    -.4455553    1.112377
             fac_diabetes |    .146368   .0892551     1.64   0.102    -.0292371     .321973
              fac_nurserm |   .0078173     .13793     0.06   0.955    -.2635534    .2791879
            fac_nutrition |   .0520806   .0898294     0.58   0.562    -.1246544    .2288157
                    _cons |  -1.455119   .1263892   -11.51   0.000    -1.703784   -1.206454
--------------------------+----------------------------------------------------------------
unidmed                   |
                var(_cons)|   .3628353   .0705424                      .2475066    .5319027
-------------------------------------------------------------------------------------------
*IV) Add RE for States for Global_rating
Note: Stage 1 is sampled with replacement; further stages will be ignored for variance
      estimation.

      pweight: <none>
          VCE: linearized
  Single unit: missing
     Strata 1: <one>
         SU 1: entidad
        FPC 1: <zero>
     Weight 1: state_weight
     Strata 2: <one>
         SU 2: unidmed
        FPC 2: <zero>
     Weight 2: facility_weight
     Strata 3: <one>
         SU 3: <observations>
        FPC 3: <zero>
     Weight 3: rescaled_patient_weights
(running melogit on estimation sample)

Survey: Mixed-effects logistic regression

Number of strata   =         1                Number of obs     =     27,337
Number of PSUs     =        32                Population size   = 68,852.018
                                              Subpop. no. obs   =     25,745
                                              Subpop. size      = 64,704.336
                                              Design df         =         31
                                              F(  22,     10)   =       6.24
                                              Prob > F          =     0.0024

-------------------------------------------------------------------------------------------
                          |             Linearized
            Global_rating |      Coef.   Std. Err.      t    P>|t|     [95% Conf. Interval]
--------------------------+----------------------------------------------------------------
                          |
                age_quart |
          25-50 Quartile  |   .0363162   .0554188     0.66   0.517    -.0767111    .1493436
          50-75 Quartile  |   .2075691   .0546569     3.80   0.001     .0960957    .3190425
            Top Quartile  |    .199244   .0628679     3.17   0.003     .0710241    .3274639
                          |
                 sex_male |   .0678541   .0436117     1.56   0.130    -.0210925    .1568007
                          |
                education |
 Completed Primary School  |   .1239821   .0543775     2.28   0.030     .0130785    .2348858
```

```
    High School or higher  |   .2998752   .0745267     4.02   0.000      .147877    .4518734
                           |
                 icd10_bin |
Endocrine, nutritional ..  |  -.0462069   .0662564    -0.70   0.491    -.1813378    .0889239
Diseases of the respira..  |  -.0563516   .0685583    -0.82   0.417    -.1961773     .083474
Diseases of the digesti..  |  -.1489402   .0897193    -1.66   0.107    -.3319239    .0340435
Diseases of the musculo..  |   .0131391   .0812024     0.16   0.873    -.1524743    .1787526
Pregnancy, childbirth a..  |   .0798801   .1043382     0.77   0.450     -.132919    .2926793
Symptoms, signs and abn..  |   .0395034   .0893947     0.44   0.662    -.1428183     .221825
Injury, poisoning and c..  |  -.1059502   .0933654    -1.13   0.265    -.2963702    .0844697
Factors influencing hea..  |   .1290214   .0980688     1.32   0.198    -.0709913    .3290341
                    Other  |  -.0471156   .0784928    -0.60   0.553    -.2072028    .1129716
                           |
                   service |
PREVENIMS (preventive ..)  |    .070673    .094898     0.74   0.462    -.1228729    .2642188
      Dental consultation  |   .1922784   .1431519     1.34   0.189    -.0996818    .4842387
                           |
                 fac_pop01 |   .1758157   .4477808     0.39   0.697    -.7374392    1.089071
         fac_consultrms01 |   .0418276   .3516141     0.12   0.906     -.675294    .7589493
              fac_diabetes |  -.0100024   .0922972    -0.11   0.914    -.1982437     .178239
               fac_nurserm |   -.083592   .0782994    -1.07   0.294    -.2432847    .0761008
             fac_nutrition |   .0955035   .1032436     0.93   0.362    -.1150632    .3060701
                     _cons |  -1.370369   .1658596    -8.26   0.000    -1.708642   -1.032096
---------------------------+----------------------------------------------------------------
entidad                    |
                 var(_cons)|   .2392245   .1050282                          .0977079    .5857086
---------------------------+----------------------------------------------------------------
entidad>unidmed            |
                 var(_cons)|   .2017519   .0374704                          .1381376    .2946617
--------------------------------------------------------------------------------------------
*V) Add state poverty level for Global_rating
(running melogit on estimation sample)

Survey: Mixed-effects logistic regression

Number of strata   =         1                Number of obs     =     27,337
Number of PSUs     =        32                Population size   = 68,852.018
                                              Subpop. no. obs   =     25,745
                                              Subpop. size      = 64,704.336
                                              Design df         =         31
                                              F(  23,      9)   =       5.88
                                              Prob > F          =     0.0046

--------------------------------------------------------------------------------------------
                           |             Linearized
             Global_rating |      Coef.   Std. Err.      t    P>|t|     [95% Conf. Interval]
---------------------------+----------------------------------------------------------------
                           |
                 age_quart |
            25-50 Quartile |   .0363813   .0554297     0.66   0.516    -.0766684    .1494309
            50-75 Quartile |   .2075904   .0546614     3.80   0.001     .0961077    .3190731
              Top Quartile |    .199295   .0628402     3.17   0.003     .0711316    .3274584
                           |
                  sex_male |   .0678951   .0436042     1.56   0.130    -.0210363    .1568264
                           |
                 education |
 Completed Primary School  |   .1239033   .0543969     2.28   0.030     .0129602    .2348464
    High School or higher  |   .2999678   .0745051     4.03   0.000     .1480136    .4519219
                           |
                 icd10_bin |
Endocrine, nutritional ..  |  -.0461836    .066243    -0.70   0.491    -.1812871      .08892
```

```
Diseases of the respira..  |  -.0563825   .0685725    -0.82   0.417     -.196237    .0834721
Diseases of the digesti..  |  -.1488815   .0896755    -1.66   0.107     -.331776    .0340129
Diseases of the musculo..  |   .0131726   .0811984     0.16   0.872    -.1524327    .1787778
Pregnancy, childbirth a..  |   .0799333   .1043205     0.77   0.449    -.1328296    .2926963
Symptoms, signs and abn..  |   .0396387   .0893295     0.44   0.660      -.14255    .2218274
Injury, poisoning and c..  |  -.1060049   .0933765    -1.14   0.265    -.2964475    .0844376
Factors influencing hea..  |   .1291362   .0980459     1.32   0.197    -.0708297    .3291021
                    Other  |  -.0470499    .078471    -0.60   0.553    -.2070927    .1129928
                           |
                   service |
PREVENIMS (preventive ..)  |   .0706781   .0948876     0.74   0.462    -.1228463    .2642026
      Dental consultation  |   .1923374   .1431533     1.34   0.189    -.0996257    .4843004
                           |
                 fac_pop01 |   .1758303   .4475757     0.39   0.697    -.7370065    1.088667
          fac_consultrms01 |   .0382857   .3508925     0.11   0.914    -.6773643    .7539357
              fac_diabetes |  -.0093208    .092181    -0.10   0.920    -.1973252    .1786835
               fac_nurserm |  -.0847935   .0783051    -1.08   0.287    -.2444979    .0749109
             fac_nutrition |   .0962518   .1028269     0.94   0.356    -.1134651    .3059686
           state_poverty01 |  -.3154306   .2843108    -1.11   0.276    -.8952863     .264425
                     _cons |  -1.233167   .2230705    -5.53   0.000    -1.688122   -.7782116
---------------------------+----------------------------------------------------------------
entidad                    |
                 var(_cons)|    .233501   .1086604                      .0903863    .6032187
---------------------------+----------------------------------------------------------------
entidad>unidmed            |
                 var(_cons)|   .2016778   .0374946                      .1380338    .2946665
--------------------------------------------------------------------------------------------
```
