# Supplementary material for: Factors associated with positive user experience with primary healthcare providers in Mexico: a multilevel modelling approach using national cross-sectional data
Source: BMJ Open. 2020 Jan 14;10(1):e029818. doi: 10.1136/bmjopen-2019-029818 (PMC7045193; doi:10.1136/bmjopen-2019-029818)
Supplement: Author's manuscript [file bmjopen-2019-029818.draft_revisions.pdf]

BMJ Open

BMJ Open is committed to open peer review. As part of this commitment we make the peer review history of every article we publish publicly available.

When an article is published we post the peer reviewers' comments and the authors' responses online. We also post the versions of the paper that were used during peer review. These are the versions that the peer review comments apply to.

The versions of the paper that follow are the versions that were submitted during the peer review process. They are not the versions of record or the final published versions. They should not be cited or distributed as the published version of this manuscript.

BMJ Open is an open access journal and the full, final, typeset and author-corrected version of record of the manuscript is available on our site with no access controls, subscription charges or pay-per-view fees (http://bmjopen.bmj.com).

If you have any questions on BMJ Open's open peer review process please email info.bmjopen@bmj.com

BMJ Open

Factors associated with positive user experience with primary healthcare providers in Mexico: A multi-level modeling approach

Journal:	*BMJ Open*
Manuscript ID	bmjopen-2019-029818
Article Type:	Research
Date Submitted by the Author:	13-Feb-2019

Complete List of Authors:	Holt, Kelsey; University of California, San Francisco,<br>Doubova, Svetlana; Mexican Institute of Social Security, Epidemiology and Health Services Research Unit, CMN Siglo XXI<br>Lee, Dennis; Harvard T. H. Chan School of Public Health, Global Health and Population<br>Perez-Cuevas, Ricardo; Secretaria de Salud, Coordinación de Asesores; Hospital Infantil de Mexico Federico Gomez, Centro de Estudios Economicos y Sociales en Salud<br>Leslie, Hannah; Harvard T. H. Chan School of Public Health, Global Health and Population

Keywords:	PRIMARY CARE, Quality in health care < HEALTH SERVICES ADMINISTRATION & MANAGEMENT, PUBLIC HEALTH

SCHOLARONE™
Manuscripts

Factors associated with positive user experience with primary healthcare providers in Mexico: A multi-level modeling approach

Kelsey Holt, ScD, University of California, San Francisco; San Francisco, United States

Svetlana V. Doubova, MD, DSc, Mexican Institute of Social Security; Mexico City, Mexico

Dennis Lee, Harvard T.H. Chan School of Public Health; Boston, Massachusetts

Ricardo Perez Cuevas, DSc , Institute of Public Health in Mexico; Morelos, Mexico

Hannah H. Leslie, PhD, Harvard T.H. Chan School of Public Health; Boston, Massachusetts

Corresponding author: Kelsey Holt, 1001 Potrero Ave, San Francisco, CA, 94110; Kelsey.holt@ucsf.edu; 415-206-4234; fax: N/A

Key words: primary care, patient centered care, mexico

Word count: 3,511

For peer review only

ABSTRACT

Objective: To investigate factors associated with patient experience with primary care in a large public health system in Mexico.

Methods: We analyzed a cross-sectional 2016 national satisfaction survey conducted by the Mexican Social Security Institute with beneficiaries over age 18. Patient-level data were merged with facility-level data and information on poverty by state. We defined patient experience measures as communication, opportunity to share health concerns, resolution of doubts, and overall treatment, and assessed the relationship of patient, facility, and state factors with patient experience using random effects logistic regression.

Results: 25,745 patients' responses from 319 primary care facilities were analyzed. The majority had positive experiences, including good communication (78%), the opportunity to share health concerns (91%), and resolution of doubts about treatment (85%). 29% of visits were rated as excellent overall. Differences between facilities accounted for 11.9-19.8% of the variation in patient experience for the four measures assessed. In adjusted models, larger patient population was correlated with worse reported communication while presence of a diabetes program was associated positively with communication. Males reported more positive communication than females; older individuals tended to report more positive experiences overall, and more highly educated individuals were less likely to report positive communication but more likely to have overall positive experiences. Preventive care visits were generally rated higher than curative visits.

Conclusion: Primary care quality improvement might have the potential to bolster individual experiences with care, given that up to one-fifth of the variation in experience was attributable to facilities. The relationship between individual characteristics and experience ratings reinforces the importance of patients' expectations of care and the potential for differential treatment by providers to impact experience.

Strengths and limitations of this study

- To the best of our knowledge, this is the first study to examine factors associated with positive user experience with primary care in a representative sample from a middle-income country.
- This study uses multi-level modeling techniques to examine the variance in user experience attributable to factors at the individual versus facility levels.
- Data are cross-sectional and thus we are unable to draw causal inferences from the observed associations.
- Findings are not generalizable to other sectors of the Mexican healthcare system, outside of the Mexican Institute of Social Security (IMSS).

For peer review only - http://bmjopen.bmj.com/site/about/guidelines.xhtml

INTRODUCTION

High quality primary care is essential to countries' ability to achieve Universal Health Coverage by 2030, one of the United Nations Sustainable Development Goals (SDGs) established in 2015.[1, 2] The core values of primary care, including person-centeredness and health equity, are in line with the SDGs' orientation towards reducing inequities and achieving good health and well-being for all:[1, 3] primary care provides a platform for delivering care that meets the entire populations' needs.[2]

In order to ensure that primary care is high quality and able to achieve the promise of promoting health for all, monitoring patient experiences is critical. Positive user experience is a core component of high quality health care and contributes to the population's confidence in the health system and achieving better health outcomes.[4] For instance, positive user experiences with primary care have been found to be associated with public perception of high healthcare quality,[5] self-rated health,[6] and high patient satisfaction.[7] Reviews of patient experiences in both primary and secondary care have found associations with clinical effectiveness and patient safety [8] and patient satisfaction.[9]

User experiences are the products of different patient and health services-related factors. Research examining the impact of health services-related factors on patient experience of care must account for the hierarchical nature of data: patients nested within providers, nested within institutions, nested within geographies.[10] Some studies, primarily from high-income countries, have examined the association between factors at different levels and patient experiences. At the individual level, age, education, and health status are the most common characteristics associated with patient experiences, with older and healthier individuals, and those with less education, tending to report more positive experiences.[11] Further, expectations for care play a clear role in overall satisfaction with care such that low expectations are associated with higher satisfaction.[9] Patient experience measures are increasingly recognized as valuable for quality improvement purposes as they are known to discriminate more effectively between practices than patient satisfaction measures.[4, 12] However, low expectations likely still influence to some degree the ways in which individuals report experiencing care.

At the health services level, variables such as presence of specific types of providers (e.g., nurses, in addition to physicians), health facility structure (e.g., fewer number of patients per provider, fewer numbers of physicians per practice) and functioning (e.g., long weekly working hours, non-training versus training facilities, and operational agreements with other health systems) have been found to be associated with positive user experience with primary care.[13-15]

Much of this research is based in high-income settings. Similar research in lower-resource settings would provide crucial information to guide decision-makers in how to allocate resources to address deficiencies in system responsiveness to patients, and to the public to empower them to demand more patient-centered care. Further, in order to understand the degree to which experience measures are reliable indicators of the performance of healthcare systems (rather than a more limited indicator of variability in provider-patient relationships by individual-level socio-

For peer review only - http://bmjopen.bmj.com/site/about/guidelines.xhtml

demographic factors), it is critical to conduct analyses on the variability of patient experience attributable to health services factors.

Consistent with global initiatives, Mexico has a strong national commitment to both primary care and quality improvement to ensure the promises of universal access are achieved.[16, 17] Since 2009, user satisfaction/experience surveys are regular practice for public health providers in México. The Mexican Institute of Social Security (IMSS) is the largest provider, covering more than 63 million formal labor market employees and their families.[18] IMSS conducts annual National Satisfaction Surveys; yet, their results have not been analyzed comprehensively.

In this paper, we take advantage of this robust data collection effort by IMSS to investigate patient, facility, and geographic factors influencing experience with health care in the IMSS primary care system, and the degree to which variability in patient experience is due to patient-level versus facility-level factors.

METHODS

Data sources

Our primary data set comes from the November 2016 IMSS National Satisfaction Survey (ENSAT). IMSS regularly conducts these cross-sectional surveys with its beneficiaries over 18 years old at primary, secondary, and tertiary care services. This analysis focuses on patients at the primary care level. The survey's sampling design consists of a two-stage stratified probabilistic sampling where the probability of selecting a health facility is defined by region and facility size. The number of patients to be interviewed in each facility is estimated according to the average number of daily consultations with physicians and the proportion of patients satisfied in the previous survey. Patient recruitment is done through systematic sampling, with a random starting point and a fixed interval based on the estimated sample size and the average number of daily consultations in the clinic. A private firm (Berumen and Associates) conducted the survey. Trained interviewers carried out direct, structured interviews with IMSS health services users. The interviewers used a satisfaction questionnaire that the Center for Evaluation Research and Surveys of the National Institute of Public Health had previously validated. The response rate in the survey was 87%.

To perform the analysis, we merged 2016 ENSAT database with a facility-level database of a 2016 physical inventory of health care facilities and a 2016 record of patients affiliated with IMSS in each facility. Finally, a 2014 database of the National Council for the Evaluation of Social Development Policy (Spanish acronym CONEVAL) on the percentage of the population in each state living in poverty was merged into patient-level data.[19] Poverty is calculated by CONEVAL as a multidimensional measure based on criteria related to housing, household services, education, health care, social security, food, and per capita income.

Patient experience outcomes

We examined four binary dependent variables. The first is a composite measure of verbal and non-verbal communication, designated as positive if the patient answered favorably to three binary questions (translated from Spanish): "Did the staff who attended to you make eye contact while greeting you?"; "Did the staff who attended to you listen to you with attention and without

For peer review only - http://bmjopen.bmj.com/site/about/guidelines.xhtml

interruptions?"; and "Did the staff who attended to you respond clearly to your questions?" We also examined the questions: "Did you have the opportunity to tell the doctor or nurse what was bothering you about your health?" (referred to hereafter as "opportunity to share health concerns"); "Did you end up with doubts about the treatments necessary for your condition?" (reverse coded and referred to hereafter as "resolution of doubts"); and "How was the treatment you received in this facility during today's visit?", scored on a scale of 1-5 with 1 being "excellent," and 5 being "Terrible," collapsed to Excellent versus all other responses and referred to hereafter as the global measure.

Explanatory variables

In identifying independent variables to include in our models, we conceptualized patient experiences with IMSS healthcare delivery as being potentially influenced by factors at multiple levels (Figure 1). Available individual-level variables in the IMSS dataset included age (categorized into quartiles); sex (binary); and education level (high school or higher, less than high school, and less than primary school). We hypothesized that patients' age, sex, and educational status will explain some variance in reported experience due to differential treatment by providers and different expectations for care based on these factors. We also hypothesized experiences would vary by the primary reason for the visit, independent of patient characteristics, given that the nature of what is covered in a visit likely influences how providers approach patients (for example, primary care doctors are less comfortable discussing stigmatized issues such as sexual health and therefore exhibit poorer communication skills) and how well the visit can meet the patient's needs (for example, if their issue has numerous treatment options they may have a better experience than for those issues where treatments are limited). We therefore included the variables of self-reported reason for visit, collapsed from 149 categories into 10 categories based on ICD-10 codes, and type of consultation (curative visit, preventive care visit at the PREVENIMSS module at each clinic,[20] or dental visit). PREVENIMSS is a Spanish acronym for "Integrated Preventive Care Program," an initiative that provides targeted preventive care services to individuals based on their age and gender.

[Figure 1]

At the facility level, we hypothesized that size of facility could impact processes of care and included two variables that served as proxies for size: total number of rooms for all types of services and the number of people affiliated with a primary care clinic. We also included variables related to the presence or absence of programs that are designed to improve quality of care: presence of a special diabetes program comprising multidisciplinary teams of health-professionals (DIABETIMSS); presence of nurses' rooms attached to family doctors' consultation rooms; and availability of a nutritionist. Finally, we hypothesized that experience would vary by the percentage of population in a state that is living in poverty, given that this is likely reflective of the infrastructure in the state—including healthcare infrastructure. We thus included poverty rate in the facility's region as an independent variable. Continuous variables (consultation rooms, number of affiliates, poverty rate) were rescaled to fall on a scale of minimum 0 to maximum 1 to facilitate comparison across explanatory variables.

Analysis

For peer review only - http://bmjopen.bmj.com/site/about/guidelines.xhtml

We constructed two-level random-intercept logistic regression models for each dependent variable, treating facility as a random effect with patients nested within facilities. Intra-class correlation (ICC) coefficients were calculated to determine the amount of the total variance in the outcome due to differences at each level. We then added independent variables at patient and facility levels as fixed effects, retaining all variables in the final model due to the exploratory nature of the study. Random intercept models provide inference on the within-cluster difference associated with each covariate. For facility-level variables, in order to interpret these effects as differences between facilities, we present population-average coefficients odds ratio, the average odds ratio comparing subjects from different clusters that have identical observed values for other covariates. We also examined interval odds ratio (IOR) to compare the magnitude of each higher-level covariate to the random effect. It provides the range of odds ratios comparing individuals with a one unit difference on the facility covariate from the 10th percentile of the random effect to the 90th percentile.[21, 22] Stata version 15 was used for all analyses.

The IMSS dataset included weights to account for non-response. To account for missing data, we weighted complete cases based on inverse probability weights. Weights were calculated as the inverse of the probability of being a complete case predicted from regressing a binary indicator of completeness on patient-level covariates with no missing values (all except reason for visit and education level).

Ethical review

This secondary analysis of de-identified data was approved by IMSS Research and Ethics Committee (No. R 2018-785-037).

Patient and public involvement

Patients nor the public were involved in the design, conduct, or analysis of this study.

RESULTS

A total of 25,745 patients´ responses from 319 primary care facilities were analyzed. 1,593 cases were dropped for missing data. The median age of respondents was 49 years, 71% were female, and 70% had less than a high school education (Table 1). A small minority of the visits represented preventive (8%) or dental (2%) care, versus curative (90%). The most common reasons for the visit were diseases of the circulatory system (20%) or endocrine, nutritional, and metabolic diseases (19%). At the facility level, the average number of rooms in clinics was 20 (range=1-57), and the median number of enrolled patients was 83,691 (range=2,356-468,618). A minority had nurses rooms attached (6%), the DIABETIMSS program (19%), or nutritionists (39%). Participants lived in regions where 41% of the population, on average, lived in poverty.

[Table 1]

The majority of patients reported the staff greeted them by looking them in their eye (82%), responded clearly to their questions (92%), and listened to them with attention and without interruptions (91%) (Figure 2). 78% reported experiencing all three of these indicators of good verbal or non-verbal communication. 91% reported having the opportunity to share health concerns; 85% reported ending up with no doubts about their treatment. In 29% of visits they reported excellent care overall (Figure 3).

For peer review only - http://bmjopen.bmj.com/site/about/guidelines.xhtml

[Figure 2]

[Figure 3]

Examining ICC values, the amount of individual variation in propensity to report positive experiences that was due to systematic differences between facilities rather than systematic differences between patients varied by outcome and ranged from 19.8% in the case of opportunity to share health concerns to 11.9% for verbal/non-verbal communication (Table 2). These figures did not change more than a percentage point after adjustment for individual and facility characteristics, with the exception of opportunity to share health concerns, which decreased to 17.0% of residual variation due to systematic differences between facilities rather than between patients (data not shown).

[Table 2]

In the multivariable models predicting positive patient experiences with all patient- and facility-level covariates included, individuals within the same facility had higher odds of reporting positive communication if they were male (AOR 1.15, 95% CI 1.06, 1.26), 63-99 years compared to 18-33 years of age (AOR 1.23, 95%CI 1.06 – 1.44), attended a visit related to pregnancy, childbirth, or the post-partum period (AOR 1.37, 95% CI 1.11, 1.70), or were seeking dental services (AOR 1.48, 95% CI 1.08, 2.02) after adjusting for other individual and facility characteristics. More educated patients (with high school education or higher compared to less than primary care) within the same facility had lower odds of reporting high-quality communication, controlling for other covariates (AOR 0.82, 95% CI 0.71, 0.94).

The pattern for other outcomes was slightly different. Individuals within the same facility had lower odds of reporting they were given the opportunity to share health concerns if they were 34-49 and 50-62 years compared to 18-33 years (AOR 0.80 and 0.81, 95% CI 0.67, 0.96 and 0.68, 0.97; respectively); being seen for visits categorized in ICD-10 codes as injury, poisoning and other consequences of external causes (AOR 0.65, 95% CI 0.51, 0.83) or "factors influencing health status or contact with health services" (0.44, 95% CI 0.34, 0.56); or if they were being seen in a preventive care versus curative visit (AOR 0.27, 95% CI 0.22, 0.33). Individuals within the same health facility had lower odds of reporting resolution of doubts if they were being seen for symptoms, signs and abnormal clinical and laboratory findings, not elsewhere classified (AOR 0.60, 95% CI 0.49, 0.74), being seen for injury, poisoning and other consequences of external causes (AOR 0.76, 95% CI 0.59, 0.99), or other reason (AOR 0.70, 95% CI 0.59, 0.84). Patients within the same facility in preventive care visits had higher odds of ending up without doubts, controlling for other covariates (AOR 1.33, 95% CI 1.04, 1.71). Finally, individuals within the same facility had higher odds of reporting overall excellent care if they were 50-62 and 63-99 years compared to 18-33 years (AOR 1.21 and 1.20, 95% CI 1.07, 1.37 and 1.05, 1.37; respectively) or if they had a primary school or high school education compared to less than primary school (AOR 1.14 and 1.35, 95% CI 1.02, 1.28 and 1.17, 1.56; respectively).

Across all facilities, larger patient population size affiliated with clinic was associated with lower odds of positive communication in the adjusted model (AOR comparing the smallest population size to the largest 0.35, 95% CI 0.22, 0.60). Across all facilities, those with an integrated diabetes program had significantly higher odds of reporting good communication (AOR 1.24, 95% CI 1.04, 1.48), and those in states with more poverty were less likely to report having the

For peer review only - http://bmjopen.bmj.com/site/about/guidelines.xhtml

opportunity to share health concerns, although this association was not statistically significant at $p<0.05$ (AOR 0.58, 95% CI 0.34, 1.00). No IORs excluded the null, suggesting no evidence that any measured facility-level characteristics play a more significant role in explaining variability than the overall random effect.

DISCUSSION

This novel analysis of patient experiences with primary care in Mexico identified generally positive perspectives on health care and found that up to one fifth of variation in experience occurs at the facility level. This suggests that quality improvement at the facility level has the potential to improve individual experiences with care. While a few facility characteristics were associated with better patient experience, much of the facility-level variation could not be explained by measured covariates, suggesting further research is necessary to identify levers for improvement. In-depth exploration of high-performing facilities would also help provide insights into what it is about the facility environment that influences patient experience. Individual characteristics shaped experience ratings, reinforcing the importance of patients' expectations of care and the potential for differential treatment by providers to impact experience. Systematic assessment of patient expectations and provider bias may help to inform health system improvements.

The variable with most variance attributable to the facility level was opportunity to share health concerns, suggesting that interventions specifically targeted at making sure providers have training and necessary time to elicit patient input during consultations—a key tenet of patient-centeredness[23]—maybe be particularly promising.

The degree of variation we uncovered at the facility level is similar to that identified in a study in general practice in Canada, which found that 20% of variance in perception of accessibility was attributable to practice-level characteristics;[15] and it is lower than that identified in a patient survey among primary care patients in California, United States, which found that 28-48% was due to system related factors.[24]

Larger patient population affiliated with clinic was correlated with worse reported communication. This is consistent with findings from other research [14] and suggests that particular attention may need to be paid to improving patient experience in settings with high patient volume. The presence of the special diabetes program, DIABETIMSS, also was associated positively with patient experience of communication, even controlling for the reason for a patient's visit. The presence of the program may be a proxy for other unmeasured characteristics about facilities related to their commitment to quality improvement or patient experience.

The pattern of relationships between individual-level characteristics and patient experience was variable across outcomes. Males reported more positive communication than females; this may reflect a difference in how providers communicate with males or different expectations for care.[25] Older individuals tended to report more positive experiences overall and specifically with communication, consistent with a review of the impact of individual characteristics on experiences.[11] Individuals between 34-62 years old were less likely than those between 18-33

For peer review only - http://bmjopen.bmj.com/site/about/guidelines.xhtml

years to report opportunity to share their health concerns, again either representing a difference in communication style on the part of providers or different expectations for care by patients by age. In terms of education, more educated individuals were less likely to report positive communication, consistent with other research and likely due to higher expectations for care.[11] However, they were more likely to report positive overall experiences. This could represent the fact that those with the most education are empowered to be more "active" participants in their care,[26] or possible provider discrimination against patients with less than primary school.

The reason for a patient's visit was consistently related to their experience, with preventive care visits eliciting higher ratings for opportunity to share health concerns and resolution of doubts, and dental visits being associated with better communication. This may reflect the different nature of preventive care, wherein patient engagement about priorities and concerns is more of a focus than when patients are being seen for a particular medical concern. It could, however, reflect better quality care for patients in the preventive care or dental modules. Specific conditions were also associated with report of better experiences in some cases, also likely reflecting either the nature of the visit or systematic differences in the ways providers treat patients who are being seen for different conditions.

These findings should be considered in light of several limitations. First, the data are cross-sectional and we are unable to draw causal inferences from the observed associations between independent and dependent variables. Further, though we were able to identify the overall amount of variance attributable to the facility level, we had few potential explanatory factors available at the facility level. Second, available datasets did not indicate which providers patients had seen, keeping us from being able to parse the variance in patient experience attributable to differences between providers. Future efforts to collect national experience data in the IMSS system could benefit from collecting these data. Third, we did not have access to information on all IMSS primary care visits to be able to compare the demographics of our sample with the overall population of users. However, the response rate was high (87%), suggesting adequate representativeness. Finally, the results are generalizable only to users of the IMSS system, as the survey did not include patients from other health sectors in Mexico.

Our findings shed light on the influences at both individual and facility levels on patients experiences with a large public health system in Mexico. These findings can inform efforts to improve patient experiences, thereby promoting confidence in the health system and better health outcomes. Key priorities for future work include further investigation of the facility-level factors accounting for differences in patient experience, as well as more robust monitoring of patients' expectations for care and their potential experiences with bias to better understand what accounts for individual level differences in experience.

For peer review only - http://bmjopen.bmj.com/site/about/guidelines.xhtml

Table 1. Patient and facility characteristics

	n (%)
**Patient characteristics (N=25,745)**	
Sex	
Male	7,533 (29.3)
Female	18,212 (70.7)
Age	
Median, Range (Interquartile range)	49, 18-99 (34-63)
Level of education	
Less than primary school	4,006 (15.6)
Less than high school	13,914 (54.0)
High school or more	7,825 (30.4)
Visit type	
Curative	23,144 (89.9)
Preventive	2,024 (7.9)
Dental	577 (2.2)
Reason for visit	
Diseases of the circulatory system	5,132 (19.9)
Endocrine, nutritional and metabolic diseases	4,898 (19.0)
Other	3,085 (12.0)
Diseases of the respiratory system	2,386 (9.3)
Factors influencing health status and contact with health services	1,805 (7.0)
Pregnancy, childbirth and the puerperium	1,781 (6.9)
Diseases of the musculoskeletal system and connective tissue	1,710 (6.6)
Diseases of the digestive system	1,691 (6.6)
Symptoms, signs and abnormal clinical and laboratory findings, not elsewhere classified	1,664 (6.5)
Injury, poisoning and certain other consequences of external causes	1,593 (6.2)
Percent poverty in region	
Mean (Range), SD	41.1 (20.4-76.2), 12.9
**Facility characteristics (N=319)**	
Number of consultation rooms	
Mean (Range), SD	20.4 (1-57), 13.0
Diabetes program	
No diabetes program	258 (80.9)
Has diabetes program	61 (19.1)
Nutritionist	
No nutritionist	194 (60.8)
At least one nutritionist	125 (39.2)
Nurses room	

For peer review only - http://bmjopen.bmj.com/site/about/guidelines.xhtml

No nurse room	300 (94.0)
At least one nurse room	19 (6.0)
Patient population size	
Median (Range), SD	83,691 (2,356-468,618), 86,231.0

For peer review only

For peer review only - http://bmjopen.bmj.com/site/about/guidelines.xhtml

Table 2. Multi-level logistic regression results: odds ratios with 95% confidence intervals (n=25,745 patients, N= 319 facilities)

	Positive verbal/non-verbal communication		Opportunity to share health concerns		Resolution of doubts		Excellent global experience rating	
**Individual Level Variables**	AOR (95% CI)	p-value	AOR (95% CI)	p-value	AOR (95% CI)	p-value	AOR (95% CI)	p-value
Male sex	**1.15 (1.06, 1.26)**	**.002**	1.12 (.97, 1.31)	.128	.97 (.87, 1.09)	.639	1.05 (.97, 1.14)	.203
Age								
18-33 years	Ref		Ref		Ref		Ref	
34-48 years	1.07 (.95, 1.20)	.281	**.80 (.67, .96)**	**.016**	.91 (.78, 1.06)	.216	.98 (.86, 1.12)	.804
49-62 years	1.13 (1.00, 1.28)	.056	**.81 (.68, .97)**	**.024**	.98 (.83, 1.15)	.770	**1.21 (1.07, 1.37)**	**.002**
63-99 years	**1.23 (1.06, 1.44)**	**.008**	.80 (.64, 1.01)	.057	.94 (.79, 1.12)	.504	**1.20 (1.05, 1.37)**	**.009**
Level of education								
Less than primary school	Ref		Ref		Ref		Ref	
Less than high school	.89 (.77, 1.02)	.093	1.05 (.88, 1.25)	.592	1.03 (.90, 1.18)	.620	**1.14 (1.02, 1.28)**	**.020**
High school or more	**.82 (.71, .94)**	**.006**	1.17 (.95, 1.43)	.140	1.15 (.99, 1.35)	.075	**1.35 (1.17, 1.56)**	**<.0001**
Visit type								
Curative	Ref		Ref		Ref		Ref	
Preventive	.98 (.82, 1.17)	.833	**.27 (.22, -.33)**	**<.0001**	**1.33 (1.04, 1.71)**	**.024**	1.10 (.93, 1.29)	.269
Dental	**1.48 (1.08, 2.02)**	**.015**	1.04 (.67, 1.61)	.875	1.05 (.73, 1.50)	.808	1.22 (.95, 1.58)	.121
Reason for visit								
Diseases of the circulatory system	Ref		Ref		Ref		Ref	
Endocrine, nutritional and metabolic diseases	1.05 (.91, 1.21)	.473	1.06 (.83, 1.37)	.631	.93 (.78, 1.11)	.435	.91 (.80, 1.03)	.134
Other	.92 (.79, 1.07)	.289	.92 (.72, 1.19)	.533	**.70 (.59, .84)**	**<.0001**	.94 (.81, 1.09)	.399
Diseases of the respiratory system	.94 (.80, 1.10)	.441	1.21 (.90, 1.62)	.205	1.05 (.85, 1.28)	.665	.94 (.77, 1.14)	.501
Factors influencing health status and contact with health services	1.07 (.85, 1.35)	.579	**.44 (.34, .56)**	**<.0001**	1.06 (.78, 1.43)	.723	1.15 (.95, 1.40)	.159
Pregnancy, childbirth and the puerperium	**1.37 (1.11, 1.70)**	**.004**	1.07 (.79, 1.46)	.663	.94 (.75, 1.17)	.577	1.04 (.88, 1.23)	.647

For peer review only - http://bmjopen.bmj.com/site/about/guidelines.xhtml

Diseases of the musculoskeletal system and connective tissue	.83 (.67, 1.02)	.070	.91 (.67, 1.23)	.520	.80 (.60, 1.06)	.119	1.00 (.84, 1.21)	.963
Diseases of the digestive system	.88 (.70, 1.11)	.279	.99 (.74, 1.33)	.937	.86 (.67, 1.10)	.234	.86 (.68, 1.08)	.182
Symptoms, signs and abnormal clinical and laboratory findings, not elsewhere classified	.88 (.71, 1.10)	.253	.92 (.68, 1.24)	.572	**.60 (.49, .74)**	**<.0001**	1.00 (.84, 1.20)	.956
Injury, poisoning and certain other consequences of external causes	.89 (.73, 1.08)	.227	**.65 (.51, .83)**	**.001**	**.76 (.59, .99)**	**.042**	.87 (.69, 1.10)	.256
**Facility Level Variables**	AOR (95% CI) or IOR	p-value	AOR (95% CI) or IOR	p-value	AOR (95% CI) or IOR	p-value	AOR (95% CI) or IOR	p-value
Number of consultation rooms								
AOR	1.08 (.57, 2.04)	.820	.66 (.28, 1.54)	.331	.58 (.27, 1.24)	.162	1.39 (.57, 3.39)	.470
IOR	0.34, 3.44		0.14, 2.78		0.15, 2.09		0.34, 6.06	
Presence of:								
Diabetes program								
AOR	**1.23 (1.03, 1.47)**	**.021**	1.19 (.99, 1.44)	.091	1.09 (.91, 1.32)	.355	1.16 (.98, 1.37)	.078
IOR	0.39, 3.97		0.27, 5.36		0.29, 4.15		0.27, 4.97	
Nutritionist								
AOR	.91 (.80, 1.04)	.164	.93 (.79, 1.10)	.392	1.06 (.90, 1.25)	.471	1.08 (.91, 1.28)	.385
IOR	0.28, 2.87		0.21, 4.09		0.28, 4.02		0.25, 4.59	
Nurses room								
AOR	.85 (.64, 1.11)	.235	.96 (.72, 1.29)	.790	.85 (.64, 1.14)	.287	1.00 (.78, 1.30)	.979
IOR	0.26, 2.66		0.22, 4.25		0.22, 3.18		0.24, 4.23	
Percent poverty in region (minimum = 20%; versus maximum = 76%)								

For peer review only - http://bmjopen.bmj.com/site/about/guidelines.xhtml

	AOR	1.16 (.74, 1.80)	.514	.60 (.35, 1.02)	.060	.91 (.60, 1.37)	.651	.78 (.51, 1.20)	.261
	IOR	0.37, 3.71		0.13, 2.50		0.24, 3.40		0.18, 3.23	
Patient population size									
	AOR	**.31 (.15, .66)**	**.003**	.43 (.17, 1.06)	.067	.60 (.27, 1.34)	.213	.79 (.35, 1.82)	.585
	IOR	0.09, 0.92		0.09, 1.72		0.15, 2.18		0.18, 3.27	
**Intraclass correlation between facilities**		11.9%		19.8%		14.7%		16.4%	

For peer review only - http://bmjopen.bmj.com/site/about/guidelines.xhtml

Author statement: KH, SD, and HL conceived of the study; KH, HL, and DL conducted the analysis; KH drafted the manuscript; SD, HL, DL, and RPC provided substantive feedback on the manuscript.

Funding statement: HL and DL received support from the Bill and Melinda Gates Foundation (Award OPP1161450, Kruk)

Acknowledgements: The authors would like to acknowledge the generous help of Sarah Wulf in preparing the tables and figures for the manuscript.

Competing interests: We have no competing interests to declare.

For peer review only

REFERENCES CITED

1. Pettigrew, L.M., et al., *Primary health care and the Sustainable Development Goals.* The Lancet, 2015. **386**(10009): p. 2119-2121.
2. Stigler, F.L., et al., *No universal health coverage without primary health care.* The Lancet, 2016. **387**(10030): p. 1811.
3. Van Lerberghe, W., *The world health report 2008: primary health care: now more than ever.* 2008: World Health Organization.
4. Kruk, M.E., et al., *High-quality health systems in the Sustainable Development Goals era: time for a revolution.* The Lancet Global Health, 2018.
5. Doubova, S.V., et al., *Attributes of patient-centered primary care associated with the public perception of good healthcare quality in Brazil, Colombia, Mexico and El Salvador.* Health policy and planning, 2016. **31**(7): p. 834-843.
6. Guanais, F., et al., *Patient-centered primary care and self-rated health in 6 Latin American and Caribbean countries: Analysis of a public opinion cross-sectional survey.* PLoS medicine, 2018. **15**(10): p. e1002673.
7. Birhanu, Z., et al., *Determinants of satisfaction with health care provider interactions at health centres in central Ethiopia: a cross sectional study.* BMC health services research, 2010. **10**(1): p. 78.
8. Doyle, C., L. Lennox, and D. Bell, *A systematic review of evidence on the links between patient experience and clinical safety and effectiveness.* BMJ open, 2013. **3**(1): p. e001570.
9. Batbaatar, E., et al., *Determinants of patient satisfaction: a systematic review.* Perspectives in public health, 2017. **137**(2): p. 89-101.
10. Hearld, L.R., et al., *How do hospital organizational structure and processes affect quality of care? A critical review of research methods.* Medical Care Research and Review, 2008. **65**(3): p. 259-299.
11. Sandager, M., M. Freil, and J.L. Knudsen, *Please tick the appropriate box: Perspectives on patient reported experience.* Patient Experience Journal, 2016. **3**(1): p. 63-79.
12. Salisbury, C., M. Wallace, and A.A. Montgomery, *Patients' experience and satisfaction in primary care: secondary analysis using multilevel modelling.* Bmj, 2010. **341**: p. c5004.
13. Cohidon, C., P. Wild, and N. Senn, *Patient experience in primary care: association with patient, physician and practice characteristics in a fee-for-service system.* Swiss medical weekly, 2018. **148**.
14. Heje, H.N., et al., *Doctor and practice characteristics associated with differences in patient evaluations of general practice.* BMC health services research, 2007. **7**(1): p. 46.
15. Haggerty, J.L., et al., *Practice features associated with patient-reported accessibility, continuity, and coordination of primary health care.* The Annals of Family Medicine, 2008. **6**(2): p. 116-123.
16. Atun, R., et al., *Health-system reform and universal health coverage in Latin America.* The Lancet, 2015. **385**(9974): p. 1230-1247.
17. Doubova, S.V., et al., *Quality governance in a pluralistic health system: Mexican experience and challenges.* The Lancet Global Health, 2018. **6**(11): p. e1149-e1152.
18. Gobierno de los Estados Unidos Mexicanos. Cuarto Informe de Gobierno 2015-2016. Anexo estadístico. Ciudad de México 2016. Available at: http://www.presidencia.gob.mx/cuartoinforme/
19. Consejo Nacional de Evaluación de la Política de Desarrollo Social. Información de pobreza y evaluación en las entidades federativas. Mexico: CONEVAL, 2014. Available at http://www.coneval.org.mx/coordinacion/entidades/Paginas/inicioent.aspx
20. Gutiérrez, G., et al., *Strengthening preventive care programs: a permanent challenge for healthcare systems; lessons from PREVENIMSS México.* BMC public health, 2010. **10**(1): p. 417.

For peer review only - http://bmjopen.bmj.com/site/about/guidelines.xhtml

21. Austin, P.C. and J. Merlo, *Intermediate and advanced topics in multilevel logistic regression analysis.* Statistics in medicine, 2017. **36**(20): p. 3257-3277.
22. Larsen, K. and J. Merlo, *Appropriate assessment of neighborhood effects on individual health: integrating random and fixed effects in multilevel logistic regression.* American journal of epidemiology, 2005. **161**(1): p. 81-88.
23. Mead, N. and P. Bower, *Patient-centredness: a conceptual framework and review of the empirical literature.* Soc Sci Med, 2000. **51**(7): p. 1087-110.
24. Rodriguez, H.P., et al., *Attributing sources of variation in patients' experiences of ambulatory care.* Medical care, 2009: p. 835-841.
25. Roder-DeWan, S., et al., *Good ratings for poor quality: Low healthcare expectations among internet users in 12 low- and middle-income countries*. In Press
26. Leonard, K.L., Active patients *in rural African health care: implications for research and policy.* Health policy and planning, 2013. **29**(1): p. 85-95.

For peer review only

Figure 1. Conceptual model of relationship between model variables and reported experience with provider (N=25,745 patients and 319 facilities)

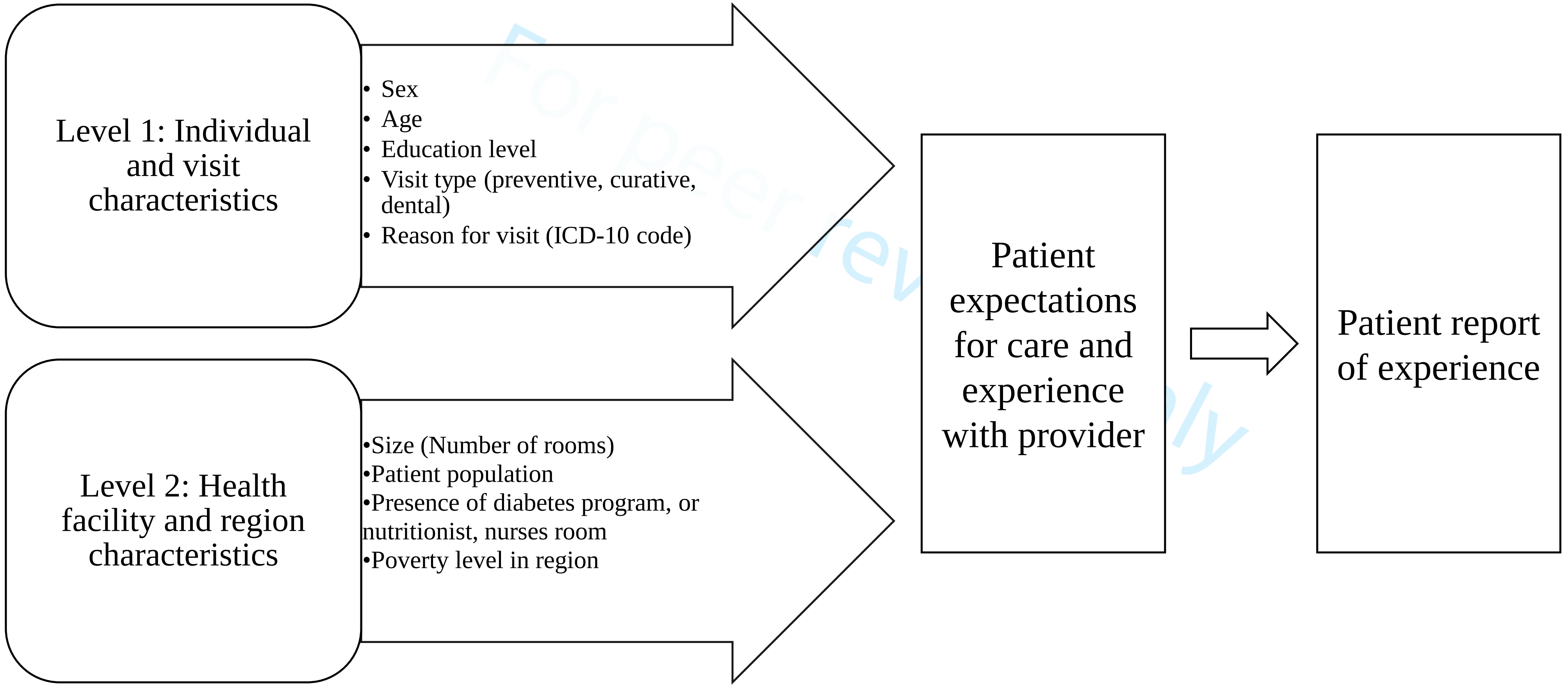

Figure 2. Patient report of experience with communicating with health care providers (N=25,745)

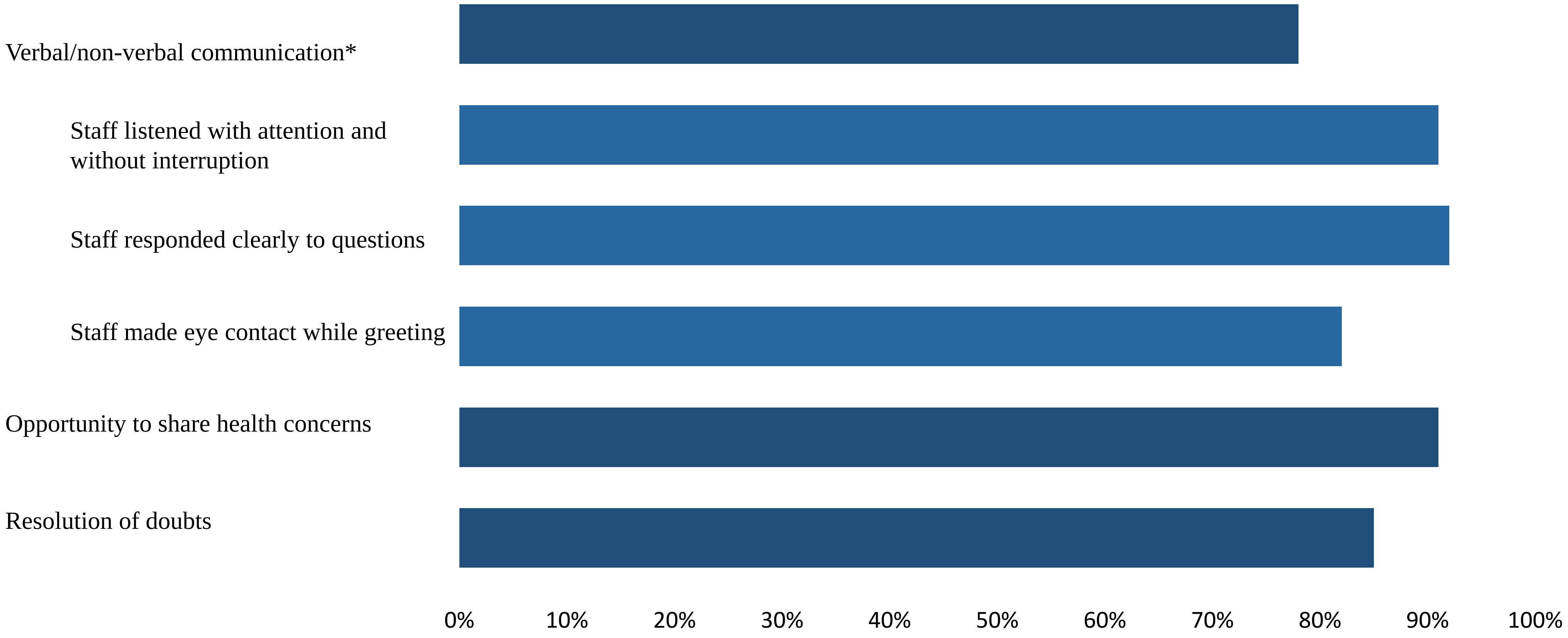

*Verbal/non-verbal communication is a composite variable comprised of those who reported positively to all three of the listed variables.

Figure 3. Patient global rating of treatment received (N=25,745)

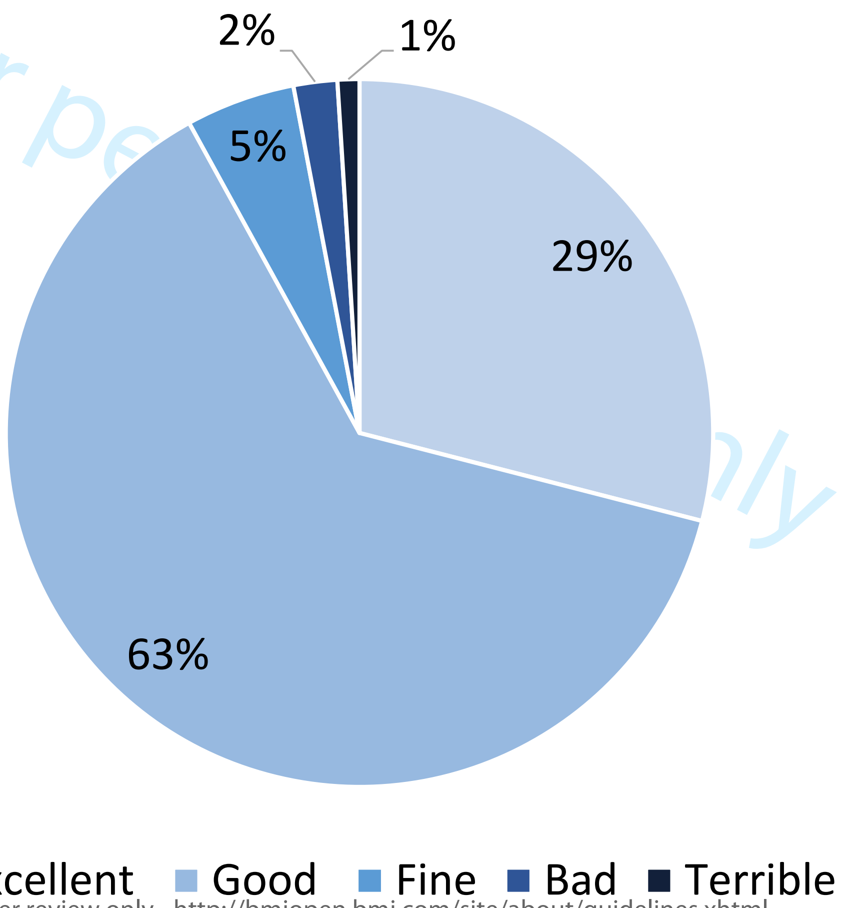

For peer review only - http://bmjopen.bmj.com/site/about/guidelines.xhtml

Holt et al.

STROBE Statement—Checklist of items that should be included in reports of ***cross-sectional studies***

	Item No	Recommendation	Page No
**Title and abstract**	1	(*a*) Indicate the study's design with a commonly used term in the title or the abstract	2
		(*b*) Provide in the abstract an informative and balanced summary of what was done and what was found	2
**Introduction**			
Background/rationale	2	Explain the scientific background and rationale for the investigation being reported	3-4
Objectives	3	State specific objectives, including any prespecified hypotheses	4
**Methods**			
Study design	4	Present key elements of study design early in the paper	4
Setting	5	Describe the setting, locations, and relevant dates, including periods of recruitment, exposure, follow-up, and data collection	4
Participants	6	(*a*) Give the eligibility criteria, and the sources and methods of selection of participants	4
Variables	7	Clearly define all outcomes, exposures, predictors, potential confounders, and effect modifiers. Give diagnostic criteria, if applicable	4-5
Data sources/ measurement	8*	For each variable of interest, give sources of data and details of methods of assessment (measurement). Describe comparability of assessment methods if there is more than one group	4-5
Bias	9	Describe any efforts to address potential sources of bias	5-6
Study size	10	Explain how the study size was arrived at	4
Quantitative variables	11	Explain how quantitative variables were handled in the analyses. If applicable, describe which groupings were chosen and why	5-6
Statistical methods	12	(*a*) Describe all statistical methods, including those used to control for confounding	6
		(*b*) Describe any methods used to examine subgroups and interactions	N/A
		(*c*) Explain how missing data were addressed	6
		(*d*) If applicable, describe analytical methods taking account of sampling strategy	6
		(*e*) Describe any sensitivity analyses	N/A
**Results**			
Participants	13*	(a) Report numbers of individuals at each stage of study—eg numbers potentially eligible, examined for eligibility, confirmed eligible, included in the study, completing follow-up, and analysed	6
		(b) Give reasons for non-participation at each stage	6
		(c) Consider use of a flow diagram	N/A
Descriptive data	14*	(a) Give characteristics of study participants (eg demographic, clinical, social) and information on exposures and potential confounders	6
		(b) Indicate number of participants with missing data for each variable of interest	N/A
Outcome data	15*	Report numbers of outcome events or summary measures	6
Main results	16	(*a*) Give unadjusted estimates and, if applicable, confounder-adjusted estimates and their precision (eg, 95% confidence interval). Make clear which confounders were adjusted for and why they were included	7-8

		(*b*) Report category boundaries when continuous variables were categorized	13
		(*c*) If relevant, consider translating estimates of relative risk into absolute risk for a meaningful time period	N/A
Other analyses	17	Report other analyses done—eg analyses of subgroups and interactions, and sensitivity analyses	N/A
**Discussion**			
Key results	18	Summarise key results with reference to study objectives	8
Limitations	19	Discuss limitations of the study, taking into account sources of potential bias or imprecision. Discuss both direction and magnitude of any potential bias	9
Interpretation	20	Give a cautious overall interpretation of results considering objectives, limitations, multiplicity of analyses, results from similar studies, and other relevant evidence	8-9
Generalisability	21	Discuss the generalisability (external validity) of the study results	10
**Other information**			
Funding	22	Give the source of funding and the role of the funders for the present study and, if applicable, for the original study on which the present article is based	N/A

*Give information separately for exposed and unexposed groups.

Note: An Explanation and Elaboration article discusses each checklist item and gives methodological background and published examples of transparent reporting. The STROBE checklist is best used in conjunction with this article (freely available on the Web sites of PLoS Medicine at http://www.plosmedicine.org/, Annals of Internal Medicine at http://www.annals.org/, and Epidemiology at http://www.epidem.com/). Information on the STROBE Initiative is available at www.strobe-statement.org.

BMJ Open

Factors associated with positive user experience with primary healthcare providers in Mexico: A multi-level modeling approach using national cross-sectional data

Journal:	*BMJ Open*
Manuscript ID	bmjopen-2019-029818.R1
Article Type:	Original research
Date Submitted by the Author:	02-Aug-2019
Complete List of Authors:	Holt, Kelsey; University of California, San Francisco,<br>Doubova, Svetlana; Mexican Institute of Social Security, Epidemiology and Health Services Research Unit, CMN Siglo XXI<br>Lee, Dennis; Harvard T. H. Chan School of Public Health, Global Health and Population<br>Perez-Cuevas, Ricardo; Secretaria de Salud, Coordinación de Asesores; Hospital Infantil de Mexico Federico Gomez, Centro de Estudios Economicos y Sociales en Salud<br>Leslie, Hannah; Harvard T. H. Chan School of Public Health, Global Health and Population
**Primary Subject Heading**:	Health services research
Secondary Subject Heading:	Patient-centred medicine
Keywords:	PRIMARY CARE, Quality in health care < HEALTH SERVICES ADMINISTRATION & MANAGEMENT, PUBLIC HEALTH

SCHOLARONE™
Manuscripts

Factors associated with positive user experience with primary healthcare providers in Mexico: A multi-level modeling approach using national cross-sectional data

Kelsey Holt, ScD, University of California, San Francisco; San Francisco, United States

Svetlana V. Doubova, MD, DSc, Mexican Institute of Social Security; Mexico City, Mexico

Dennis Lee, Harvard T.H. Chan School of Public Health; Boston, Massachusetts

Ricardo Perez Cuevas, DSc , Institute of Public Health in Mexico; Morelos, Mexico

Hannah H. Leslie, PhD, Harvard T.H. Chan School of Public Health; Boston, Massachusetts

Corresponding author: Kelsey Holt, 1001 Potrero Ave, San Francisco, CA, 94110; Kelsey.holt@ucsf.edu; 415-206-4234; fax: N/A

Key words: primary care, patient centered care, Mexico

Word count: 3,617

For peer review only

For peer review only - http://bmjopen.bmj.com/site/about/guidelines.xhtml

ABSTRACT

Objective: To investigate factors associated with patient experience with primary care in a large public health system in Mexico.

Methods: We analyzed a cross-sectional 2016 national satisfaction survey conducted by the Mexican Social Security Institute with beneficiaries over age 18. Patient-level data were merged with facility-level data and information on poverty by state. We defined patient experience measures as communication, opportunity to share health concerns, resolution of doubts, and overall treatment, and assessed the relationship of patient, facility, and state factors with patient experience using random effects logistic regression.

Results: 25,745 patients' responses from 319 primary care facilities were analyzed. The majority had positive experiences, including good communication (78%), the opportunity to share health concerns (91%), and resolution of doubts about treatment (85%). 29% of visits were rated as excellent overall. Differences between facilities and states accounted for 6.5-11.9% of the variation in patient experience for the four measures assessed. In adjusted models, larger patient population was correlated with worse reported communication, less opportunity to share concerns, and less resolution of doubts. Males reported more positive communication than females; older individuals reported more positive communication and experiences overall, but less opportunity to share concerns; and more highly educated individuals were less likely to report positive communication but more likely to report resolution of doubts and overall positive experiences. Preventive care visits were rated higher than curative visits in terms of resolution of doubts, but lower in terms of opportunity to share concerns.

Conclusion: Primary care quality improvement might bolster individual experiences with care, given that up to 12% of the variation in experience was attributable to facility and state level differences. The relationship between individual characteristics and experience ratings reinforces the importance of patients' expectations of care and the potential for differential treatment by providers to impact experience.

Strengths and limitations of this study

- This is one of very few studies to examine factors associated with positive user experience with primary care in a representative sample from a middle-income country.
- This study uses multi-level modeling techniques to examine the variance in user experience attributable to factors at the individual versus facility or state levels.
- Data are cross-sectional and thus we are unable to draw causal inferences from the observed associations.

For peer review only

- Findings are not generalizable to other sectors of the Mexican healthcare system, outside of the Mexican Institute of Social Security (IMSS).

For peer review only

For peer review only - http://bmjopen.bmj.com/site/about/guidelines.xhtml

INTRODUCTION

High quality primary care is essential to countries' ability to achieve Universal Health Coverage by 2030, one of the United Nations Sustainable Development Goals (SDGs) established in 2015.[1, 2] The core values of primary care, including person-centeredness and health equity, are in line with the SDGs' orientation towards reducing inequities and achieving good health and well-being for all:[1, 3] primary care provides a platform for delivering care that meets the entire populations' needs.[2]

In order to ensure that primary care is high quality and able to achieve the promise of promoting health for all, monitoring patient experiences is critical. Positive user experience is a core component of high quality health care and contributes to the population's confidence in the health system and achieving better health outcomes.[4] For instance, positive user experiences with primary care have been found to be associated with public perception of high healthcare quality,[5] self-rated health,[6] and high patient satisfaction.[7] Reviews of patient experiences in both primary and secondary care have found associations with clinical effectiveness and patient safety [8] and patient satisfaction.[9]

User experiences are the products of different patient and health services-related factors. Research examining the impact of health services-related factors on patient experience of care must account for the hierarchical nature of data: patients nested within providers, nested within institutions, nested within geographies.[10] Some studies, primarily from high-income countries, have examined the association between factors at different levels and patient experiences. At the individual level, age, education, and health status are the most common characteristics associated with patient experiences, with older and healthier individuals, and those with less education, tending to report more positive experiences.[11] Further, expectations for care play a clear role in overall satisfaction with care such that low expectations are associated with higher satisfaction.[9] Patient experience measures are increasingly recognized as valuable for quality improvement purposes as they are known to discriminate more effectively between practices than patient satisfaction measures.[4, 12] However, low expectations likely still influence to some degree the ways in which individuals report experiencing care.

At the health services level, variables such as presence of specific types of providers (e.g., nurses, in addition to physicians), health facility structure (e.g., fewer number of patients per provider, fewer numbers of physicians per practice, comprehensiveness of services provided by physicians) and functioning (e.g., long weekly working hours, non-training versus training facilities, payment structure, and operational agreements with other health systems) have been found to be associated with positive user experience with primary care.[13-16]

Much of this research is based in high-income settings. Similar research in lower-resource settings would provide crucial information to guide decision-makers in how to allocate resources to address deficiencies in system responsiveness to patients, and to the public to empower them to demand more patient-centered care. Further, in order to understand the degree to which experience measures are reliable indicators of the performance of healthcare systems (rather than a more limited indicator of variability in provider-patient relationships by individual-level socio-

For peer review only - http://bmjopen.bmj.com/site/about/guidelines.xhtml

demographic factors), it is critical to conduct analyses on the variability of patient experience attributable to health services factors.

Consistent with global initiatives, Mexico has a strong national commitment to both primary care and quality improvement to ensure the promises of universal access are achieved.[17, 18] Since 2009, user satisfaction/experience surveys are regular practice for public health providers in México. The Mexican Institute of Social Security (IMSS) is the largest provider, covering more than 63 million formal labor market employees and their families.[19] IMSS conducts annual National Satisfaction Surveys; yet, their results have not been analyzed comprehensively.

In this paper, we take advantage of this robust data collection effort by IMSS to investigate patient, facility, and geographic factors influencing experience with health care in the IMSS primary care system, and the degree to which variability in patient experience is due to patient-level versus facility-level factors.

METHODS

Data sources

Our primary data set comes from the November 2016 IMSS National Satisfaction Survey (ENSAT). IMSS regularly conducts these cross-sectional surveys with its beneficiaries over 18 years old at primary, secondary, and tertiary care services. This analysis focuses on patients at the primary care level. The survey's sampling design consists of a two-stage stratified probabilistic sampling where the probability of selecting a health facility is defined by region and facility size. The number of patients to be interviewed in each facility is estimated according to the average number of daily consultations with physicians and the proportion of patients satisfied in the previous survey. Patient recruitment is done through systematic sampling, with a random starting point and a fixed interval based on the estimated sample size and the average number of daily consultations in the clinic. A private firm (Berumen and Associates) conducted the survey. Trained interviewers carried out direct, structured interviews with IMSS health services users. The interviewers used a satisfaction questionnaire that the Center for Evaluation Research and Surveys of the National Institute of Public Health had previously validated. The response rate in the survey was 87%.

To perform the analysis, we merged 2016 ENSAT database with a facility-level database of a 2016 physical inventory of health care facilities and a 2016 record of patients affiliated with IMSS in each facility. Finally, a 2014 database of the National Council for the Evaluation of Social Development Policy (Spanish acronym CONEVAL) on the percentage of the population in each state living in poverty was merged into patient-level data.[20] Poverty is calculated by CONEVAL as a multidimensional measure based on criteria related to housing, household services, education, health care, social security, food, and per capita income.

Patient experience outcomes

We examined four binary dependent variables. The first is a composite measure of verbal and non-verbal communication, designated as positive if the patient answered favorably to three binary questions (translated from Spanish): "Did the staff who attended to you make eye contact while greeting you?"; "Did the staff who attended to you listen to you with attention and without

For peer review only - http://bmjopen.bmj.com/site/about/guidelines.xhtml

interruptions?"; and "Did the staff who attended to you respond clearly to your questions?" We also examined the questions: "Did you have the opportunity to tell the doctor or nurse what was bothering you about your health?" (referred to hereafter as "opportunity to share health concerns"); "Did you end up with doubts about the treatments necessary for your condition?" (reverse coded and referred to hereafter as "resolution of doubts"); and "How was the treatment you received in this facility during today's visit?", scored on a scale of 1-5 with 1 being "excellent," and 5 being "Terrible," collapsed to Excellent versus all other responses and referred to hereafter as the global measure.

Explanatory variables

In identifying independent variables to include in our models, we conceptualized patient experiences with IMSS healthcare delivery as being potentially influenced by factors at multiple levels (Figure 1). Available individual-level variables in the IMSS dataset included age (categorized into quartiles); sex (binary); and education level (high school or higher, less than high school, and less than primary school). We hypothesized that patients' age, sex, and educational status will explain some variance in reported experience due to differential treatment by providers and different expectations for care based on these factors. We also hypothesized experiences would vary by the primary reason for the visit, independent of patient characteristics, given that the nature of what is covered in a visit likely influences how providers approach patients (for example, primary care doctors are less comfortable discussing stigmatized issues such as sexual health and therefore exhibit poorer communication skills) and how well the visit can meet the patient's needs (for example, if their issue has numerous treatment options they may have a better experience than for those issues where treatments are limited). We therefore included the variables of self-reported reason for visit, collapsed from 149 categories into 10 categories based on ICD-10 codes, and type of consultation (curative visit, preventive care visit at the PREVENIMSS module at each clinic,[21] or dental visit). PREVENIMSS is a Spanish acronym for "Integrated Preventive Care Program," an initiative that provides targeted preventive care services to individuals based on their age and gender.

[Figure 1]

At the facility level, we hypothesized that size of facility could impact processes of care and included two variables that served as proxies for size: total number of rooms for all types of services and the number of people affiliated with a primary care clinic. We also included variables related to the presence or absence of programs that are designed to improve quality of care: presence of a special diabetes program comprising multidisciplinary teams of health-professionals (DIABETIMSS); presence of nurses' rooms attached to family doctors' consultation rooms; and availability of a nutritionist. Finally, we hypothesized that experience would vary by the percentage of population in a state that is living in poverty, given that this is likely reflective of the infrastructure in the state—including healthcare infrastructure. We thus included poverty rate in the facility's state as an independent variable. Continuous variables (consultation rooms, number of affiliates, poverty rate) were rescaled to fall on a scale of minimum 0 to maximum 1 to facilitate comparison across explanatory variables.

Analysis

For peer review only - http://bmjopen.bmj.com/site/about/guidelines.xhtml

We constructed three-level random-intercept logistic regression models for each dependent variable, nesting patients within facilities within states. Intra-class correlation (ICC) coefficients were calculated at facility and state levels to determine the amount of the total variance in the outcome due to differences at each level. We then added independent variables at patient and facility levels as fixed effects, retaining all variables in the final model. Random intercept models provide inference on the within-cluster difference associated with each covariate. For facility-level variables, in order to interpret these effects as differences between facilities, we present population-average coefficients odds ratio, the average odds ratio comparing subjects from different clusters that have identical observed values for other covariates, conditional on state for facility-level variables. We also examined interval odds ratios (IOR) at facility and state levels to compare the magnitude of each higher-level covariate to the random effect. For instance, a facility IOR provides the range of odds ratios comparing individuals with a one unit difference on the facility or state covariate from the 10th percentile to the 90th percentile of the random effect.[22, 23] (See Appendix 1 for all formulas). Stata version 15 was used for all analyses.

The IMSS dataset included weights to account for non-response. To account for missing data, we weighted complete cases based on inverse probability weights. Weights were calculated as the inverse of the probability of being a complete case predicted from regressing a binary indicator of completeness on patient-level covariates with no missing values (all except reason for visit and education level). State-level weights were set to 1; patient weights were rescaled around the mean weight at each facility. [24]

Ethical review

This secondary analysis of de-identified data was approved by IMSS Research and Ethics Committee (No. R 2018-785-037).

Patient and public involvement

Patients nor the public were involved in the design, conduct, or analysis of this study.

RESULTS

A total of 25,745 patients´ responses from 319 primary care facilities were analyzed. 1,593 cases were dropped for missing data. The median age of respondents was 49 years, 71% were female, and 70% had less than a high school education (Table 1). A small minority of the visits represented preventive (8%) or dental (2%) care, versus curative (90%). The most common reasons for the visit were diseases of the circulatory system (20%) or endocrine, nutritional, and metabolic diseases (19%). At the facility level, the average number of rooms in clinics was 20 (range=1-57), and the median number of enrolled patients was 83,691 (range=2,356-468,618). A minority had nurses rooms attached (6%), the DIABETIMSS program (19%), or nutritionists (39%). Participants lived in states where 41% of the population, on average, lived in poverty.

[Table 1]

The majority of patients reported the staff greeted them by looking them in their eye (82%), responded clearly to their questions (92%), and listened to them with attention and without

For peer review only - http://bmjopen.bmj.com/site/about/guidelines.xhtml

interruptions (91%) (Figure 2). 78% reported experiencing all three of these indicators of good verbal or non-verbal communication. 91% reported having the opportunity to share health concerns; 85% reported ending up with no doubts about their treatment. In 29% of visits they reported excellent care overall (Figure 3).

[Figure 2]

[Figure 3]

Examining ICC values, the amount of individual variation in propensity to report positive experiences that was due to systematic differences between facilities and states rather than systematic differences between patients ranged from 11.9% for the global measure to 6.5% for verbal/non-verbal communication (Table 2); approximately half of this variation was due to between-facility differences for all outcomes. These figures did not change more than a percentage point after adjustment for individual and facility characteristics, with the exception of opportunity to share health concerns, which decreased from 8.4% to 6.3% of residual variation due to systematic differences between facilities and states rather than between patients (data not shown).

[Table 2]

In the multivariable models predicting positive patient experiences with all patient- and facility-level covariates included, individuals within the same facility had higher odds of reporting positive communication if they were male (AOR 1.18, 95% CI 1.11, 1.26), 49-62 or 63-99 years compared to 18-33 years of age (AOR 1.16 and 1.30, 95% CI 1.01, 1.32 and 1.12, 1.50; respectively), , attended a visit related to pregnancy, childbirth, or the post-partum period (AOR 1.30, 95% CI 1.02, 1.67), or were seeking dental services (AOR 1.37, 95% CI 1.05, 1.79) after adjusting for other individual, facility, and state characteristics. More educated patients (with high school education or higher compared to less than primary school) within the same facility had lower odds of reporting high-quality communication, controlling for other covariates (AOR 0.84, 95% CI 0.76, 0.94).

The pattern for other outcomes was slightly different. Individuals within the same facility had lower odds of reporting they were given the opportunity to share health concerns if they were 34-48, 49-62, and 63-99 years compared to 18-33 years (AOR 0.83 and 0.83 and 0.82, 95% CI 0.72, 0.95 and 0.72, 0.97 and 0.70, 0.96; respectively); being seen for visits categorized in ICD-10 codes as injury, poisoning and other consequences of external causes (AOR 0.62, 95% CI 0.50, 0.77) or "factors influencing health status or contact with health services" (0.38, 95% CI 0.30, 0.49); or if they were being seen in a preventive care versus curative visit (AOR 0.27, 95% CI 0.24, 0.30). Individuals within the same health facility had lower odds of reporting resolution of doubts if they were being seen for symptoms, signs and abnormal clinical and laboratory findings, not elsewhere classified (AOR 0.58, 95% CI 0.48, 0.72), being seen for injury, poisoning and other consequences of external causes (AOR 0.70, 95% CI 0.52, 0.95), being seen for diseases of the musculoskeletal system and connective tissue (AOR 0.74, 95% CI 0.57, 0.97), or being seen for other reason (AOR 0.69, 95% CI 0.57, 0.83). Individuals within the same facility had higher odds of resolution of doubts if they had high school education or higher compared to less than primary school (AOR 1.18, 95% CI 1.05, 1.34) or were being seen for a preventive versus curative visit (AOR 1.26, 95% CI 1.05, 1.50), controlling for other covariates.

For peer review only - http://bmjopen.bmj.com/site/about/guidelines.xhtml

Finally, individuals within the same facility had higher odds of reporting overall excellent care if they were 49-62 and 63-99 years compared to 18-33 years (AOR 1.23 and 1.22, 95% CI 1.10, 1.38 and 1.07, 1.39; respectively) or if they had a primary school or high school education compared to less than primary school (AOR 1.13 and 1.35, 95% CI 1.01, 1.26 and 1.16, 1.57; respectively).

Across all facilities, larger patient population size affiliated with clinic was associated with lower odds of positive communication, opportunity to share concerns, and resolution of doubts in the adjusted models (AORs comparing the smallest population size to the largest 0.32 and 0.43 and 0.48; 95% CI 0.13, 0.74 and 0.21, 0.90 and 0.25, 0.93; respectively). Every facility-level IOR included the null, suggesting no evidence that every measured facility-level characteristic plays a more significant role in explaining variability than the random effect, conditional on state. However, the IOR for state poverty excluded the null for the fully adjusted models for positive communication (IOR 0.17 to 0.56) and opportunity to share health concerns (IOR 0.24, 0.77), suggesting that poverty plays a more important role than the state random effect in shaping these outcomes and that higher poverty levels are associated with less positive reports of patient experience.

DISCUSSION

This novel analysis of patient experiences with primary care in Mexico identified generally positive perspectives on health care and found that up to 12% of variation in experience occurs at the facility or state level. This suggests that quality improvement at the facility or regional level has the potential to improve individual experiences with care, though the degree of variation we uncovered at these higher levels is lower than that identified in other studies. For example, a study in general practice in Canada found that 20% of variance in perception of accessibility was attributable to practice-level characteristics[15], and a patient survey among primary care patients in California, United States, found that 28-48% was due to system related factors.[25]

While smaller patient population size was clearly associated with better patient experience, much of the facility-level variation could not be explained by measured covariates, suggesting further research is necessary in Mexico to identify levers for improvement. In the QUALICOPC study, conducted in 34—mainly European—countries, physicians providing a broader range of services and being payed through capitation had patients that reported more positive experiences, and more robust national primary care structure and higher health spending were also factors associated with better experience [16]. In-depth exploration of high-performing facilities in Mexico would help provide insights into these and other characteristics of facilities and the broader health system that may influence patient experience. Individual characteristics shaped experience ratings, reinforcing the importance of patients' expectations of care and the potential for differential treatment by providers to impact experience. Systematic assessment of patient expectations and provider bias may help to inform health system improvements.

The finding that larger patient population affiliated with clinic was correlated with worse reported patient experience on a number of dimensions is consistent with findings from other research [14] and suggests that particular attention may need to be paid to improving patient experience in settings with high patient volume.

For peer review only - http://bmjopen.bmj.com/site/about/guidelines.xhtml

The pattern of relationships between individual-level characteristics and patient experience was variable across outcomes. Males reported more positive communication than females; this may reflect a difference in how providers communicate with males or different expectations for care.[26] Older individuals tended to report more positive experiences overall and specifically with communication, consistent with a review of the impact of individual characteristics on experiences.[11] Older individuals were less likely than those between 18-33 years to report opportunity to share their health concerns, again either representing a difference in communication style on the part of providers or different expectations for care by patients by age. In terms of education, more educated individuals were less likely to report positive communication, consistent with other research and likely due to higher expectations for care.[11] However, they were more likely to report resolution of doubts and positive overall experiences. This could represent the fact that those with the most education are empowered to be more "active" participants in their care,[27] or possible provider discrimination against patients with less than primary school.

The reason for a patient's visit was consistently related to their experience, with preventive care visits eliciting higher ratings for resolution of doubts (though lower ratings for opportunity to share concerns), and dental visits being associated with better communication. This may reflect the different nature of preventive care, wherein ability to resolve patients' doubts is more achievable than when patients are being seen for a particular medical concern. It could, however, reflect better quality care for patients in the preventive care or dental modules. Specific conditions were also associated with report of better experiences in some cases, also likely reflecting either the nature of the visit or systematic differences in the ways providers treat patients who are being seen for different conditions.

These findings should be considered in light of several limitations. First, the data are cross-sectional and we are unable to draw causal inferences from the observed associations between independent and dependent variables. Further, though we were able to identify the overall amount of variance attributable to the facility level, we had few potential explanatory factors available at the facility level. Second, available datasets did not indicate which providers patients had seen, keeping us from being able to parse the variance in patient experience attributable to differences between providers. Future efforts to collect national experience data in the IMSS system could benefit from collecting these data. Third, we did not have access to information on all IMSS primary care visits to be able to compare the demographics of our sample with the overall population of users. However, the response rate was high (87%), suggesting adequate representativeness. Finally, the results are generalizable only to users of the IMSS system, as the survey did not include patients from other health sectors in Mexico.

Our findings shed light on the influences at individual, facility, and state levels on patients' experiences with a large public health system in Mexico. These findings can inform efforts to improve patient experiences, thereby promoting confidence in the health system and better health outcomes. Key priorities for future work include further investigation of the facility-level factors accounting for differences in patient experience, as well as more robust monitoring of patients' expectations for care and their potential experiences with bias to better understand what accounts for individual level differences in experience.

For peer review only - http://bmjopen.bmj.com/site/about/guidelines.xhtml

For peer review only

For peer review only - http://bmjopen.bmj.com/site/about/guidelines.xhtml

Table 1. Patient and facility characteristics

	n (%)
**Patient characteristics (N=25,745)**	
Sex	
Male	7,533 (29.3)
Female	18,212 (70.7)
Age	
Median, Range (Interquartile range)	49, 18-99 (34-63)
Level of education	
Less than primary school	4,006 (15.6)
Less than high school	13,914 (54.0)
High school or more	7,825 (30.4)
Visit type	
Curative	23,144 (89.9)
Preventive	2,024 (7.9)
Dental	577 (2.2)
Reason for visit	
Diseases of the circulatory system	5,132 (19.9)
Endocrine, nutritional and metabolic diseases	4,898 (19.0)
Other	3,085 (12.0)
Diseases of the respiratory system	2,386 (9.3)
Factors influencing health status and contact with health services	1,805 (7.0)
Pregnancy, childbirth and the puerperium	1,781 (6.9)
Diseases of the musculoskeletal system and connective tissue	1,710 (6.6)
Diseases of the digestive system	1,691 (6.6)
Symptoms, signs and abnormal clinical and laboratory findings, not elsewhere classified	1,664 (6.5)
Injury, poisoning and certain other consequences of external causes	1,593 (6.2)
Percent poverty in state	
Mean (Range), SD	41.1 (20.4-76.2), 12.9
**Facility characteristics (N=319)**	
Number of consultation rooms	
Mean (Range), SD	20.4 (1-57), 13.0
Diabetes program	
No diabetes program	258 (80.9)
Has diabetes program	61 (19.1)
Nutritionist	
No nutritionist	194 (60.8)
At least one nutritionist	125 (39.2)
Nurses room	

For peer review only - http://bmjopen.bmj.com/site/about/guidelines.xhtml

No nurse room	300 (94.0)
At least one nurse room	19 (6.0)
Patient population size	
Median (Range), SD	83,691 (2,356-468,618), 86,231.0

For peer review only

Table 2. Multi-level logistic regression results: odds ratios with 95% confidence intervals (n=25,745 patients, N= 319 facilities)

	Positive verbal/non-verbal communication		Opportunity to share health concerns		Resolution of doubts		Excellent global experience rating	
**Individual Level Variables**	AOR (95% CI)	p-value	AOR (95% CI)	p-value	AOR (95% CI)	p-value	AOR (95% CI)	p-value
Male sex	**1.18 (1.11, 1.26)**	**<.0001**	1.06 (.94, 1.21)	.328	.98 (.87, 1.12)	.799	1.07 (.98, 1.17)	.130
Age								
18-33 years	Ref		Ref		Ref		Ref	
34-48 years	1.08 (.94, 1.23)	.260	**.83 (.72, .95)**	**.008**	.90 (.79, 1.02)	.098	1.04 (.93, 1.16)	.517
49-62 years	**1.16 (1.01, 1.32)**	**.033**	**.83 (.72, .97)**	**.018**	.95 (.81, 1.11)	.490	**1.23 (1.10, 1.38)**	**.0006**
63-99 years	**1.30 (1.12, 1.50)**	**.009**	**.82 (.70, 0.96)**	**.016**	.98 (.85, 1.14)	.836	**1.22 (1.07, 1.39)**	**.003**
Level of education								
Less than primary school	Ref		Ref		Ref		Ref	
Less than high school	.90 (.80, 1.02)	.097	1.01 (.81, 1.25)	.934	1.07 (.96, 1.19)	.223	**1.13 (1.01, 1.26)**	**.030**
High school or more	**.84 (.76, .94)**	**.003**	1.14 (.92, 1.41)	.235	**1.18 (1.05, 1.34)**	**.008**	**1.35 (1.16, 1.57)**	**.0003**
Visit type								
Curative	Ref		Ref		Ref		Ref	
Preventive	1.02 (.85, 1.22)	.830	**.27 (.24, .30)**	**<.0001**	**1.26 (1.05, 1.50)**	**.015**	1.07 (.88, 1.30)	.462
Dental	**1.37 (1.05, 1.79)**	**.021**	1.09 (.73, 1.64)	.671	1.22 (.89, 1.68)	.207	1.21 (.91, 1.62)	.189
Reason for visit								
Diseases of the circulatory system	Ref		Ref		Ref		Ref	
Endocrine, nutritional and metabolic diseases	1.02 (.88, 1.18)	.778	1.01 (.82, 1.24)	.919	.88 (.73, 1.06)	.181	.95 (.83, 1.09)	.491
Other	.89 (.75, 1.04)	.138	.87 (.72, 1.05)	.140	**.69 (.57, .83)**	**.0003**	.95 (.81, 1.12)	.553
Diseases of the respiratory system	.94 (.82, 1.08)	.361	1.16 (.84, 1.61)	.351	.96 (.76, 1.23)	.751	.95 (.82, 1.09)	.417
Factors influencing health status and contact with health services	1.12 (.90, 1.38)	.303	**.38 (.30, .49)**	**<.0001**	1.03 (.82, 1.30)	.776	1.14 (.93, 1.39)	.197
Pregnancy, childbirth and	**1.30 (1.02, 1.67)**	**.037**	.93 (.73, 1.20)	.572	.89 (.71, 1.10)	.268	1.08 (.88, 1.34)	.449

For peer review only - http://bmjopen.bmj.com/site/about/guidelines.xhtml

the puerperium								
Diseases of the musculoskeletal system and connective tissue	.82 (.66, 1.02)	.077	.82 (.65, 1.03)	.085	**.74 (.57, 0.97)**	**.032**	1.01 (.89, 1.20)	.872
Diseases of the digestive system	.88 (.73, 1.06)	.163	.86 (.64, 1.14)	.283	.75 (.55, 1.03)	.072	.86 (.72, 1.03)	.107
Symptoms, signs and abnormal clinical and laboratory findings, not elsewhere classified	.89 (.71, 1.11)	.281	.88 (.67, 1.17)	.365	**.58 (.48, .72)**	**<.0001**	1.04 (.87, 1.25)	.660
Injury, poisoning and certain other consequences of external causes	.88 (.73, 1.06)	.162	**.62 (.50, .77)**	**<.0001**	**.70 (.52, .95)**	**.023**	.90 (.74, 1.09)	.256
**Facility Level Variables**	AOR (95% CI) or IOR	p-value	AOR (95% CI) or IOR	p-value	AOR (95% CI) or IOR	p-value	AOR (95% CI) or IOR	p-value
Number of consultation rooms								
AOR	1.65 (.88, 3.36)	.163	.93 (.37, 2.34)	.868	.84 (.41, 1.74)	.644	1.04 (.51, 2.10)	.915
IOR	0.57, 1.85		0.60, 1.95		0.52, 2.42		0.44, 2.24	
Presence of:								
Diabetes program								
AOR	1.02 (0.88, 1.19)	.746	1.08 (.81, 1.45)	.091	1.11 (.92, 1.36)	.269	0.99 (.82, 1.19)	.921
IOR	0.48, 1.57		0.54, 1.74		0.45, 2.11		0.41, 2.07	
Nutritionist								
AOR	1.00 (.84, 1.19)	.989	1.07 (.83, 1.40)	.583	0.95 (.73, 1.25)	.719	1.10 (.89, 1.35)	.365
IOR	0.63, 2.00		0.44, 1.51		0.48, 1.71		0.30, 1.75	
Nurses room								
AOR	.87 (.69, 1.10)	.232	.97 (.67, 1.40)	.859	.98 (.72, 1.33)	. 872	0.92 (.79, 1.08)	.296
IOR	0.55, 1.80		0.60, 1.93		0.44, 2.05		0.49, 2.48	
Patient population size								
AOR	**.32 (.13, .74)**	**.010**	**.43 (.21, 0.90)**	**.026**	**.48 (.25, 0.93)**	**.030**	1.19 (.48, 2.91)	.702

For peer review only - http://bmjopen.bmj.com/site/about/guidelines.xhtml

	IOR	0.92, 3.00		0.52, 1.66		0.39, 1.82		0.46, 2.34	
**State Level Variable**									
Percent poverty in state (minimum = 20%; versus maximum = 76%)									
	AOR	1.12 (.69, 1.82)	.641	.82 (.50, 1.35)	.416	.48 (.50, 1.67)	.757	.74 (.42, 1.30)	.285
	IOR	0.17, 0.56		0.24, 0.77		0.22, 1.02		0.53, 2.69	
**Intraclass correlation between facilities and states**		6.5%		8.3%		8.9%		11.9%	

For peer review only - http://bmjopen.bmj.com/site/about/guidelines.xhtml

Author statement: KH, SD, and HL conceived of the study; KH, HL, and DL conducted the analysis; KH drafted the manuscript; SD, HL, DL, and RPC provided substantive feedback on the manuscript.

Funding statement: HL and DL received support from the Bill and Melinda Gates Foundation (Award OPP1161450, Kruk)

Acknowledgements: The authors would like to acknowledge the generous help of Sarah Wulf in preparing the tables and figures for the manuscript.

Competing interests: We have no competing interests to declare.

Data sharing statement:
Patient and state level data are available in public, open access repositories
Patient level outcomes: http://datos.imss.gob.mx/
State level poverty data:
https://www.coneval.org.mx/coordinacion/entidades/Paginas/inicioent.aspx

Facility level data may be obtained from a third party and are not publicly available
Data on IMSS facility characteristics are available from IMSS upon request after approval for analyses from the IMSS Health Research Ethics Committee (contact: comiteeticainv.imss@gmail.com)

For peer review only

REFERENCES CITED

1. Pettigrew, L.M., et al., *Primary health care and the Sustainable Development Goals.* The Lancet, 2015. **386**(10009): p. 2119-2121.
2. Stigler, F.L., et al., *No universal health coverage without primary health care.* The Lancet, 2016. **387**(10030): p. 1811.
3. Van Lerberghe, W., *The world health report 2008: primary health care: now more than ever.* 2008: World Health Organization.
4. Kruk, M.E., et al., *High-quality health systems in the Sustainable Development Goals era: time for a revolution.* The Lancet Global Health, 2018.
5. Doubova, S.V., et al., *Attributes of patient-centered primary care associated with the public perception of good healthcare quality in Brazil, Colombia, Mexico and El Salvador.* Health policy and planning, 2016. **31**(7): p. 834-843.
6. Guanais, F., et al., *Patient-centered primary care and self-rated health in 6 Latin American and Caribbean countries: Analysis of a public opinion cross-sectional survey.* PLoS medicine, 2018. **15**(10): p. e1002673.
7. Birhanu, Z., et al., *Determinants of satisfaction with health care provider interactions at health centres in central Ethiopia: a cross sectional study.* BMC health services research, 2010. **10**(1): p. 78.
8. Doyle, C., L. Lennox, and D. Bell, *A systematic review of evidence on the links between patient experience and clinical safety and effectiveness.* BMJ open, 2013. **3**(1): p. e001570.
9. Batbaatar, E., et al., *Determinants of patient satisfaction: a systematic review.* Perspectives in public health, 2017. **137**(2): p. 89-101.
10. Hearld, L.R., et al., *How do hospital organizational structure and processes affect quality of care? A critical review of research methods.* Medical Care Research and Review, 2008. **65**(3): p. 259-299.
11. Sandager, M., M. Freil, and J.L. Knudsen, *Please tick the appropriate box: Perspectives on patient reported experience.* Patient Experience Journal, 2016. **3**(1): p. 63-79.
12. Salisbury, C., M. Wallace, and A.A. Montgomery, *Patients' experience and satisfaction in primary care: secondary analysis using multilevel modelling.* Bmj, 2010. **341**: p. c5004.
13. Cohidon, C., P. Wild, and N. Senn, *Patient experience in primary care: association with patient, physician and practice characteristics in a fee-for-service system.* Swiss medical weekly, 2018. **148**.
14. Heje, H.N., et al., *Doctor and practice characteristics associated with differences in patient evaluations of general practice.* BMC health services research, 2007. **7**(1): p. 46.
15. Haggerty, J.L., et al., *Practice features associated with patient-reported accessibility, continuity, and coordination of primary health care.* The Annals of Family Medicine, 2008. **6**(2): p. 116-123.
16. Schäfer, W.L.A., et al., *Are people's health care needs better met when primary care is strong? A synthesis of the results of the QUALICOPC study in 34 countries*. Primary Health Care Research & Development, 2019. 20(e104): 1–9. doi:10.1017/S1463423619000434
17. Atun, R., et al., *Health-system reform and universal health coverage in Latin America.* The Lancet, 2015. **385**(9974): p. 1230-1247.
18. Doubova, S.V., et al., *Quality governance in a pluralistic health system: Mexican experience and challenges.* The Lancet Global Health, 2018. **6**(11): p. e1149-e1152.
19. Gobierno de los Estados Unidos Mexicanos. Cuarto Informe de Gobierno 2015-2016. Anexo estadístico. Ciudad de México 2016. Available at: http://www.presidencia.gob.mx/cuartoinforme/
20. Consejo Nacional de Evaluación de la Política de Desarrollo Social. Información de pobreza y evaluación en las entidades federativas. Mexico: CONEVAL, 2014. Available at

For peer review only - http://bmjopen.bmj.com/site/about/guidelines.xhtml

http://www.coneval.org.mx/coordinacion/entidades/Paginas/inicioent.aspx
21. Gutiérrez, G., et al., *Strengthening preventive care programs: a permanent challenge for healthcare systems; lessons from PREVENIMSS México.* BMC public health, 2010. **10**(1): p. 417.
22. Austin, P.C. and J. Merlo, *Intermediate and advanced topics in multilevel logistic regression analysis.* Statistics in medicine, 2017. **36**(20): p. 3257-3277.
23. Larsen, K. and J. Merlo, *Appropriate assessment of neighborhood effects on individual health: integrating random and fixed effects in multilevel logistic regression.* American journal of epidemiology, 2005. **161**(1): p. 81-88.
24. Rabe-Hesketh, S. and A. Skrondal, Mul*tilevel modeling of complex survey data*. Journal of the Royal Statistical Society, 2006. Series A 169, 805-827.
25. Rodriguez, H.P., et al., *Attributing sources of variation in patients' experiences of ambulatory care.* Medical care, 2009: p. 835-841.
26. Roder-DeWan, S., et al., *Good ratings for poor quality: Low healthcare expectations among internet users in 12 low- and middle-income countries*. In Press
27. Leonard, K.L., *Active patients in rural African health care: implications for research and policy.* Health policy and planning, 2013. **29**(1): p. 85-95.

For peer review only

For peer review only - http://bmjopen.bmj.com/site/about/guidelines.xhtml

Figure 1. Conceptual model of relationship between model variables and reported experience with provider (N=25,745 patients and 319 facilities)

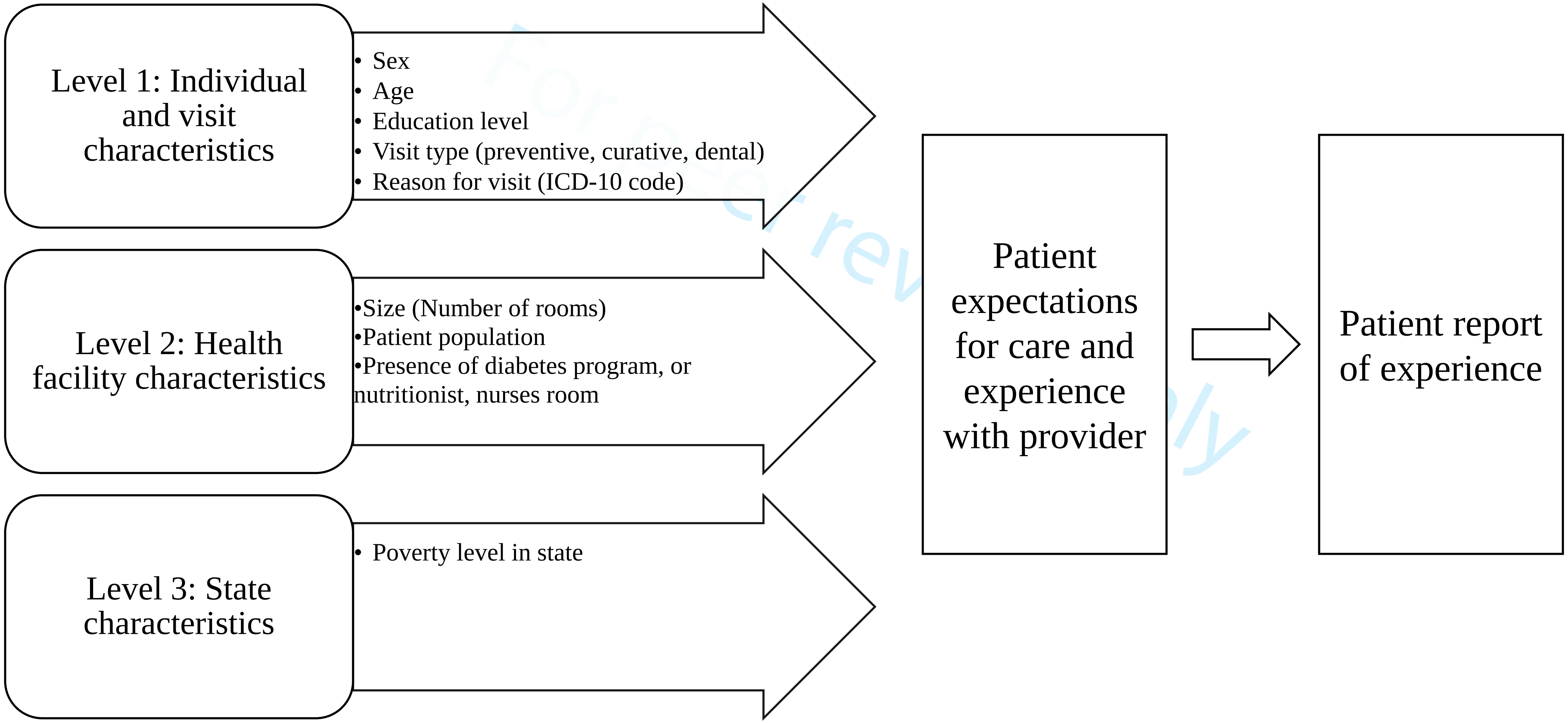

Figure 2. Patient report of experience with communicating with health care providers (N=25,745)

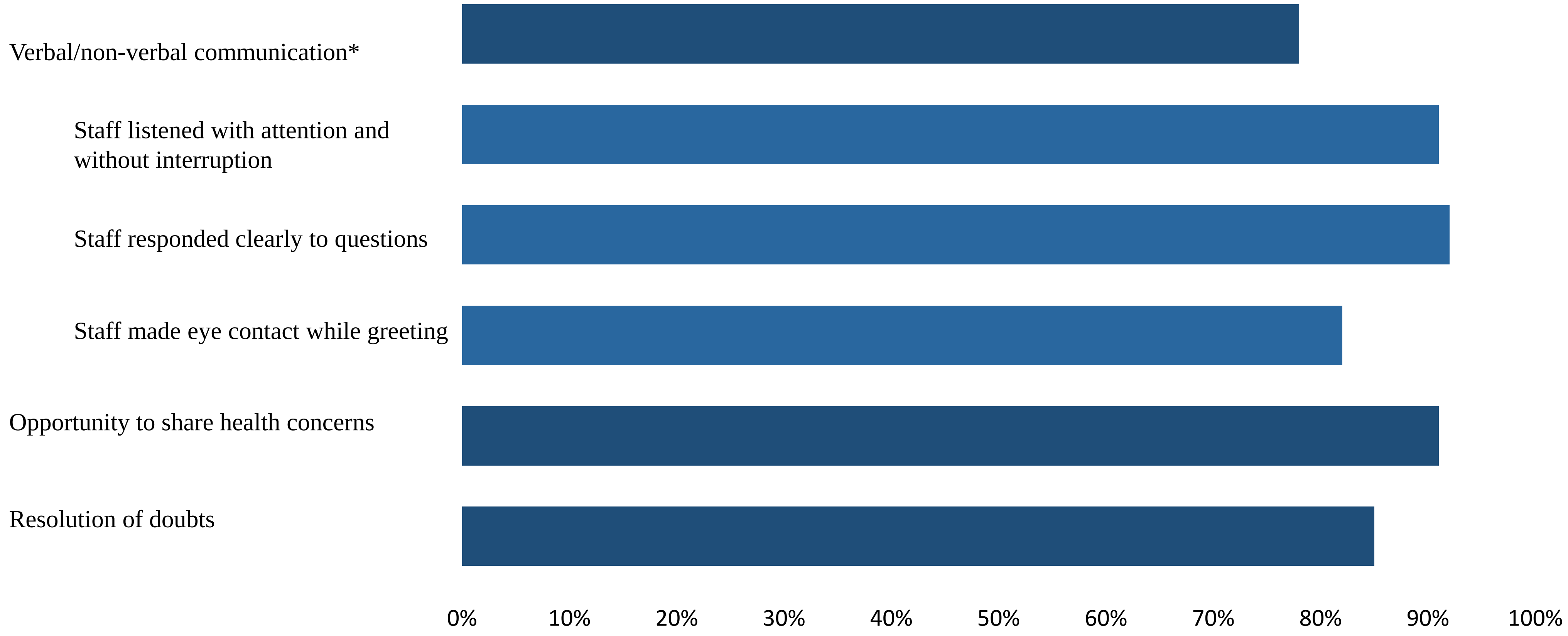

*Verbal/non-verbal communication is a composite variable comprised of those who reported positively to all three of the listed variables.

Figure 3. Patient global rating of treatment received (N=25,745)

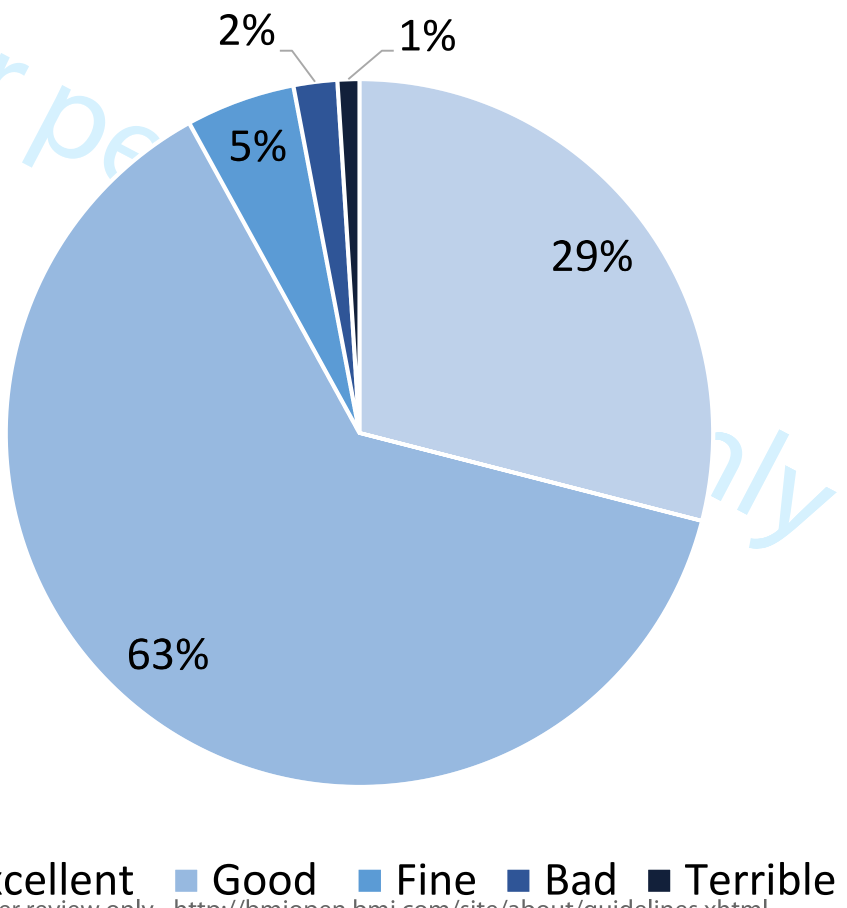

For peer review only - http://bmjopen.bmj.com/site/about/guidelines.xhtml

Appendix 1: Formulas

ICC:

Level 2:

$$\frac{\widehat{\tau_2^2} + \widehat{\tau_3^2}}{\widehat{\tau_2^2} + \widehat{\tau_3^2} + \frac{\pi^2}{3}}$$

Level 3:

$$\frac{\widehat{\tau_3^2}}{\widehat{\tau_2^2} + \widehat{\tau_3^2} + \frac{\pi^2}{3}}$$

MOR:

Level 2:

$$\exp\left(\sqrt{2\widehat{\tau_2^2}} * \Phi^{-1}(0.75)\right)$$

Level 3:

$$\exp\left(\sqrt{2\widehat{\tau_3^2}} * \Phi^{-1}(0.75)\right)$$

IOR:

Level 2:

$$\left[\exp\left(\widehat{\alpha_2} + \sqrt{2\widehat{\tau_2^2}} * \Phi^{-1}(0.1)\right), \exp\left(\widehat{\alpha_2} + \sqrt{2\widehat{\tau_2^2}} * \Phi^{-1}(0.9)\right)\right]$$

Level 3:

$$\left[\exp\left(\widehat{\alpha_3} + \sqrt{2\widehat{\tau_3^2}} * \Phi^{-1}(0.1)\right), \exp\left(\widehat{\alpha_3} + \sqrt{2\widehat{\tau_3^2}} * \Phi^{-1}(0.9)\right)\right]$$

Population Average Coefficients:

Level 2:

$$\widehat{\alpha_{PA,2}} = \frac{\widehat{\alpha_2}}{\sqrt{1 + \left(16^2 * \frac{3}{(15 * \pi)^2}\right) * \widehat{\tau_2^2}}}$$

For peer review only

Level 3:

$$\widehat{\alpha_{PA,3}} = \frac{\widehat{\alpha_3}}{\sqrt{1 + \left(16^2 * \frac{3}{(15 * \pi)^2}\right) * \widehat{\tau_3^2}}}$$

Where the subscript denotes the level of the variable, and level 2 variances (facility-level) are conditional on the third level (state-level), $\widehat{\tau^2}$ is the estimated group variance, and $\hat{\alpha}$ is an estimated regression coefficient. Φ represents the cumulative distribution function of the standard normal distribution.

For peer review only

Holt et al.

STROBE Statement—Checklist of items that should be included in reports of ***cross-sectional studies***

	Item No	Recommendation	Page No
**Title and abstract**	1	(*a*) Indicate the study's design with a commonly used term in the title or the abstract	2
		(*b*) Provide in the abstract an informative and balanced summary of what was done and what was found	2
**Introduction**			
Background/rationale	2	Explain the scientific background and rationale for the investigation being reported	3-4
Objectives	3	State specific objectives, including any prespecified hypotheses	4
**Methods**			
Study design	4	Present key elements of study design early in the paper	4
Setting	5	Describe the setting, locations, and relevant dates, including periods of recruitment, exposure, follow-up, and data collection	4
Participants	6	(*a*) Give the eligibility criteria, and the sources and methods of selection of participants	4
Variables	7	Clearly define all outcomes, exposures, predictors, potential confounders, and effect modifiers. Give diagnostic criteria, if applicable	4-5
Data sources/ measurement	8*	For each variable of interest, give sources of data and details of methods of assessment (measurement). Describe comparability of assessment methods if there is more than one group	4-5
Bias	9	Describe any efforts to address potential sources of bias	5-6
Study size	10	Explain how the study size was arrived at	4
Quantitative variables	11	Explain how quantitative variables were handled in the analyses. If applicable, describe which groupings were chosen and why	5-6
Statistical methods	12	(*a*) Describe all statistical methods, including those used to control for confounding	6
		(*b*) Describe any methods used to examine subgroups and interactions	N/A
		(*c*) Explain how missing data were addressed	6
		(*d*) If applicable, describe analytical methods taking account of sampling strategy	6
		(*e*) Describe any sensitivity analyses	N/A
**Results**			
Participants	13*	(a) Report numbers of individuals at each stage of study—eg numbers potentially eligible, examined for eligibility, confirmed eligible, included in the study, completing follow-up, and analysed	6
		(b) Give reasons for non-participation at each stage	6
		(c) Consider use of a flow diagram	N/A
Descriptive data	14*	(a) Give characteristics of study participants (eg demographic, clinical, social) and information on exposures and potential confounders	6
		(b) Indicate number of participants with missing data for each variable of interest	N/A
Outcome data	15*	Report numbers of outcome events or summary measures	6
Main results	16	(*a*) Give unadjusted estimates and, if applicable, confounder-adjusted estimates and their precision (eg, 95% confidence interval). Make clear which confounders were adjusted for and why they were included	7-8

		(*b*) Report category boundaries when continuous variables were categorized	13
		(*c*) If relevant, consider translating estimates of relative risk into absolute risk for a meaningful time period	N/A
Other analyses	17	Report other analyses done—eg analyses of subgroups and interactions, and sensitivity analyses	N/A
**Discussion**			
Key results	18	Summarise key results with reference to study objectives	8
Limitations	19	Discuss limitations of the study, taking into account sources of potential bias or imprecision. Discuss both direction and magnitude of any potential bias	9
Interpretation	20	Give a cautious overall interpretation of results considering objectives, limitations, multiplicity of analyses, results from similar studies, and other relevant evidence	8-9
Generalisability	21	Discuss the generalisability (external validity) of the study results	10
**Other information**			
Funding	22	Give the source of funding and the role of the funders for the present study and, if applicable, for the original study on which the present article is based	N/A

*Give information separately for exposed and unexposed groups.

Note: An Explanation and Elaboration article discusses each checklist item and gives methodological background and published examples of transparent reporting. The STROBE checklist is best used in conjunction with this article (freely available on the Web sites of PLoS Medicine at http://www.plosmedicine.org/, Annals of Internal Medicine at http://www.annals.org/, and Epidemiology at http://www.epidem.com/). Information on the STROBE Initiative is available at www.strobe-statement.org.

BMJ Open

Factors associated with positive user experience with primary healthcare providers in Mexico: A multi-level modeling approach using national cross-sectional data

Journal:	*BMJ Open*
Manuscript ID	bmjopen-2019-029818.R2
Article Type:	Original research
Date Submitted by the Author:	16-Dec-2019
Complete List of Authors:	Holt, Kelsey; University of California, San Francisco,<br>Doubova, Svetlana; Mexican Institute of Social Security, Epidemiology and Health Services Research Unit, CMN Siglo XXI<br>Lee, Dennis; Harvard T. H. Chan School of Public Health, Global Health and Population<br>Perez-Cuevas, Ricardo; Secretaria de Salud, Coordinación de Asesores; Hospital Infantil de Mexico Federico Gomez, Centro de Estudios Economicos y Sociales en Salud<br>Leslie, Hannah; Harvard T. H. Chan School of Public Health, Global Health and Population
**Primary Subject Heading**:	Health services research
Secondary Subject Heading:	Patient-centred medicine
Keywords:	PRIMARY CARE, Quality in health care < HEALTH SERVICES ADMINISTRATION & MANAGEMENT, PUBLIC HEALTH

SCHOLARONE™
Manuscripts

Factors associated with positive user experience with primary healthcare providers in Mexico: A multi-level modeling approach using national cross-sectional data

Kelsey Holt, ScD, University of California, San Francisco; San Francisco, United States

Svetlana V. Doubova, MD, DSc, Mexican Institute of Social Security; Mexico City, Mexico

Dennis Lee, Harvard T.H. Chan School of Public Health; Boston, Massachusetts

Ricardo Perez Cuevas, DSc , Institute of Public Health in Mexico; Morelos, Mexico

Hannah H. Leslie, PhD, Harvard T.H. Chan School of Public Health; Boston, Massachusetts

Corresponding author: Kelsey Holt, 1001 Potrero Ave, San Francisco, CA, 94110; Kelsey.holt@ucsf.edu; 415-206-4234; fax: N/A

Key words: primary care, patient centered care, Mexico

Word count: 4,037

For peer review only

ABSTRACT

Objective: To investigate factors associated with patient experience with primary care in a large public health system in Mexico and determine the amount of variability in experience attributable to facility- and state-level factors.

Methods: We analyzed cross-sectional 2016 national satisfaction survey data from the Mexican Social Security Institute. Patient-level data were merged with facility-level data and information on poverty by state. We assessed general contextual effects and examined the relationship of patient, facility, and state factors with four patient experience measures using random effects logistic regression.

Results: 25,745 patients' responses from 319 facilities were analyzed. The majority experienced good communication (78%), the opportunity to share health concerns (91%), and resolution of doubts (85%). 29% of visits were rated as excellent. Differences between facilities and states accounted for up to 12% and 6% of the variation in patient experience, respectively. Inclusion of facility-level contextual effects improved model predictions by 8-12%; models with facility random effects and individual covariates correctly predicted 64-71% of individual outcomes. In adjusted models, larger patient population was correlated with worse reported communication, less opportunity to share concerns, and less resolution of doubts. Males reported more positive communication; older individuals reported more positive communication and experiences overall, but less opportunity to share concerns; and more educated individuals were less likely to report positive communication but more likely to report resolution of doubts and overall positive experiences. Preventive care visits were rated higher than curative visits for resolution of doubts, but lower for opportunity to share concerns.

Conclusion: Quality improvement might bolster individual experiences with primary care, given that up to 12% of the variation in experience was attributable to facility-level differences. The relationship between individual characteristics and experience ratings reinforces the importance of patients' expectations of care and the potential for differential treatment by providers to impact experience.

Strengths and limitations of this study

- This is one of very few studies to examine factors associated with positive user experience with primary care in a representative sample from a middle-income country.
- This study uses multi-level modeling techniques to examine the variance in user experience attributable to factors at the individual versus facility or state levels.
- Data are cross-sectional and thus we are unable to draw causal inferences from the observed associations.

For peer review only - http://bmjopen.bmj.com/site/about/guidelines.xhtml

For peer review only

- Findings are not generalizable to other sectors of the Mexican healthcare system, outside of the Mexican Institute of Social Security (IMSS).

For peer review only

For peer review only - http://bmjopen.bmj.com/site/about/guidelines.xhtml

INTRODUCTION

High quality primary care is essential to countries' ability to achieve Universal Health Coverage by 2030, one of the United Nations Sustainable Development Goals (SDGs) established in 2015.[1, 2] The core values of primary care, including person-centeredness and health equity, are in line with the SDGs' orientation towards reducing inequities and achieving good health and well-being for all:[1, 3] primary care provides a platform for delivering care that meets the entire populations' needs.[2]

In order to ensure that primary care is high quality and able to achieve the promise of promoting health for all, monitoring patient experiences is critical. Positive user experience is a core component of high quality health care and contributes to the population's confidence in the health system and achieving better health outcomes.[4] For instance, positive user experiences with primary care have been found to be associated with public perception of high healthcare quality,[5] self-rated health,[6] and high patient satisfaction.[7] Reviews of patient experiences in both primary and secondary care have found associations with clinical effectiveness and patient safety [8] and patient satisfaction.[9]

User experiences are the products of different patient and health services-related factors. Research examining the impact of health services-related factors on patient experience of care must account for the hierarchical nature of data: patients nested within providers, nested within institutions, nested within geographies.[10] Some studies, primarily from high-income countries, have examined the association between factors at different levels and patient experiences. At the individual level, age, education, and health status are the most common characteristics associated with patient experiences, with older and healthier individuals, and those with less education, tending to report more positive experiences.[11] Further, expectations for care play a clear role in overall satisfaction with care such that low expectations are associated with higher satisfaction.[9] Patient experience measures are increasingly recognized as valuable for quality improvement purposes as they are known to discriminate more effectively between practices than patient satisfaction measures.[4, 12] However, low expectations likely still influence to some degree the ways in which individuals report experiencing care.

At the health services level, variables such as presence of specific types of providers (e.g., nurses, in addition to physicians), health facility structure (e.g., fewer number of patients per provider, fewer numbers of physicians per practice, comprehensiveness of services provided by physicians) and functioning (e.g., long weekly working hours, non-training versus training facilities, payment structure, and operational agreements with other health systems) have been found to be associated with positive user experience with primary care.[13-16]

Much of this research is based in high-income settings. Similar research in lower-resource settings would provide crucial information to guide decision-makers in how to allocate resources to address deficiencies in system responsiveness to patients, and to the public to empower them to demand more patient-centered care. Further, in order to understand the degree to which experience measures are reliable indicators of the performance of healthcare systems (rather than a more limited indicator of variability in provider-patient relationships by individual-level socio-

For peer review only - http://bmjopen.bmj.com/site/about/guidelines.xhtml

demographic factors), it is critical to conduct analyses on the variability of patient experience attributable to health services factors.

Consistent with global initiatives, Mexico has a strong national commitment to both primary care and quality improvement to ensure the promises of universal access are achieved.[17, 18] Since 2009, user satisfaction/experience surveys are regular practice for public health providers in México. The Mexican Institute of Social Security (IMSS) is the largest provider, covering more than 63 million formal labor market employees and their families.[19] IMSS conducts annual National Satisfaction Surveys; yet, their results have not been analyzed comprehensively.

In this paper, we take advantage of this robust data collection effort by IMSS to investigate the degree to which variability in patient experience is due to clustering at facility and state levels (general contextual effects) and the patient, facility, and geographic factors influencing experience with health care in the IMSS primary care system (i.e., individual level and specific contextual effects).

METHODS

Data sources

Our primary data set comes from the November 2016 IMSS National Satisfaction Survey (ENSAT). IMSS regularly conducts these cross-sectional surveys with its beneficiaries over 18 years old at primary, secondary, and tertiary care services. This analysis focuses on patients at the primary care level. The survey's sampling design consists of a two-stage stratified probabilistic sampling where the probability of selecting a health facility is defined by region and facility size. The number of patients to be interviewed in each facility is estimated according to the average number of daily consultations with physicians and the proportion of patients satisfied in the previous survey. Patient recruitment is done through systematic sampling, with a random starting point and a fixed interval based on the estimated sample size and the average number of daily consultations in the clinic. A private firm (Berumen and Associates) conducted the survey. Trained interviewers carried out direct, structured interviews with IMSS health services users. The interviewers used a satisfaction questionnaire that the Center for Evaluation Research and Surveys of the National Institute of Public Health had previously validated. The response rate in the survey was 87%.

To perform the analysis, we merged 2016 ENSAT database with a facility-level database of a 2016 physical inventory of health care facilities and a 2016 record of patients affiliated with IMSS in each facility. Finally, a 2014 database of the National Council for the Evaluation of Social Development Policy (Spanish acronym CONEVAL) on the percentage of the population in each state living in poverty was merged into patient-level data.[20] Poverty is calculated by CONEVAL as a multidimensional measure based on criteria related to housing, household services, education, health care, social security, food, and per capita income.

Patient experience outcomes

We examined four binary dependent variables. The first is a composite measure of verbal and non-verbal communication, designated as positive if the patient answered favorably to three binary questions (translated from Spanish): "Did the staff who attended to you make eye contact

For peer review only - http://bmjopen.bmj.com/site/about/guidelines.xhtml

while greeting you?"; "Did the staff who attended to you listen to you with attention and without interruptions?"; and "Did the staff who attended to you respond clearly to your questions?" We also examined the questions: "Did you have the opportunity to tell the doctor or nurse what was bothering you about your health?" (referred to hereafter as "opportunity to share health concerns"); "Did you end up with doubts about the treatments necessary for your condition?" (reverse coded and referred to hereafter as "resolution of doubts"); and "How was the treatment you received in this facility during today's visit?", scored on a scale of 1-5 with 1 being "excellent," and 5 being "Terrible," collapsed to Excellent versus all other responses and referred to hereafter as the global measure.

Explanatory variables

In identifying independent variables to include in our models, we conceptualized patient experiences with IMSS healthcare delivery as being potentially influenced by factors at multiple levels (Figure 1). Available individual-level variables in the IMSS dataset included age (categorized into quartiles); sex (binary); and education level (high school or higher, less than high school, and less than primary school). We hypothesized that patients' age, sex, and educational status will explain some variance in reported experience due to differential treatment by providers and different expectations for care based on these factors. We also hypothesized experiences would vary by the primary reason for the visit, independent of patient characteristics, given that the nature of what is covered in a visit likely influences how providers approach patients (for example, primary care doctors are less comfortable discussing stigmatized issues such as sexual health and therefore exhibit poorer communication skills) and how well the visit can meet the patient's needs (for example, if their issue has numerous treatment options they may have a better experience than for those issues where treatments are limited). We therefore included the variables of self-reported reason for visit, collapsed from 149 categories into 10 categories based on ICD-10 codes, and type of consultation (curative visit, preventive care visit at the PREVENIMSS module at each clinic,[21] or dental visit). PREVENIMSS is a Spanish acronym for "Integrated Preventive Care Program," an initiative that provides targeted preventive care services to individuals based on their age and gender.

[Figure 1]

At the facility level, we hypothesized that size of facility could impact processes of care and included two variables that served as proxies for size: total number of rooms for all types of services and the number of people affiliated with a primary care clinic. We also included variables related to the presence or absence of programs that are designed to improve quality of care: presence of a special diabetes program comprising multidisciplinary teams of health-professionals (DIABETIMSS); presence of nurses' rooms attached to family doctors' consultation rooms; and availability of a nutritionist. Finally, we hypothesized that experience would vary by the percentage of population in a state that is living in poverty, given that this is likely reflective of the infrastructure in the state—including healthcare infrastructure. We thus included poverty rate in the facility's state as an independent variable. Continuous variables (consultation rooms, number of affiliates, poverty rate) were rescaled to fall on a scale of minimum 0 to maximum 1 to facilitate comparison across explanatory variables.

Analysis

For peer review only - http://bmjopen.bmj.com/site/about/guidelines.xhtml

We performed a series of analyses to address questions about the extent of clustering in each outcome (general contextual effects) and the associations of state and facility factors with each outcome (specific contextual effects) [22]. We first constructed a conventional logistic regression model for each dependent variable, including all patient-level explanatory variables of interest (Model 1). We next added a random intercept for health facility (Model 2) and subsequently the facility-level factors that could explain the measured outcomes (Model 3). In the last set of models, we added a state-level random effect (Model 4) and then state-level poverty (Model 5).

For each model, we calculated the area under the receiver operating characteristic curve (AUC). The AUC is a measure of discriminatory accuracy, i.e. how well a given model predicts individual outcomes. Comparing the AUC across models as contextual effects are added supports assessment of how the addition of each random effect improves the model's predictive capability. For random intercept models, we calculated the intra-class correlation (ICC) to determine the amount of the total variance in the outcome due to differences at the facility level (Model 2) and the facility and state levels (Model 4).

For specific contextual effects, we calculated the proportional change in variance (PCV) values between steps to allow for examination of the proportion of the contextual variance that is explained by the facility-level and then state-level covariates included in the model. To interpret the association of facility- and state-level factors with the outcomes, we calculated population average ORs. Random intercept models allow for inference on the within-cluster difference associated with each covariate. Population average ORs allow for comparing subjects from different clusters (i.e., states) that have identical observed values for other covariates. We also examined interval odds ratios (IOR) at facility and state levels to compare the magnitude of each higher-level covariate to the random effect. For instance, a facility IOR provides the range of odds ratios comparing individuals with a one unit difference on the facility or state covariate from the 10th percentile to the 90th percentile of the random effect.[23, 24]

The IMSS dataset included weights to account for non-response. To account for missing data, we weighted complete cases based on inverse probability weights. [25] Weights were calculated as the inverse of the probability of being a complete case predicted from regressing a binary indicator of completeness on patient-level covariates with no missing values (all except reason for visit and education level). The characteristics of the incomplete cases and the distribution of the inverse probability weights is shown in Supplemental Table 1 and Supplemental Figure 1; all measured individual covariates and the distribution of the weights were highly similar between complete and incomplete cases. State-level weights were set to 1; patient weights were rescaled around the mean weight at each facility. [26]

See Supplemental Figure 2 for all formulas. Stata version 14 was used for all analyses.

Ethical review

This secondary analysis of de-identified data was approved by IMSS Research and Ethics Committee (No. R 2018-785-037).

Patient and public involvement

Patients nor the public were involved in the design, conduct, or analysis of this study.

For peer review only - http://bmjopen.bmj.com/site/about/guidelines.xhtml

RESULTS

A total of 27,338 patients were surveyed from 319 primary care facilities in 32 states; 25,745 of these provided complete data (94.2%, Figure 2). The median age of respondents was 49 years, 71% were female, and 70% had less than a high school education (Table 1). A small minority of the visits represented preventive (8%) or dental (2%) care, versus curative (90%). The most common reasons for the visit were diseases of the circulatory system (20%) or endocrine, nutritional, and metabolic diseases (19%). At the facility level, the average number of rooms in clinics was 20 (range=1-57), and the median number of enrolled patients was 83,691 (range=2,356-468,618). A minority had nurses rooms attached (6%), the DIABETIMSS program (19%), or nutritionists (39%). Participants lived in states where 41% of the population, on average, lived in poverty.

[Figure 2]

[Table 1]

The majority of patients reported the staff greeted them by looking them in their eye (82%), responded clearly to their questions (92%), and listened to them with attention and without interruptions (91%) (Figure 3). 78% reported experiencing all three of these indicators of good verbal or non-verbal communication. 91% reported having the opportunity to share health concerns; 85% reported ending up with no doubts about their treatment. In 29% of visits, respondents reported excellent care overall (Figure 4).

[Figure 3]

[Figure 4]

Examination of ICC and AUC values allowed us to consider general contextual effects of clustering at the facility and state levels. In terms of the ICC, the amount of individual variation in propensity to report positive experiences that was due to systematic differences between facilities rather than systematic differences between patients ranged from 6% for both verbal/non-verbal communication and opportunity to share health concerns to 12% for the global measure. Variation due to differences between states was 6% for the global measure and 3% for the other measures (Table 2). The increase in AUC values between the single-level model with covariates and the model adding the facility random effect demonstrates that accounting for systematic differences between facilities increased predictive validity of the model by 8 to 12% (Table 2); conversely, the addition of the random effect for state did not result in an increase in AUC values, suggesting that accounting for systematic differences between states did not improve the models' ability to correctly predict individual outcomes. Roughly two thirds of individual outcomes were correctly predicted by the final models across the four outcomes.

[Table 2]

Based on the PCV values (Table 3), measured covariates at the facility level explained between 2% (global rating of visit) and 18% (opportunity to share health concerns) of the change in contextual variance. State poverty level explained a maximum of 3% of the change in variance. In the final multivariable models (Model 5 with state and facility covariates and random effects included), individuals within the same facility had higher odds of reporting positive

For peer review only - http://bmjopen.bmj.com/site/about/guidelines.xhtml

For peer review only

communication if they were male (AOR 1.18, 95% CI 1.11, 1.26), 49-62 or 63-99 years compared to 18-33 years of age (AOR 1.16 and 1.30, 95% CI 1.01, 1.32 and 1.12, 1.50; respectively), attended a visit related to pregnancy, childbirth, or the post-partum period (AOR 1.30, 95% CI 1.02, 1.67), or were seeking dental services (AOR 1.37, 95% CI 1.05, 1.79) after adjusting for other individual, facility, and state characteristics (Table 3; see output from Models 1-4 in Supplemental Table 2). More educated patients (with high school education or higher compared to less than primary school) within the same facility had lower odds of reporting high-quality communication, controlling for other covariates (AOR 0.84, 95% CI 0.76, 0.94).

[Table 3]

The pattern for other outcomes was slightly different. Individuals within the same facility had lower odds of reporting they were given the opportunity to share health concerns if they were 34-48, 49-62, and 63-99 years compared to 18-33 years (AOR 0.83 and 0.83 and 0.82, 95% CI 0.72, 0.95 and 0.72, 0.97 and 0.70, 0.96; respectively); being seen for visits categorized in ICD-10 codes as injury, poisoning and other consequences of external causes (AOR 0.62, 95% CI 0.50, 0.77) or "factors influencing health status or contact with health services" (0.38, 95% CI 0.30, 0.49); or if they were being seen in a preventive care versus curative visit (AOR 0.27, 95% CI 0.24, 0.30). Individuals within the same facility had lower odds of reporting resolution of doubts if they were being seen for symptoms, signs and abnormal clinical and laboratory findings, not elsewhere classified (AOR 0.58, 95% CI 0.48, 0.72), being seen for injury, poisoning and other consequences of external causes (AOR 0.70, 95% CI 0.52, 0.95), being seen for diseases of the musculoskeletal system and connective tissue (AOR 0.74, 95% CI 0.57, 0.97), or being seen for other reason (AOR 0.69, 95% CI 0.57, 0.83). Individuals within the same facility had higher odds of resolution of doubts if they had high school education or higher compared to less than primary school (AOR 1.18, 95% CI 1.05, 1.34) or were being seen for a preventive versus curative visit (AOR 1.26, 95% CI 1.05, 1.50), controlling for other covariates. Finally, individuals within the same facility had higher odds of reporting overall excellent care if they were 49-62 and 63-99 years compared to 18-33 years (AOR 1.23 and 1.22, 95% CI 1.10, 1.38 and 1.07, 1.39; respectively) or if they had a primary school or high school education compared to less than primary school (AOR 1.13 and 1.35, 95% CI 1.01, 1.26 and 1.16, 1.57; respectively).

Across all facilities, larger patient population size affiliated with clinic was associated with lower odds of positive communication, opportunity to share concerns, and resolution of doubts in the adjusted models (AORs comparing the smallest population size to the largest 0.32 and 0.43 and 0.48; 95% CI 0.13, 0.74 and 0.21, 0.90 and 0.25, 0.93; respectively). The only facility-level IOR that did not include the null was patient population size (for all individual experience measures, but not the global measure), suggesting that this was the only measured facility-level characteristic whose association with patient experience was strong compared to remaining facility-level heterogeneity, conditional on state. For all other facility-level variables and for state poverty, IORs included the null.

DISCUSSION

This novel analysis of patient experiences with primary care in Mexico identified generally positive perspectives on health care and found that up to 12% of variation in experience occurs at the facility level and up to 6% occurs at the state level. Models accounting for individual, facility, and state characteristics correctly classified outcomes for two in three individuals,

For peer review only - http://bmjopen.bmj.com/site/about/guidelines.xhtml

suggesting that these methods improve our insight on patient experience while pointing to the remaining unexplained variation. This suggests that quality improvement at the facility or regional level has the potential to improve individual experiences with care, though the degree of variation we uncovered at these higher levels is lower than that identified in other studies. For example, a study in general practice in Canada found that 20% of variance in perception of accessibility was attributable to practice-level characteristics[15], and a patient survey among primary care patients in California, United States, found that 28-48% was due to system related factors.[27]

While smaller patient population size was clearly associated with better patient experience, only 2 to 18% of facility-level variation could be explained by measured covariates, suggesting further research is necessary in Mexico to identify levers for improvement. In the QUALICOPC study, conducted in 34—mainly European—countries, physicians providing a broader range of services and being paid through capitation had patients that reported more positive experiences, and more robust national primary care structure and higher health spending were also factors associated with better experience [16]. In-depth exploration of high-performing facilities in Mexico would help provide insights into these and other characteristics of facilities and the broader health system that may influence patient experience. Individual characteristics shaped experience ratings, reinforcing the importance of patients' expectations of care and the potential for differential treatment by providers to impact experience. Systematic assessment of patient expectations and provider bias may help to inform health system improvements.

The finding that larger patient population affiliated with clinic was correlated with worse reported patient experience on a number of dimensions is consistent with findings from other research [14] and suggests that particular attention may need to be paid to improving patient experience in settings with high patient volume.

The pattern of relationships between individual-level characteristics and patient experience was variable across outcomes. Males reported more positive communication than females; this may reflect a difference in how providers communicate with males or different expectations for care.[28] Older individuals tended to report more positive experiences overall and specifically with communication, consistent with a review of the impact of individual characteristics on experiences.[11] Older individuals were less likely than those between 18-33 years to report opportunity to share their health concerns, again either representing a difference in communication style on the part of providers or different expectations for care by patients by age. In terms of education, more educated individuals were less likely to report positive communication, consistent with other research and likely due to higher expectations for care.[11] However, they were more likely to report resolution of doubts and positive overall experiences. This could represent the fact that those with the most education are empowered to be more "active" participants in their care,[29] or possible provider discrimination against patients with less than primary school.

The reason for a patient's visit was consistently related to their experience, with preventive care visits eliciting higher ratings for resolution of doubts (though lower ratings for opportunity to share concerns), and dental visits being associated with better communication. This may reflect the different nature of preventive care, wherein ability to resolve patients' doubts is more achievable than when patients are being seen for a particular medical concern. It could, however, reflect better quality care for patients in the preventive care or dental modules. Specific

For peer review only - http://bmjopen.bmj.com/site/about/guidelines.xhtml

conditions were also associated with report of better experiences in some cases, also likely reflecting either the nature of the visit or systematic differences in the ways providers treat patients who are being seen for different conditions.

These findings should be considered in light of several limitations. First, the data are cross-sectional and we are unable to draw causal inferences from the observed associations between independent and dependent variables. Further, though we were able to identify the overall amount of variance attributable to the facility and state levels, we had few potential explanatory factors available at those levels and could not explain the majority of the contextual variation. Second, available datasets did not indicate which providers patients had seen, keeping us from being able to parse the variance in patient experience attributable to differences between providers. Future efforts to collect national experience data in the IMSS system could benefit from collecting these data. Third, we did not have access to information on all IMSS primary care visits to be able to compare the demographics of our sample with the overall population of users. However, the response rate was high (87%), particularly compared to other patient experience surveys such as the Consumer Assessment of Healthcare Providers and Systems in the United States [30], and the rate of missing data was fairly low (6%) suggesting reasonable representativeness to the population of IMSS users. Finally, the results are generalizable only to users of the IMSS system, as the survey did not include patients from other health sectors in Mexico.

Our findings shed light on the influences at individual, facility, and state levels on patients' experiences with a large public health system in Mexico. These findings can inform efforts to improve patient experiences, thereby promoting confidence in the health system and better health outcomes. Key priorities for future work include further investigation of the facility-level factors accounting for differences in patient experience, as well as more robust monitoring of patients' expectations for care and their potential experiences with bias to better understand what accounts for individual level differences in experience.

For peer review only - http://bmjopen.bmj.com/site/about/guidelines.xhtml

Table 1. Patient and facility characteristics

	n (%)
**Patient characteristics (N=25,745)**	
Sex	
Male	7,533 (29.3)
Female	18,212 (70.7)
Age	
Median, Range (Interquartile range)	49, 18-99 (34-63)
Level of education	
Less than primary school	4,006 (15.6)
Less than high school	13,914 (54.0)
High school or more	7,825 (30.4)
Visit type	
Curative	23,144 (89.9)
Preventive	2,024 (7.9)
Dental	577 (2.2)
Reason for visit	
Diseases of the circulatory system	5,132 (19.9)
Endocrine, nutritional and metabolic diseases	4,898 (19.0)
Other	3,085 (12.0)
Diseases of the respiratory system	2,386 (9.3)
Factors influencing health status and contact with health services	1,805 (7.0)
Pregnancy, childbirth and the puerperium	1,781 (6.9)
Diseases of the musculoskeletal system and connective tissue	1,710 (6.6)
Diseases of the digestive system	1,691 (6.6)
Symptoms, signs and abnormal clinical and laboratory findings, not elsewhere classified	1,664 (6.5)
Injury, poisoning and certain other consequences of external causes	1,593 (6.2)
Percent poverty in state	
Mean (Range), SD	41.1 (20.4-76.2), 12.9
**Facility characteristics (N=319)**	
Number of consultation rooms	
Mean (Range), SD	20.4 (1-57), 13.0
Diabetes program	
No diabetes program	258 (80.9)
Has diabetes program	61 (19.1)
Nutritionist	
No nutritionist	194 (60.8)
At least one nutritionist	125 (39.2)
Nurses room	

For peer review only - http://bmjopen.bmj.com/site/about/guidelines.xhtml

No nurse room	300 (94.0)
At least one nurse room	19 (6.0)
Patient population size	
Median (Range), SD	83,691 (2,356-468,618), 86,231.0

Table 2. General contextual effects

	Positive verbal/non-verbal communication	Opportunity to share health concerns	Resolution of doubts	Excellent global experience rating
**Intraclass correlation (ICC)**				
Addition of facility RE				
Facility level coefficient	0.06	0.05	0.08	0.10
Addition of facility & state RE				
Facility level coefficient	0.06	0.06	0.08	0.12
State level coefficient	0.03	0.03	0.03	0.06
**Area under curve (AUC)**				
Single level with covariates	0.56 (0.55-0.56)	0.63 (0.61-0.64)	0.56 (0.55-0.57)	0.54 (0.53-0.55)
Addition of facility RE	0.64 (0.63-0.65)	0.71 (0.70-0.72)	0.67 (0.66-0.68)	0.66 (0.65-0.66)
Addition of facility & state RE	0.64 (0.63-0.65)	0.70 (0.69-0.71)	0.67 (0.66-0.67)	0.65 (0.65-0.66)

RE=random effect

For peer review only

For peer review only - http://bmjopen.bmj.com/site/about/guidelines.xhtml

Table 3. Multi-level logistic regression results: Adjusted odds ratios (AORs) with 95% confidence intervals (n=25,745 patients, N= 319 facilities, N=32 states)

	Positive verbal/non-verbal communication	Opportunity to share health concerns	Resolution of doubts	Excellent global experience rating
**Individual Level Variables**	AOR (95% CI)	AOR (95% CI)	AOR (95% CI)	AOR (95% CI)
Male sex	**1.18 (1.11, 1.26)**	1.06 (.94, 1.21)	.98 (.87, 1.12)	1.07 (.98, 1.17)
Age				
18-33 years	Ref	Ref	Ref	Ref
34-48 years	1.08 (.94, 1.23)	**.83 (.72, .95)**	.90 (.79, 1.02)	1.04 (.93, 1.16)
49-62 years	**1.16 (1.01, 1.32)**	**.83 (.72, .97)**	.95 (.81, 1.11)	**1.23 (1.10, 1.38)**
63-99 years	**1.30 (1.12, 1.50)**	**.82 (.70, .96)**	.98 (.85, 1.14)	**1.22 (1.07, 1.39)**
Level of education				
Less than primary school	Ref	Ref	Ref	Ref
Less than high school	.90 (.80, 1.02)	1.01 (.81, 1.25)	1.07 (.96, 1.19)	**1.13 (1.01, 1.26)**
High school or more	**.84 (.76, .94)**	1.14 (.92, 1.41)	**1.18 (1.05, 1.34)**	**1.35 (1.16, 1.57)**
Visit type				
Curative	Ref	Ref	Ref	Ref
Preventive	1.02 (.85, 1.22)	**.27 (.24, .30)**	**1.26 (1.05, 1.50)**	1.07 (.88, 1.30)
Dental	**1.37 (1.05, 1.79)**	1.09 (.73, 1.64)	1.22 (.89, 1.68)	1.21 (.91, 1.62)
Reason for visit				
Diseases of the circulatory system	Ref	Ref	Ref	Ref
Endocrine, nutritional and metabolic diseases	1.02 (.88, 1.18)	1.01 (.82, 1.24)	.88 (.73, 1.06)	.95 (.83, 1.09)
Other	.89 (.75, 1.04)	.87 (.72, 1.05)	**.69 (.57, .83)**	.95 (.81, 1.12)
Diseases of the respiratory system	.94 (.82, 1.08)	1.16 (.84, 1.61)	.96 (.76, 1.23)	.95 (.82, 1.09)
Factors influencing health status and contact with health services	1.12 (.90, 1.38)	**.38 (.30, .49)**	1.03 (.82, 1.30)	1.14 (.93, 1.39)
Pregnancy, childbirth and	**1.30 (1.02, 1.67)**	.93 (.73, 1.20)	.89 (.71, 1.10)	1.08 (.88, 1.34)

For peer review only - http://bmjopen.bmj.com/site/about/guidelines.xhtml

the puerperium				
Diseases of the musculoskeletal system and connective tissue	.82 (.66, 1.02)	.82 (.65, 1.03)	**.74 (.57, 0.97)**	1.01 (.89, 1.20)
Diseases of the digestive system	.88 (.73, 1.06)	.86 (.64, 1.14)	.75 (.55, 1.03)	.86 (.72, 1.03)
Symptoms, signs and abnormal clinical and laboratory findings, not elsewhere classified	.89 (.71, 1.11)	.88 (.67, 1.17)	**.58 (.48, .72)**	1.04 (.87, 1.25)
Injury, poisoning and certain other consequences of external causes	.88 (.73, 1.06)	**.62 (.50, .77)**	**.70 (.52, .95)**	.90 (.74, 1.09)
**Facility Level Variables**	AOR (95% CI) IOR	AOR (95% CI) IOR	AOR (95% CI) IOR	AOR (95% CI) IOR
Number of consultation rooms				
AOR	1.65 (.88, 3.36)	.93 (.37, 2.34)	.85 (.41, 1.74)	1.04 (.51, 2.10)
IOR	0.92, 2.95	0.50, 1.72	0.45, 1.60	0.43, 2.49
Presence of:				
Diabetes program				
AOR	1.02 (0.88, 1.19)	1.08 (.81, 1.45)	1.11 (.92, 1.36)	0.99 (.82, 1.19)
IOR	0.57, 1.83	0.59, 2.00	0.59, 2.10	0.41, 2.38
Nutritionist				
AOR	1.00 (.84, 1.19)	1.07 (.83, 1.40)	0.95 (.73, 1.25)	1.10 (.89, 1.35)
IOR	0.56, 1.56	0.58, 1.99	0.51, 1.79	0.46, 2.63
Nurses room				
AOR	.87 (.69, 1.10)	.97 (.67, 1.40)	.98 (.72, 1.33)	0.92 (.79, 1.08)
IOR	0.49, 1.56	0.52, 1.79	0.52, 1.84	0.38, 2.21
Patient population size				
AOR	**.32 (.13, .74)**	**.43 (.21, 0.90)**	**.48 (.25, 0.93)**	1.19 (.48, 2.91)
IOR	0.18, 0.56	0.23, 0.80	0.26, 0.90	0.49, 2.85

For peer review only - http://bmjopen.bmj.com/site/about/guidelines.xhtml

**State Level Variable**					
Percent poverty in state (minimum = 20%; versus maximum = 76%)					
	AOR	1.12 (.69, 1.82)	.82 (.50, 1.35)	.48 (.50, 1.67)	.74 (.42, 1.30)
	IOR	0.62, 2.02	0.46, 1.47	0.42, 1.97	0.33, 1.67

For peer review only

For peer review only - http://bmjopen.bmj.com/site/about/guidelines.xhtml

Author statement: KH, SD, and HL conceived of the study; KH, HL, and DL conducted the analysis; KH drafted the manuscript; SD, HL, DL, and RPC provided substantive feedback on the manuscript.

Funding statement: HL and DL received support from the Bill and Melinda Gates Foundation (Award OPP1161450, Kruk)

Acknowledgements: The authors would like to acknowledge the generous help of Sarah Wulf in preparing the tables and figures for the manuscript.

Competing interests: We have no competing interests to declare.

Data sharing statement:
Patient- and state-level data are available in public, open access repositories
Patient-level outcomes: http://datos.imss.gob.mx/
State-level poverty data:
https://www.coneval.org.mx/coordinacion/entidades/Paginas/inicioent.aspx

Facility-level data may be obtained from a third party and are not publicly available
Data on IMSS facility characteristics are available from IMSS upon request after approval for analyses from the IMSS Health Research Ethics Committee (contact: comiteeticainv.imss@gmail.com)

Figure Legends

Figure 1. Conceptual model of relationship between model variables and reported experience with provider (N=25,745 patients and 319 facilities)

Figure 2. Sample flow chart
*Average patients per facility= 81 (min=28; max=139); average facilities per state = 10 (min 5, max 23)

Figure 3. Patient report of experience with communicating with health care providers (N=25,745)

Figure 4. Patient global rating of treatment received (N=25,745)

For peer review only - http://bmjopen.bmj.com/site/about/guidelines.xhtml

REFERENCES CITED

1. Pettigrew, L.M., et al., *Primary health care and the Sustainable Development Goals.* The Lancet, 2015. **386**(10009): p. 2119-2121.
2. Stigler, F.L., et al., *No universal health coverage without primary health care.* The Lancet, 2016. **387**(10030): p. 1811.
3. Van Lerberghe, W., *The world health report 2008: primary health care: now more than ever*. 2008: World Health Organization.
4. Kruk, M.E., et al., *High-quality health systems in the Sustainable Development Goals era: time for a revolution.* The Lancet Global Health, 2018.
5. Doubova, S.V., et al., *Attributes of patient-centered primary care associated with the public perception of good healthcare quality in Brazil, Colombia, Mexico and El Salvador.* Health policy and planning, 2016. **31**(7): p. 834-843.
6. Guanais, F., et al., *Patient-centered primary care and self-rated health in 6 Latin American and Caribbean countries: Analysis of a public opinion cross-sectional survey.* PLoS medicine, 2018. **15**(10): p. e1002673.
7. Birhanu, Z., et al., *Determinants of satisfaction with health care provider interactions at health centres in central Ethiopia: a cross sectional study.* BMC health services research, 2010. **10**(1): p. 78.
8. Doyle, C., L. Lennox, and D. Bell, *A systematic review of evidence on the links between patient experience and clinical safety and effectiveness.* BMJ open, 2013. **3**(1): p. e001570.
9. Batbaatar, E., et al., *Determinants of patient satisfaction: a systematic review.* Perspectives in public health, 2017. **137**(2): p. 89-101.
10. Hearld, L.R., et al., *How do hospital organizational structure and processes affect quality of care? A critical review of research methods.* Medical Care Research and Review, 2008. **65**(3): p. 259-299.
11. Sandager, M., M. Freil, and J.L. Knudsen, *Please tick the appropriate box: Perspectives on patient reported experience.* Patient Experience Journal, 2016. **3**(1): p. 63-79.
12. Salisbury, C., M. Wallace, and A.A. Montgomery, *Patients' experience and satisfaction in primary care: secondary analysis using multilevel modelling.* Bmj, 2010. **341**: p. c5004.
13. Cohidon, C., P. Wild, and N. Senn, *Patient experience in primary care: association with patient, physician and practice characteristics in a fee-for-service system.* Swiss medical weekly, 2018. **148**.
14. Heje, H.N., et al., *Doctor and practice characteristics associated with differences in patient evaluations of general practice.* BMC health services research, 2007. **7**(1): p. 46.
15. Haggerty, J.L., et al., *Practice features associated with patient-reported accessibility, continuity, and coordination of primary health care.* The Annals of Family Medicine, 2008. **6**(2): p. 116-123.
16. Schäfer, W.L.A., et al., *Are people's health care needs better met when primary care is strong? A synthesis of the results of the QUALICOPC study in 34 countries*. Primary Health Care Research & Development, 2019. 20(e104): 1–9. doi:10.1017/S1463423619000434
17. Atun, R., et al., *Health-system reform and universal health coverage in Latin America.* The Lancet, 2015. **385**(9974): p. 1230-1247.
18. Doubova, S.V., et al., *Quality governance in a pluralistic health system: Mexican experience and challenges.* The Lancet Global Health, 2018. **6**(11): p. e1149-e1152.
19. Gobierno de los Estados Unidos Mexicanos. Cuarto Informe de Gobierno 2015-2016. Anexo estadístico. Ciudad de México 2016. Available at: http://www.presidencia.gob.mx/cuartoinforme/
20. Consejo Nacional de Evaluación de la Política de Desarrollo Social. Información de pobreza y evaluación en las entidades federativas. Mexico: CONEVAL, 2014. Available at

For peer review only - http://bmjopen.bmj.com/site/about/guidelines.xhtml

http://www.coneval.org.mx/coordinacion/entidades/Paginas/inicioent.aspx

21. Gutiérrez, G., et al., *Strengthening preventive care programs: a permanent challenge for healthcare systems; lessons from PREVENIMSS México.* BMC public health, 2010. **10**(1): p. 417.
22. Merlo, J., Wagner, P., Ghith, N. and Leckie, G., *An original stepwise multilevel logistic regression analysis of discriminatory accuracy: the case of neighbourhoods and health*. PLoS One, 2016. **11**(4), p.e0153778.
23. Austin, P.C. and J. Merlo, *Intermediate and advanced topics in multilevel logistic regression analysis.* Statistics in medicine, 2017. **36**(20): p. 3257-3277.
24. Larsen, K. and J. Merlo, *Appropriate assessment of neighborhood effects on individual health: integrating random and fixed effects in multilevel logistic regression.* American journal of epidemiology, 2005. **161**(1): p. 81-88.
25. Seaman, S.R. and White, I.R., *Review of inverse probability weighting for dealing with missing data. Statistical methods in medical research*, 2013, **22**(3), pp.278-295.
26. Rabe-Hesketh, S. and A. Skrondal, Multilevel modeling of complex survey data. Journal of the Royal Statistical Society, 2006. Series A 169, 805-827.
27. Rodriguez, H.P., et al., *Attributing sources of variation in patients' experiences of ambulatory care.* Medical care, 2009: p. 835-841.
28. Roder-DeWan, S., et al., *Good ratings for poor quality: Low healthcare expectations among internet users in 12 low- and middle-income countries*. In Press
29. Leonard, K.L., *Active patients in rural African health care: implications for research and policy.* Health policy and planning, 2013. **29**(1): p. 85-95.
30. Tesler, R. and Sorra, J., 2019. CAHPS survey administration: what we know and potential research questions. Available at: https://www.ahrq.gov/sites/default/files/wysiwyg/cahps/about-cahps/research/survey-administration-literature-review.pdf

For peer review only

Figure 1. Conceptual model of relationship between model variables and reported experience with provider (N=25,745 patients and 319 facilities)

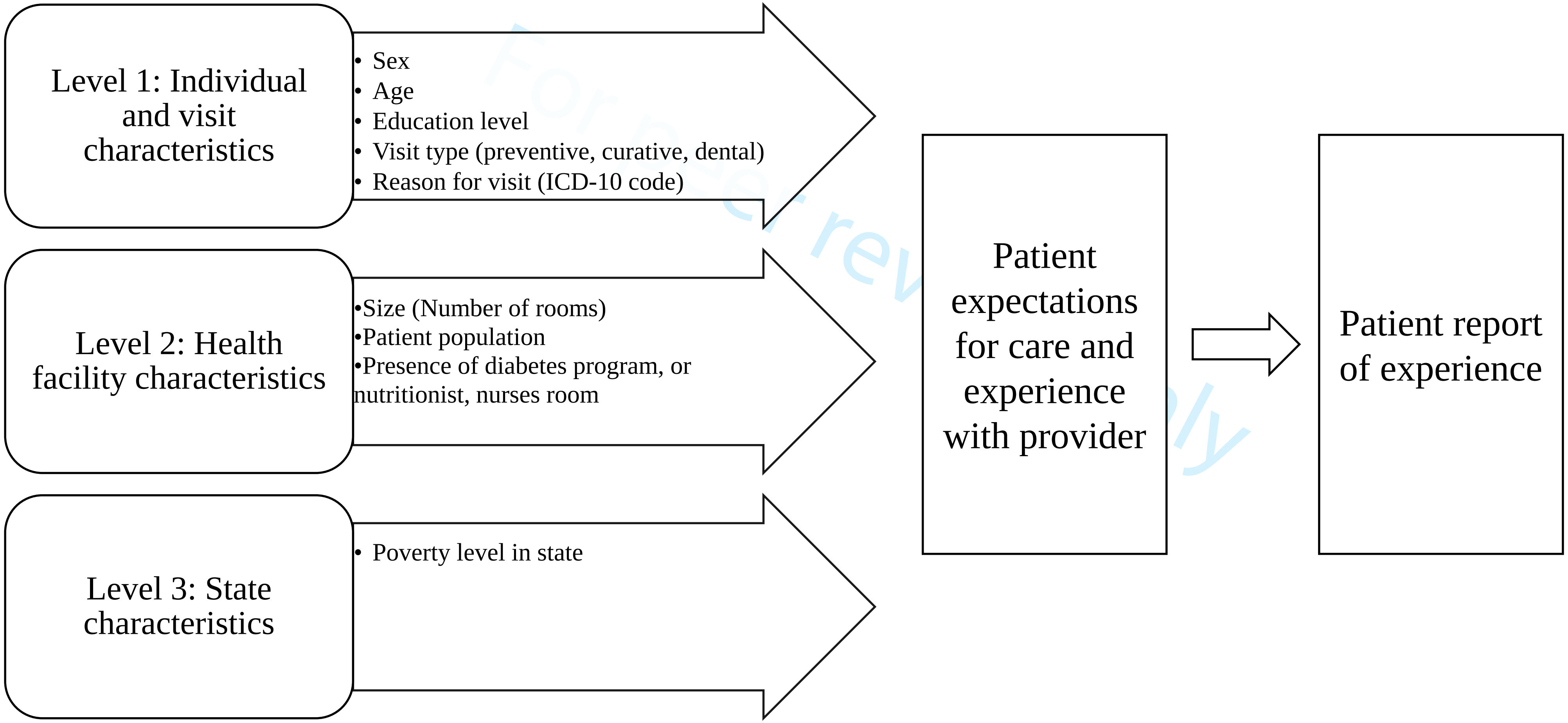

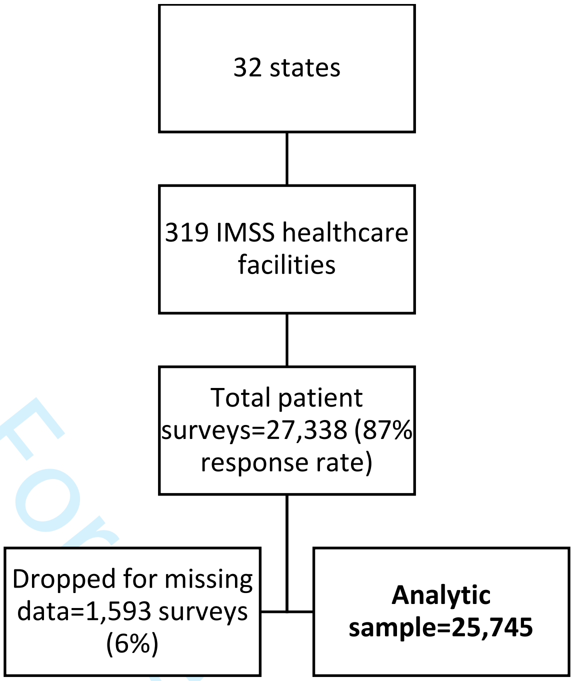

Figure 2: Sample flow chart
*Average patients per facility= 81 (min=28; max=139); average facilities per state = 10 (min 5, max 23)

For peer review only

Figure 3. Patient report of experience with communicating with health care providers (N=25,745)

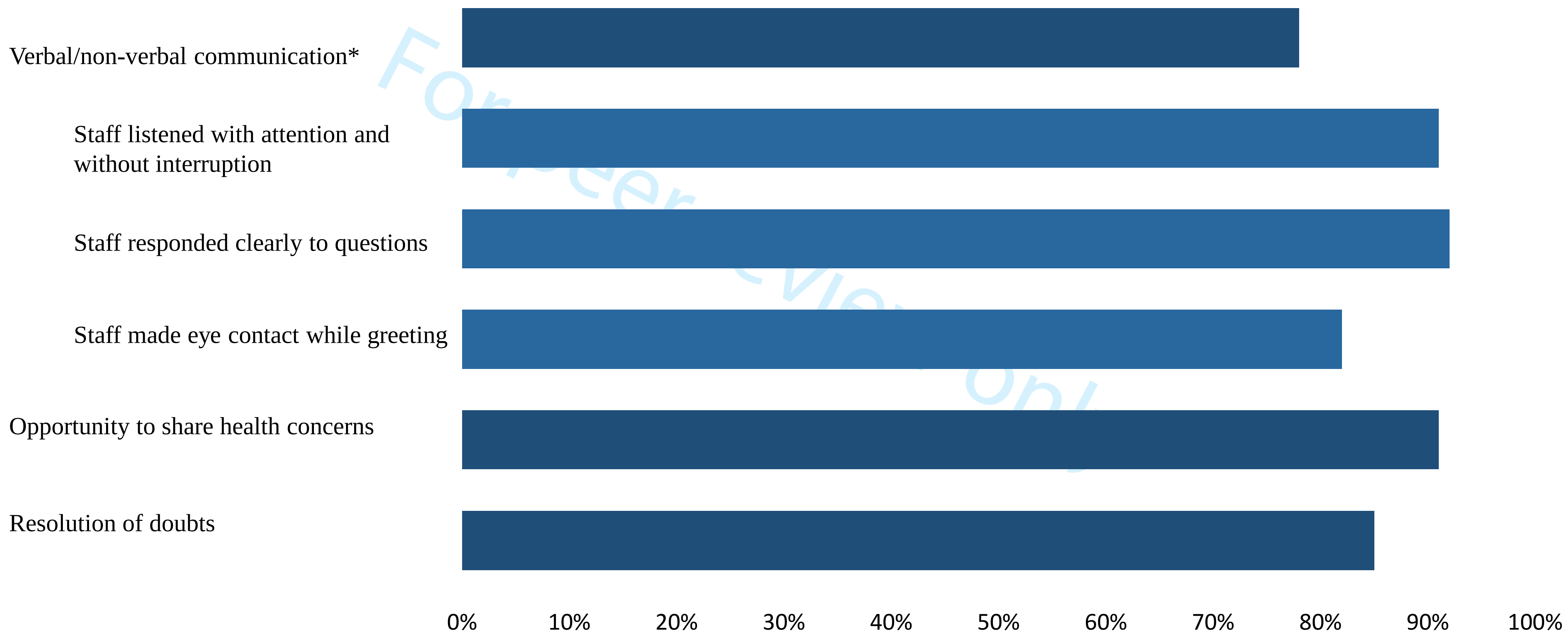

*Verbal/non-verbal communication is a composite variable comprised of those who reported positively to all three of the listed variables.

For peer review only - http://bmjopen.bmj.com/site/about/guidelines.xhtml

Figure 4. Patient global rating of treatment received (N=25,745)

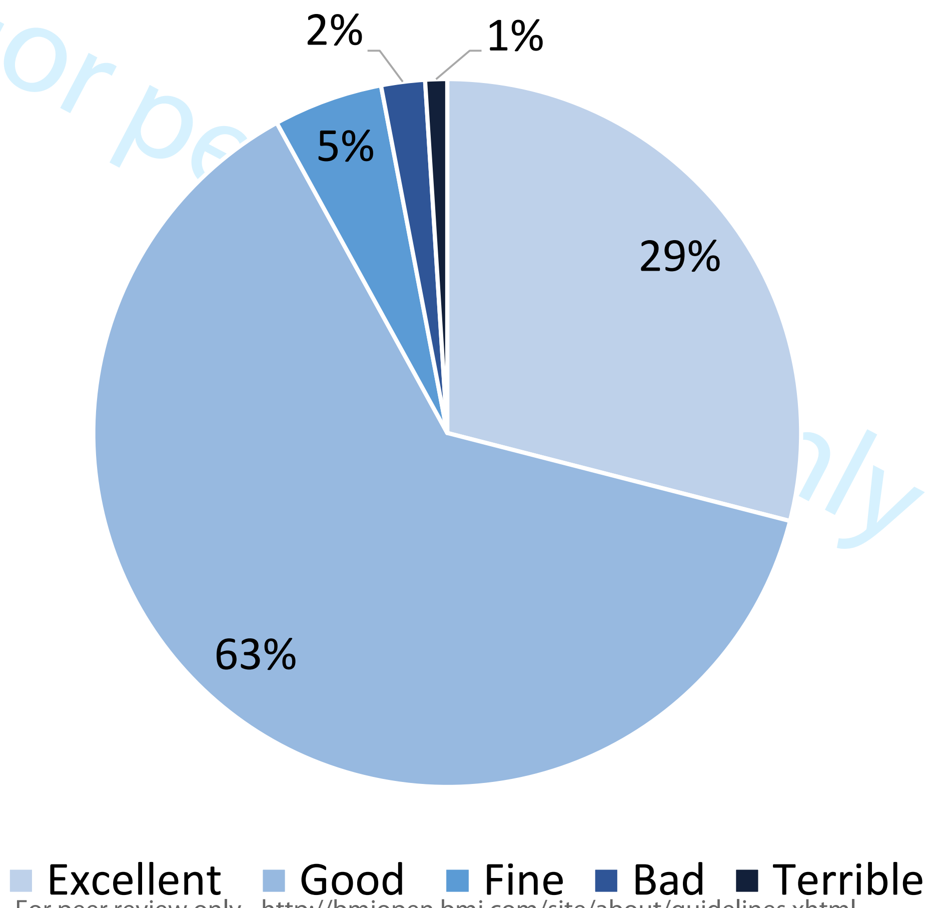

For peer review only - http://bmjopen.bmj.com/site/about/guidelines.xhtml

Supplemental Information, **Factors associated with positive user experience with primary healthcare providers in Mexico: A multi-level modeling approach using national cross-sectional data**

Supplemental Table 1: Characteristics of Respondents for Incomplete and Complete Cases

	Incomplete (N = 1592)	Complete (N = 25745)	Total (N = 27337)
Sex of Patient			
Female	1123 (71%)	18313 (71%)	19436 (71%)
Male	469 (29%)	7432 (29%)	7901 (29%)
Age			
Bottom Quartile (18 – 33)	346 (22%)	6384 (25%)	6730 (25%)
25-50 Quartile (34 – 48)	367 (23%)	6261 (24%)	6628 (24%)
50-75 Quartile (49 – 62)	420 (26%)	6555 (25%)	6975 (26%)
Top Quartile (63 – 99)	459 (29%)	6545 (25%)	7004 (26%)
Education			
Without Schooling	272 (18%)	4309 (17%)	4581 (17%)
Completed Primary School	818 (53%)	13792 (54%)	14610 (54%)
High School or higher	460 (30%)	7644 (30%)	8104 (30%)
Type of consultation:			
Medical consultation	1393 (88%)	23113 (90%)	24506 (90%)
PREVENIMS (preventive care consultation by nurses)	171 (11%)	2023 (8%)	2194 (8%)
Dental consultation	28 (2%)	609 (2%)	637 (2%)
ICD 10 Code (Grouped)			
Endocrine, nutritional and metabolic diseases	291 (20%)	4843 (19%)	5134 (19%)
Diseases of the circulatory system	314 (22%)	5128 (20%)	5442 (20%)
Diseases of the respiratory system	131 (9%)	2352 (9%)	2483 (9%)
Diseases of the digestive system	84 (6%)	1705 (7%)	1789 (7%)
Diseases of the musculoskeletal system and connective tissue	88 (6%)	1692 (7%)	1780 (7%)
Pregnancy, childbirth and the puerperium	85 (6%)	1846 (7%)	1931 (7%)
Symptoms, signs and abnormal clinical and laboratory findings, not elsewhere classified	99 (7%)	1683 (7%)	1782 (7%)
Injury, poisoning and certain other consequences of external causes	71 (5%)	1515 (6%)	1586 (6%)
Factors influencing health status and contact with health services	104 (7%)	1831 (7%)	1935 (7%)
Other	158 (11%)	3150 (12%)	3308 (12%)

For peer review only

Supplemental Figure 1: Distribution of Inverse Probability Weights for Incomplete and Complete Cases (N=25,745)

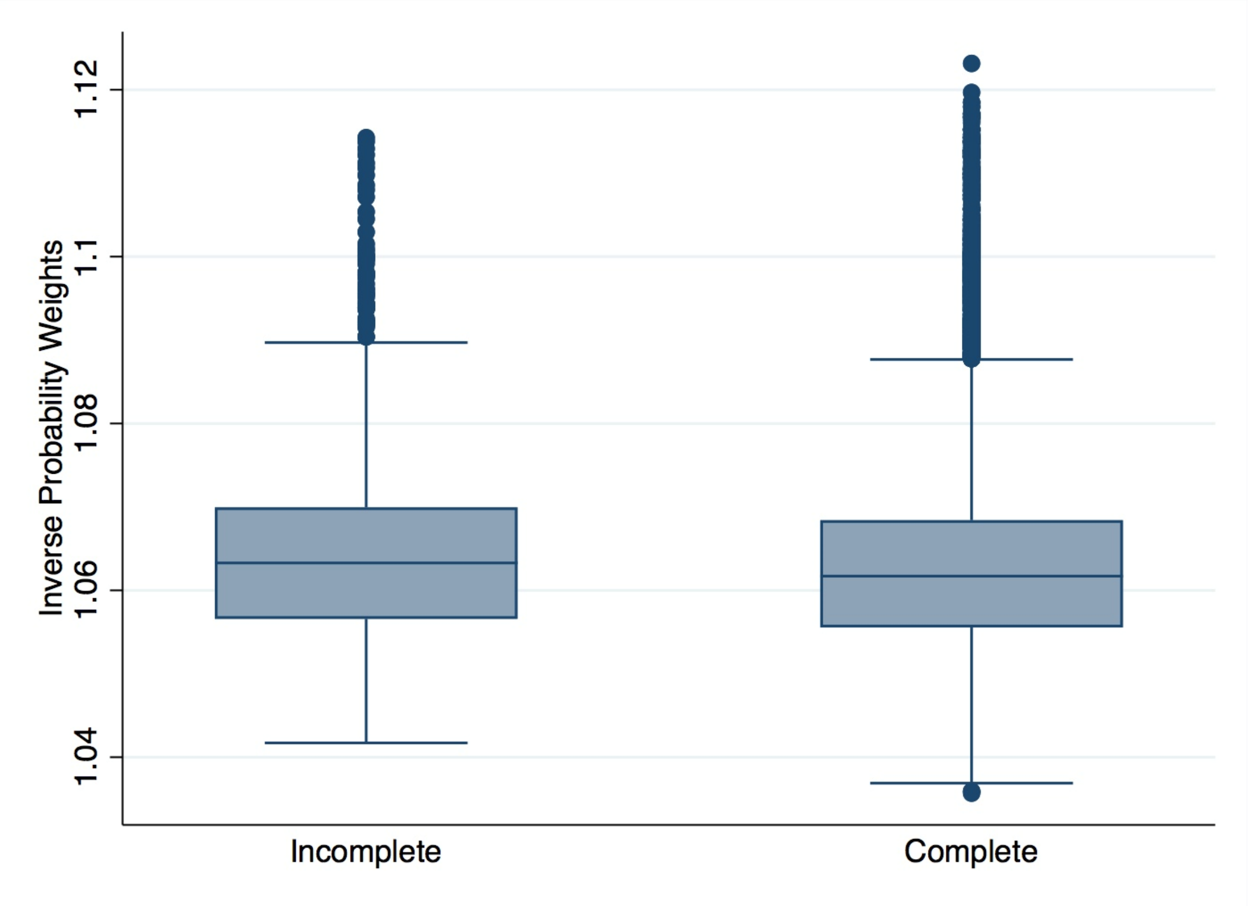

w only

Supplemental Figure 2: Formulas

ICC:

Level 2:

$$\frac{\widehat{\tau_2^2} + \widehat{\tau_3^2}}{\widehat{\tau_2^2} + \widehat{\tau_3^2} + \frac{\pi^2}{3}}$$

Level 3:

$$\frac{\widehat{\tau_3^2}}{\widehat{\tau_2^2} + \widehat{\tau_3^2} + \frac{\pi^2}{3}}$$

IOR:

Level 2:

$$\left[\exp\left(\widehat{\alpha_2} + \sqrt{2\widehat{\tau_2^2}} * \Phi^{-1}(0.1)\right), \exp\left(\widehat{\alpha_2} + \sqrt{2\widehat{\tau_2^2}} * \Phi^{-1}(0.9)\right)\right]$$

Level 3:

$$\left[\exp\left(\widehat{\alpha_3} + \sqrt{2\widehat{\tau_3^2}} * \Phi^{-1}(0.1)\right), \exp\left(\widehat{\alpha_3} + \sqrt{2\widehat{\tau_3^2}} * \Phi^{-1}(0.9)\right)\right]$$

Population Average Adjusted Odds Ratios:

Level 2:

For peer review only

$$\exp(\widehat{\alpha_{PA,2}}) = \exp\left(\frac{\widehat{\alpha_2}}{\sqrt{1 + \left(16^2 * \frac{3}{(15 * \pi)^2}\right) * \widehat{\tau_2^2}}}\right)$$

Level 3:

$$\exp(\widehat{\alpha_{PA,3}}) = \exp\left(\frac{\widehat{\alpha_3}}{\sqrt{1 + \left(16^2 * \frac{3}{(15 * \pi)^2}\right) * \widehat{\tau_3^2}}}\right)$$

Where the subscript denotes the level of the variable, and level 2 variances (facility-level) are conditional on the third level (state-level), $\widehat{\tau^2}$ is the estimated group variance, and $\hat{\alpha}$ is an estimated regression coefficient. Φ represents the cumulative distribution function of the standard normal distribution.

For peer review only

Supplemental Table 2: Models 1-4 for all outcomes

Please note coefficients are not population-adjusted

```
-----------------------------------------------------------------------------------
*I) Flat Model with Individual Covariates for Communication
Note: Stage 1 is sampled with replacement; further stages will be ignored for variance
      estimation.

      pweight: <none>
          VCE: linearized
  Single unit: missing
     Strata 1: <one>
         SU 1: unidmed
        FPC 1: <zero>
     Weight 1: facility_weight
     Strata 2: <one>
         SU 2: <observations>
        FPC 2: <zero>
     Weight 2: rescaled_patient_weights
(running logit on estimation sample)

Survey: Logistic regression

Number of strata   =         1                Number of obs     =     27,337
Number of PSUs     =       319                Population size   = 68,852.018
                                              Subpop. no. obs   =     25,745
                                              Subpop. size      = 64,704.336
                                              Design df         =        318
                                              F(  17,    302)   =       5.89
                                              Prob > F          =     0.0000
```

Communication	Coef.	Linearized Std. Err.	t	P>|t|	[95% Conf.	Interval]
age_quart						
25-50 Quartile	.0721386	.0519142	1.39	0.166	-.03	.1742772
50-75 Quartile	.1310581	.0623401	2.10	0.036	.008407	.2537091
Top Quartile	.2501662	.0746586	3.35	0.001	.103279	.3970534
sex_male	.176233	.0417178	4.22	0.000	.0941551	.2583108
education						
Completed Primary School	-.1652057	.065023	-2.54	0.012	-.2931353	-.0372761
High School or higher	-.2300984	.0663483	-3.47	0.001	-.3606354	-.0995614
icd10_bin						
Endocrine, nutritional ..	.0034759	.0622057	0.06	0.955	-.1189108	.1258626
Diseases of the respira..	-.0665038	.0706778	-0.94	0.347	-.205559	.0725515
Diseases of the digesti..	-.1280427	.1026104	-1.25	0.213	-.3299238	.0738384
Diseases of the musculo..	-.2116943	.0894267	-2.37	0.019	-.387637	-.0357515
Pregnancy, childbirth a..	.2507037	.0971022	2.58	0.010	.0596597	.4417477
Symptoms, signs and abn..	-.1040915	.0953784	-1.09	0.276	-.2917439	.0835608
Injury, poisoning and c..	-.1512712	.0883329	-1.71	0.088	-.325062	.0225196
Factors influencing hea..	.1223723	.1084912	1.13	0.260	-.0910789	.3358235
Other	-.114026	.071712	-1.59	0.113	-.2551159	.027064
service						
PREVENIMS (preventive ..)	.0203624	.0829673	0.25	0.806	-.1428716	.1835965

For peer review only

```
     Dental consultation |   .2658778   .1404628     1.89   0.059     -.010476    .5422315
                         |
                   _cons |   1.204737   .1144617    10.53   0.000     .9795395    1.429935
------------------------------------------------------------------------------------------
*II) Add RE for Facility for Communication
(running melogit on estimation sample)

Survey: Mixed-effects logistic regression

Number of strata   =         1                  Number of obs     =     27,337
Number of PSUs     =       319                  Population size   = 68,852.018
                                                Subpop. no. obs   =     25,745
                                                Subpop. size      = 64,704.336
                                                Design df         =        318
                                                F(  17,    302)   =       5.52
                                                Prob > F          =     0.0000

------------------------------------------------------------------------------------------
                         |             Linearized
           Communication |      Coef.   Std. Err.      t    P>|t|     [95% Conf. Interval]
-------------------------+----------------------------------------------------------------
                         |
               age_quart |
        25-50 Quartile   |    .074767   .0537437     1.39   0.165    -.0309712    .1805052
        50-75 Quartile   |     .13805   .0603951     2.29   0.023     .0192256    .2568743
          Top Quartile   |   .2533195   .0717119     3.53   0.000     .1122298    .3944093
                         |
                sex_male |   .1643417   .0422591     3.89   0.000      .081199    .2474844
                         |
               education |
Completed Primary School |  -.1223788   .0634736    -1.93   0.055      -.24726    .0025024
   High School or higher |  -.1935813   .0634676    -3.05   0.002    -.3184508   -.0687119
                         |
               icd10_bin |
Endocrine, nutritional ..|   .0138917   .0639729     0.22   0.828    -.1119718    .1397552
Diseases of the respira..|  -.0694817   .0749529    -0.93   0.355    -.2169481    .0779846
Diseases of the digesti..|  -.1320547   .1037108    -1.27   0.204    -.3361007    .0719913
Diseases of the musculo..|  -.2054198   .0910969    -2.25   0.025    -.3846486   -.0261909
Pregnancy, childbirth a..|   .2578102   .0993497     2.59   0.010     .0623445     .453276
Symptoms, signs and abn..|  -.1182738   .0980881    -1.21   0.229    -.3112573    .0747098
Injury, poisoning and c..|  -.1355994    .091086    -1.49   0.138    -.3148066    .0436079
Factors influencing hea..|   .0986902    .105488     0.94   0.350    -.1088524    .3062328
                   Other |  -.1227894   .0723211    -1.70   0.091    -.2650776    .0194988
                         |
                 service |
PREVENIMS (preventive ..)|   .0239104   .0816092     0.29   0.770    -.1366518    .1844726
     Dental consultation |   .3227617   .1441316     2.24   0.026     .0391897    .6063337
                         |
                   _cons |   1.277797   .1201587    10.63   0.000      1.04139    1.514203
-------------------------+----------------------------------------------------------------
unidmed                  |
               var(_cons)|   .2059121   .0336714                      .1492653    .2840565
------------------------------------------------------------------------------------------
*III) Facility-level Effects for Communication
(running melogit on estimation sample)

Survey: Mixed-effects logistic regression

Number of strata   =         1                  Number of obs     =     27,337
Number of PSUs     =       319                  Population size   = 68,852.018
                                                Subpop. no. obs   =     25,745
```

For peer review only

```
                                                Subpop. size      = 64,704.336
                                                Design df         =        318
                                                F(  22,    297)   =       6.62
                                                Prob > F          =     0.0000

------------------------------------------------------------------------------
                           |             Linearized
             Communication |      Coef.   Std. Err.      t    P>|t|     [95% Conf. Interval]
---------------------------+--------------------------------------------------------------------
                           |
                 age_quart |
            25-50 Quartile |   .0745965   .0536839     1.39   0.166    -.0310239    .1802169
            50-75 Quartile |   .1418883    .060469     2.35   0.020     .0229184    .2608581
              Top Quartile |    .258148   .0721074     3.58   0.000     .1162801    .4000158
                           |
                  sex_male |   .1617564   .0423419     3.82   0.000     .0784507     .245062
                           |
                 education |
   Completed Primary School|  -.1165275   .0630907    -1.85   0.066    -.2406555    .0076005
      High School or higher|  -.1842632   .0633158    -2.91   0.004    -.3088341   -.0596924
                           |
                 icd10_bin |
 Endocrine, nutritional .. |   .0145716   .0636643     0.23   0.819    -.1106849    .1398282
 Diseases of the respira.. |  -.0718691   .0747377    -0.96   0.337     -.218912    .0751738
 Diseases of the digesti.. |  -.1301095   .1034579    -1.26   0.209     -.333658     .073439
 Diseases of the musculo.. |  -.2021127   .0909805    -2.22   0.027    -.3811125   -.0231129
 Pregnancy, childbirth a.. |    .261052   .0993929     2.63   0.009     .0655012    .4566028
 Symptoms, signs and abn.. |  -.1214599   .0977301    -1.24   0.215    -.3137392    .0708193
 Injury, poisoning and c.. |  -.1308762   .0909873    -1.44   0.151    -.3098893     .048137
 Factors influencing hea.. |   .0939097   .1055378     0.89   0.374    -.1137307    .3015502
                     Other |  -.1229068   .0723339    -1.70   0.090    -.2652202    .0194066
                           |
                   service |
 PREVENIMS (preventive ..) |   .0204962    .081476     0.25   0.802    -.1398039    .1807964
      Dental consultation  |   .3235917   .1438569     2.25   0.025     .0405601    .6066232
                           |
                 fac_pop01 |   -1.12813   .2994017    -3.77   0.000    -1.717189    -.539072
          fac_consultrms01 |   .1366501   .2653941     0.51   0.607    -.3855001    .6588003
              fac_diabetes |   .1797619   .0824758     2.18   0.030     .0174947    .3420291
               fac_nurserm |  -.1329903   .1386105    -0.96   0.338    -.4056998    .1397192
             fac_nutrition |   -.064361   .0632269    -1.02   0.309    -.1887569     .060035
                     _cons |   1.398286     .13636    10.25   0.000     1.130005    1.666568
---------------------------+--------------------------------------------------------------------
unidmed                    |
                 var(_cons)|    .181121   .0300568                                 .1306692    .2510523
------------------------------------------------------------------------------------------------
*IV) Add RE for States for Communication
Note: Stage 1 is sampled with replacement; further stages will be ignored for variance
      estimation.

      pweight: <none>
          VCE: linearized
  Single unit: missing
     Strata 1: <one>
         SU 1: entidad
        FPC 1: <zero>
     Weight 1: state_weight
     Strata 2: <one>
         SU 2: unidmed
        FPC 2: <zero>
     Weight 2: facility_weight
```

For peer review only

```
     Strata 3: <one>
         SU 3: <observations>
        FPC 3: <zero>
     Weight 3: rescaled_patient_weights
(running melogit on estimation sample)

Survey: Mixed-effects logistic regression

Number of strata   =         1                 Number of obs     =     27,337
Number of PSUs     =        32                 Population size   = 68,852.018
                                               Subpop. no. obs   =     25,745
                                               Subpop. size      = 64,704.336
                                               Design df         =         31
                                               F(  22,     10)   =      13.90
                                               Prob > F          =     0.0001
```

Communication	Coef.	Linearized Std. Err.	t	P>|t|	[95% Conf.	Interval]
**age_quart**						
25-50 Quartile	.0738536	.0644009	1.15	0.260	-.0574928	.2052
50-75 Quartile	.1445787	.0650096	2.22	0.034	.0119908	.2771666
Top Quartile	.2612484	.0713457	3.66	0.001	.1157378	.406759
sex_male	.1662661	.0300724	5.53	0.000	.1049331	.2275991
**education**						
Completed Primary School	-.1035327	.0603783	-1.71	0.096	-.2266751	.0196097
High School or higher	-.1703817	.0528894	-3.22	0.003	-.2782504	-.0625131
**icd10_bin**						
Endocrine, nutritional ..	.0206051	.072258	0.29	0.777	-.1267661	.1679763
Diseases of the respira..	-.0619315	.0669224	-0.93	0.362	-.1984205	.0745576
Diseases of the digesti..	-.1292431	.0904469	-1.43	0.163	-.3137108	.0552246
Diseases of the musculo..	-.1966096	.107644	-1.83	0.077	-.416151	.0229318
Pregnancy, childbirth a..	.2657592	.1218505	2.18	0.037	.0172436	.5142748
Symptoms, signs and abn..	-.1181645	.1080201	-1.09	0.282	-.3384729	.1021439
Injury, poisoning and c..	-.1324661	.0924996	-1.43	0.162	-.3211202	.0561881
Factors influencing hea..	.1104231	.1053465	1.05	0.303	-.1044326	.3252788
Other	-.1211925	.0796557	-1.52	0.138	-.2836515	.0412665
**service**						
PREVENIMS (preventive ..)	.0194082	.0895295	0.22	0.830	-.1631884	.2020047
Dental consultation	.3171764	.1299256	2.44	0.021	.0521915	.5821614
fac_pop01	-1.173962	.4244646	-2.77	0.009	-2.039664	-.3082611
fac_consultrms01	.5061666	.3509444	1.44	0.159	-.2095892	1.221922
fac_diabetes	.0251968	.0756337	0.33	0.741	-.1290591	.1794527
fac_nurserm	-.1401575	.1134644	-1.24	0.226	-.3715697	.0912548
fac_nutrition	-.000692	.0854003	-0.01	0.994	-.174867	.173483
_cons	1.360566	.1373071	9.91	0.000	1.080526	1.640605
**entidad**						
var(_cons)	.1037982	.0371667			.0500072	.2154505
**entidad>unidmed**						
var(_cons)	.1064024	.0311615			.0585527	.1933555

*V) Add state poverty level for Communication

For peer review only

```
(running melogit on estimation sample)

Survey: Mixed-effects logistic regression

Number of strata   =         1                Number of obs     =     27,337
Number of PSUs     =        32                Population size   = 68,852.018
                                              Subpop. no. obs   =     25,745
                                              Subpop. size      = 64,704.336
                                              Design df         =         31
                                              F(  23,      9)   =      12.02
                                              Prob > F          =     0.0003

------------------------------------------------------------------------------------------
                          |             Linearized
            Communication |      Coef.   Std. Err.      t    P>|t|     [95% Conf. Interval]
--------------------------+---------------------------------------------------------------
                          |
                age_quart |
         25-50 Quartile   |   .0738168   .0643873     1.15   0.260    -.0575019    .2051356
         50-75 Quartile   |    .144545   .0649773     2.22   0.034     .0120228    .2770672
           Top Quartile   |   .2612314   .0713038     3.66   0.001     .1158063    .4066565
                          |
                 sex_male |   .1661988   .0300511     5.53   0.000     .1049092    .2274884
                          |
                education |
Completed Primary School  |  -.1034459   .0603547    -1.71   0.097    -.2265401    .0196483
   High School or higher  |  -.1704332   .0528333    -3.23   0.003    -.2781874   -.0626791
                          |
                icd10_bin |
Endocrine, nutritional .. |   .0205323   .0722378     0.28   0.778    -.1267977    .1678623
Diseases of the respira.. |  -.0619767   .0669156    -0.93   0.362     -.198452    .0744987
Diseases of the digesti.. |  -.1293143   .0904143    -1.43   0.163    -.3137155     .055087
Diseases of the musculo.. |   -.196657   .1076437    -1.83   0.077    -.4161979    .0228839
Pregnancy, childbirth a.. |   .2656754    .121806     2.18   0.037     .0172505    .5141003
Symptoms, signs and abn.. |  -.1183117   .1078812    -1.10   0.281    -.3383369    .1017135
Injury, poisoning and c.. |  -.1324501   .0925091    -1.43   0.162    -.3211235    .0562234
Factors influencing hea.. |   .1102726   .1052054     1.05   0.303    -.1042952    .3248403
                    Other |  -.1212962   .0796063    -1.52   0.138    -.2836544    .0410619
                          |
                  service |
PREVENIMS (preventive ..) |   .0193896   .0895162     0.22   0.830    -.1631799    .2019592
     Dental consultation  |   .3171689   .1299158     2.44   0.021      .052204    .5821339
                          |
                fac_pop01 |  -1.174488   .4249714    -2.76   0.010    -2.041223   -.3077532
         fac_consultrms01 |   .5082048   .3527097     1.44   0.160    -.2111514    1.227561
             fac_diabetes |   .0250445   .0757642     0.33   0.743    -.1294775    .1795666
              fac_nurserm |  -.1392225     .11319    -1.23   0.228     -.370075      .09163
            fac_nutrition |  -.0012475   .0857079    -0.01   0.988    -.1760498    .1735549
          state_poverty01 |   .1140767   .2403637     0.47   0.638    -.3761483    .6043017
                    _cons |   1.310954   .2084665     6.29   0.000     .8857833    1.736124
--------------------------+---------------------------------------------------------------
entidad                   |
                var(_cons)|   .1028885   .0372656                         .0491533    .2153676
--------------------------+---------------------------------------------------------------
entidad>unidmed           |
                var(_cons)|   .1064125   .0311536                         .0585704    .1933336
------------------------------------------------------------------------------------------
*I) Flat Model with Individual Covariates for Share_concerns
Note: Stage 1 is sampled with replacement; further stages will be ignored for variance
      estimation.
```

For peer review only

```
      pweight: <none>
          VCE: linearized
  Single unit: missing
     Strata 1: <one>
         SU 1: unidmed
        FPC 1: <zero>
     Weight 1: facility_weight
     Strata 2: <one>
         SU 2: <observations>
        FPC 2: <zero>
     Weight 2: rescaled_patient_weights
(running logit on estimation sample)

Survey: Logistic regression

Number of strata   =         1                  Number of obs     =     27,337
Number of PSUs     =       319                  Population size   = 68,852.018
                                                Subpop. no. obs   =     25,745
                                                Subpop. size      = 64,704.336
                                                Design df         =        318
                                                F(  17,    302)   =      34.31
                                                Prob > F          =     0.0000

------------------------------------------------------------------------------------------
                          |             Linearized
           Share_concerns |      Coef.   Std. Err.      t    P>|t|     [95% Conf. Interval]
--------------------------+---------------------------------------------------------------
                age_quart |
          25-50 Quartile  |  -.1963475   .0819595    -2.40   0.017    -.3575989   -.0350961
          50-75 Quartile  |   -.216171   .0921544    -2.35   0.020    -.3974804   -.0348616
            Top Quartile  |  -.2414114   .1092795    -2.21   0.028    -.4564137   -.0264092
                          |
                 sex_male |   .0664078   .0667259     1.00   0.320    -.0648722    .1976878
                          |
                education |
 Completed Primary School  |  -.0382979   .0763811    -0.50   0.616     -.188574    .1119781
    High School or higher  |   .0470606   .0890162     0.53   0.597    -.1280745    .2221956
                          |
                icd10_bin |
Endocrine, nutritional ..  |  -.0009121   .1026431    -0.01   0.993    -.2028574    .2010332
Diseases of the respira..  |   .1319851   .1402913     0.94   0.348    -.1440313    .4080016
Diseases of the digesti..  |  -.1700052   .1398109    -1.22   0.225    -.4450764    .1050659
Diseases of the musculo..  |  -.2259857   .1274163    -1.77   0.077    -.4766712    .0246999
Pregnancy, childbirth a..  |  -.0919085   .1409401    -0.65   0.515    -.3692014    .1853844
Symptoms, signs and abn..  |  -.1118509   .1397744    -0.80   0.424    -.3868504    .1631486
Injury, poisoning and c..  |  -.4820752   .1186258    -4.06   0.000    -.7154658   -.2486845
Factors influencing hea..  |  -.9587249   .1114824    -8.60   0.000    -1.178061   -.7393887
                    Other  |  -.1475031   .1128718    -1.31   0.192     -.369573    .0745667
                          |
                  service |
PREVENIMS (preventive ..)  |   -1.27743   .0846704   -15.09   0.000    -1.444015   -1.110845
      Dental consultation  |   .0949897   .1941323     0.49   0.625    -.2869563    .4769358
                          |
                    _cons |     2.8449   .1702974    16.71   0.000     2.509848    3.179951
------------------------------------------------------------------------------------------
*II) Add RE for Facility for Share_concerns
(running melogit on estimation sample)

Survey: Mixed-effects logistic regression

Number of strata   =         1                  Number of obs     =     27,337
```

For peer review only

```
Number of PSUs     =       319                 Population size   = 68,852.018
                                               Subpop. no. obs   =     25,745
                                               Subpop. size      = 64,704.336
                                               Design df         =        318
                                               F(  17,    302)   =      33.45
                                               Prob > F          =     0.0000

------------------------------------------------------------------------------
                           |             Linearized
            Share_concerns |      Coef.   Std. Err.      t    P>|t|     [95% Conf. Interval]
---------------------------+--------------------------------------------------------------
                           |
                 age_quart |
           25-50 Quartile  |  -.1977085   .0832451    -2.38   0.018    -.3614893   -.0339277
           50-75 Quartile  |  -.2030779   .0908244    -2.24   0.026    -.3817707   -.0243852
             Top Quartile  |  -.2280504   .1091908    -2.09   0.038    -.4428781   -.0132227
                           |
                  sex_male |   .0632317   .0665015     0.95   0.342    -.0676069    .1940702
                           |
                 education |
 Completed Primary School  |  -.0133517    .077376    -0.17   0.863    -.1655852    .1388818
      High School or higher |   .0839919    .089364     0.94   0.348    -.0918275    .2598112
                           |
                 icd10_bin |
Endocrine, nutritional ..  |  -.0003744   .1034186    -0.00   0.997    -.2038456    .2030968
Diseases of the respira..  |   .1421993   .1407525     1.01   0.313    -.1347245    .4191231
Diseases of the digesti..  |  -.1560012   .1416805    -1.10   0.272    -.4347508    .1227484
Diseases of the musculo..  |  -.2136876   .1277391    -1.67   0.095    -.4650081     .037633
Pregnancy, childbirth a..  |  -.0831523   .1408744    -0.59   0.555    -.3603158    .1940113
Symptoms, signs and abn..  |  -.1157391   .1400367    -0.83   0.409    -.3912546    .1597765
Injury, poisoning and c..  |  -.4752238   .1189693    -3.99   0.000    -.7092901   -.2411575
Factors influencing hea..  |  -.9576453   .1108565    -8.64   0.000     -1.17575   -.7395405
                    Other  |  -.1429192   .1136525    -1.26   0.209     -.366525    .0806866
                           |
                   service |
PREVENIMS (preventive ..)  |   -1.30918   .0861955   -15.19   0.000    -1.478766   -1.139595
     Dental consultation   |   .0919705   .1951279     0.47   0.638    -.2919343    .4758752
                           |
                     _cons |   2.919982   .1736988    16.81   0.000     2.578238    3.261726
---------------------------+--------------------------------------------------------------
unidmed                    |
                var(_cons)|   .1902232   .0341005                       .133687    .2706686
------------------------------------------------------------------------------
*III) Facility-level Effects for Share_concerns
(running melogit on estimation sample)

Survey: Mixed-effects logistic regression

Number of strata   =         1                 Number of obs     =     27,337
Number of PSUs     =       319                 Population size   = 68,852.018
                                               Subpop. no. obs   =     25,745
                                               Subpop. size      = 64,704.336
                                               Design df         =        318
                                               F(  22,    297)   =      25.49
                                               Prob > F          =     0.0000

------------------------------------------------------------------------------
                           |             Linearized
            Share_concerns |      Coef.   Std. Err.      t    P>|t|     [95% Conf. Interval]
---------------------------+--------------------------------------------------------------
                           |
```

For peer review only

```
                age_quart |
        25-50 Quartile  |  -.1998499   .0837436    -2.39   0.018    -.3646113   -.0350884
        50-75 Quartile  |  -.1962364   .0910245    -2.16   0.032    -.3753228   -.0171501
          Top Quartile  |  -.2198729   .1105111    -1.99   0.047    -.4372981   -.0024477
                        |
               sex_male |   .0557743   .0668492     0.83   0.405    -.0757483     .187297
                        |
              education |
Completed Primary School |  -.0020449   .0774564    -0.03   0.979    -.1544367    .1503469
   High School or higher |   .1026721   .0897995     1.14   0.254    -.0740041    .2793483
                        |
              icd10_bin |
Endocrine, nutritional .. |  -.0002771     .10367    -0.00   0.998    -.2042429    .2036887
Diseases of the respira.. |   .1375723   .1420331     0.97   0.333     -.141871    .4170156
Diseases of the digesti.. |  -.1547567   .1421003    -1.09   0.277    -.4343323    .1248189
Diseases of the musculo.. |  -.2124765   .1278028    -1.66   0.097    -.4639224    .0389694
Pregnancy, childbirth a.. |  -.0819705   .1415853    -0.58   0.563    -.3605328    .1965918
Symptoms, signs and abn.. |  -.1239627   .1402149    -0.88   0.377    -.3998288    .1519033
Injury, poisoning and c.. |  -.4691149   .1192677    -3.93   0.000    -.7037683   -.2344615
Factors influencing hea.. |  -.9743061   .1124096    -8.67   0.000    -1.195467   -.7531456
                    Other |  -.1458597   .1144054    -1.27   0.203    -.3709467    .0792273
                        |
                service |
PREVENIMS (preventive ..) |  -1.313699   .0862879   -15.22   0.000    -1.483467   -1.143932
      Dental consultation |   .0965886    .195796     0.49   0.622    -.2886306    .4818079
                        |
             fac_pop01 |  -.9535991   .3991065    -2.39   0.017    -1.738822   -.1683763
       fac_consultrms01 |  -.1106742   .3467813    -0.32   0.750    -.7929497    .5716013
           fac_diabetes |   .1862659   .0946715     1.97   0.050     4.16e-06    .3725275
            fac_nurserm |    .064487   .1392731     0.46   0.644    -.2095262    .3385002
          fac_nutrition |    -.01714     .07498    -0.23   0.819    -.1646596    .1303795
                  _cons |   3.054871    .183727    16.63   0.000     2.693397    3.416345
--------------------------+----------------------------------------------------------------
unidmed                   |
               var(_cons)|   .1563907   .0314717                         .1052597    .2323589
-------------------------------------------------------------------------------------------
*IV) Add RE for States for Share_concerns
Note: Stage 1 is sampled with replacement; further stages will be ignored for variance
      estimation.

      pweight: <none>
          VCE: linearized
  Single unit: missing
     Strata 1: <one>
         SU 1: entidad
        FPC 1: <zero>
     Weight 1: state_weight
     Strata 2: <one>
         SU 2: unidmed
        FPC 2: <zero>
     Weight 2: facility_weight
     Strata 3: <one>
         SU 3: <observations>
        FPC 3: <zero>
     Weight 3: rescaled_patient_weights
(running melogit on estimation sample)

Survey: Mixed-effects logistic regression

Number of strata   =          1                 Number of obs     =     27,337
Number of PSUs     =         32                 Population size   = 68,852.018
```

For peer review only

```
Subpop. no. obs   =     25,745
Subpop. size      = 64,704.336
Design df         =         31
F(  22,     10)   =      25.24
Prob > F          =     0.0000
```

Share_concerns	Coef.	Linearized Std. Err.	t	P>|t|	[95% Conf.	Interval]
age_quart						
25-50 Quartile	-.1891676	.0672534	-2.81	0.008	-.3263319	-.0520033
50-75 Quartile	-.1827254	.07314	-2.50	0.018	-.3318954	-.0335555
Top Quartile	-.2018592	.0792979	-2.55	0.016	-.3635883	-.0401301
sex_male	.0613961	.0618973	0.99	0.329	-.0648443	.1876365
education						
Completed Primary School	.0091232	.1052271	0.09	0.931	-.2054889	.2237353
High School or higher	.1288565	.1066328	1.21	0.236	-.0886226	.3463356
icd10_bin						
Endocrine, nutritional ..	.0101802	.1002269	0.10	0.920	-.1942339	.2145944
Diseases of the respira..	.1504957	.1590078	0.95	0.351	-.1738028	.4747942
Diseases of the digesti..	-.1543164	.1409729	-1.09	0.282	-.4418325	.1331998
Diseases of the musculo..	-.1992548	.111818	-1.78	0.085	-.4273091	.0287995
Pregnancy, childbirth a..	-.0697851	.1219999	-0.57	0.571	-.3186055	.1790352
Symptoms, signs and abn..	-.1263413	.1371278	-0.92	0.364	-.4060153	.1533326
Injury, poisoning and c..	-.475034	.1025005	-4.63	0.000	-.6840852	-.2659828
Factors influencing hea..	-.9570296	.1220179	-7.84	0.000	-1.205887	-.7081725
Other	-.1401664	.0923261	-1.52	0.139	-.3284668	.048134
service						
PREVENIMS (preventive ..)	-1.324286	.0595103	-22.25	0.000	-1.445658	-1.202914
Dental consultation	.0855222	.199395	0.43	0.671	-.3211465	.4921908
fac_pop01	-.8513804	.3615294	-2.35	0.025	-1.588725	-.1140363
fac_consultrms01	-.0722481	.4565006	-0.16	0.875	-1.003287	.8587909
fac_diabetes	.0805323	.1435956	0.56	0.579	-.212333	.3733975
fac_nurserm	-.0310571	.180736	-0.17	0.865	-.3996707	.3375565
fac_nutrition	.0720496	.1298929	0.55	0.583	-.1928687	.3369679
_cons	3.028693	.2335348	12.97	0.000	2.552395	3.50499
entidad						
var(_cons)	.1183331	.0373483			.062165	.2252507
entidad>unidmed						
var(_cons)	.1041221	.0320072			.055624	.1949051

*V) Add state poverty level for Share_concerns
(running melogit on estimation sample)

Survey: Mixed-effects logistic regression

```
Number of strata   =          1                 Number of obs     =     27,337
Number of PSUs     =         32                 Population size   = 68,852.018
                                                Subpop. no. obs   =     25,745
                                                Subpop. size      = 64,704.336
                                                Design df         =         31
                                                F(  23,      9)   =      21.88
```

For peer review only

```
                                             Prob > F          =     0.0000

------------------------------------------------------------------------------------------
                           |             Linearized
            Share_concerns |      Coef.   Std. Err.      t    P>|t|     [95% Conf. Interval]
---------------------------+--------------------------------------------------------------
                           |
                 age_quart |
            25-50 Quartile |   -.188914   .0671811    -2.81   0.008    -.3259307   -.0518973
            50-75 Quartile |  -.1824825   .0729888    -2.50   0.018    -.3313442   -.0336209
              Top Quartile |  -.2016701   .0791763    -2.55   0.016    -.3631513    -.040189
                           |
                  sex_male |   .0615178   .0618745     0.99   0.328     -.064676    .1877116
                           |
                 education |
  Completed Primary School |   .0087758     .10525     0.08   0.934     -.205883    .2234346
     High School or higher |   .1289782   .1066099     1.21   0.235     -.088454    .3464105
                           |
                 icd10_bin |
Endocrine, nutritional ..  |   .0102353   .1002019     0.10   0.919    -.1941279    .2145984
Diseases of the respira..  |   .1504319   .1590145     0.95   0.351    -.1738804    .4747442
Diseases of the digesti..  |  -.1541799   .1410199    -1.09   0.283    -.4417918    .1334321
Diseases of the musculo..  |  -.1992044   .1118135    -1.78   0.085    -.4272496    .0288409
Pregnancy, childbirth a..  |  -.0696704   .1218989    -0.57   0.572    -.3182849    .1789441
Symptoms, signs and abn..  |  -.1260352   .1370289    -0.92   0.365    -.4055076    .1534372
Injury, poisoning and c..  |  -.4749992   .1025226    -4.63   0.000    -.6840954   -.2659029
Factors influencing hea..  |  -.9567239   .1218248    -7.85   0.000    -1.205187   -.7082605
                     Other |  -.1399324    .092311    -1.52   0.140     -.328202    .0483371
                           |
                   service |
PREVENIMS (preventive ..)  |  -1.324235   .0594536   -22.27   0.000    -1.445492   -1.202979
      Dental consultation  |    .085601   .1994053     0.43   0.671    -.3210887    .4922907
                           |
                 fac_pop01 |  -.8508376   .3611301    -2.36   0.025    -1.587367   -.1143078
          fac_consultrms01 |  -.0771689   .4577233    -0.17   0.867    -1.010702    .8563639
              fac_diabetes |   .0812226   .1437852     0.56   0.576    -.2120294    .3744745
               fac_nurserm |  -.0326914   .1811533    -0.18   0.858    -.4021559    .3367731
             fac_nutrition |   .0727493   .1299266     0.56   0.580    -.1922378    .3377364
           state_poverty01 |  -.2052001   .2465455    -0.83   0.412    -.7080329    .2976327
                     _cons |   3.117878   .2897188    10.76   0.000     2.526993    3.708764
---------------------------+--------------------------------------------------------------
entidad                    |
                 var(_cons)|   .1154058   .0373783                      .0596138    .2234133
---------------------------+--------------------------------------------------------------
entidad>unidmed            |
                 var(_cons)|   .1042867   .0320328                      .0557393    .1951178
------------------------------------------------------------------------------------------
*I) Flat Model with Individual Covariates for Resolve_doubts
Note: Stage 1 is sampled with replacement; further stages will be ignored for variance
      estimation.

      pweight: <none>
          VCE: linearized
  Single unit: missing
     Strata 1: <one>
         SU 1: unidmed
        FPC 1: <zero>
     Weight 1: facility_weight
     Strata 2: <one>
         SU 2: <observations>
        FPC 2: <zero>
```

For peer review only

```
    Weight 2: rescaled_patient_weights
(running logit on estimation sample)

Survey: Logistic regression

Number of strata   =         1                 Number of obs     =     27,337
Number of PSUs     =       319                 Population size   = 68,852.018
                                               Subpop. no. obs   =     25,745
                                               Subpop. size      = 64,704.336
                                               Design df         =        318
                                               F(  17,    302)   =       5.76
                                               Prob > F          =     0.0000

------------------------------------------------------------------------------
                           |             Linearized
            Resolve_doubts |      Coef.   Std. Err.      t    P>|t|     [95% Conf. Interval]
---------------------------+--------------------------------------------------
                 age_quart |
           25-50 Quartile  |   -.093559   .0632403    -1.48   0.140    -.2179813    .0308633
           50-75 Quartile  |   -.023793   .0725288    -0.33   0.743      -.16649     .118904
             Top Quartile  |   .0377486   .0809958     0.47   0.641    -.1216067    .1971039
                           |
                  sex_male |  -.0073745   .0510991    -0.14   0.885    -.1079096    .0931605
                           |
                 education |
 Completed Primary School  |   .0346189    .065023     0.53   0.595    -.0933106    .1625485
    High School or higher  |   .1394226   .0782083     1.78   0.076    -.0144485    .2932937
                           |
                 icd10_bin |
Endocrine, nutritional ..  |  -.1484384   .0811892    -1.83   0.068    -.3081743    .0112974
Diseases of the respira..  |  -.0113877   .0885284    -0.13   0.898    -.1855631    .1627876
Diseases of the digesti..  |  -.2908084   .1049612    -2.77   0.006    -.4973146   -.0843022
Diseases of the musculo..  |  -.3015726   .1097798    -2.75   0.006     -.517559   -.0855861
Pregnancy, childbirth a..  |  -.1224683   .1018048    -1.20   0.230    -.3227643    .0778277
Symptoms, signs and abn..  |  -.5400882    .093885    -5.75   0.000    -.7248023   -.3553741
Injury, poisoning and c..  |  -.4325446   .1067835    -4.05   0.000    -.6426359   -.2224532
Factors influencing hea..  |   .1288747   .1372838     0.94   0.349    -.1412246    .3989739
                    Other  |  -.3547119   .0771014    -4.60   0.000    -.5064051   -.2030186
                           |
                   service |
PREVENIMS (preventive ..)  |   .1319719   .1027883     1.28   0.200    -.0702592    .3342029
      Dental consultation  |    .141035   .1606949     0.88   0.381    -.1751245    .4571945
                           |
                     _cons |   1.987299   .1189204    16.71   0.000     1.753329    2.221269
------------------------------------------------------------------------------
*II) Add RE for Facility for Resolve_doubts
(running melogit on estimation sample)

Survey: Mixed-effects logistic regression

Number of strata   =         1                 Number of obs     =     27,337
Number of PSUs     =       319                 Population size   = 68,852.018
                                               Subpop. no. obs   =     25,745
                                               Subpop. size      = 64,704.336
                                               Design df         =        318
                                               F(  17,    302)   =       5.57
                                               Prob > F          =     0.0000

------------------------------------------------------------------------------
                           |             Linearized
            Resolve_doubts |      Coef.   Std. Err.      t    P>|t|     [95% Conf. Interval]
```

```
----------------------------+----------------------------------------------------------------
                            |
                  age_quart |
              25-50 Quartile |  -.1048848   .0673424    -1.56   0.120    -.2373777    .0276081
              50-75 Quartile |  -.0497869   .0750062    -0.66   0.507    -.1973579    .0977842
                Top Quartile |   -.011277   .0842838    -0.13   0.894    -.1771014    .1545474
                            |
                   sex_male |  -.0166598   .0516194    -0.32   0.747    -.1182185    .0848989
                            |
                  education |
     Completed Primary School |   .0551933   .0640305     0.86   0.389    -.0707836    .1811701
        High School or higher |   .1606116   .0759891     2.11   0.035     .0111067    .3101165
                            |
                  icd10_bin |
 Endocrine, nutritional ..  |  -.1281452   .0830646    -1.54   0.124    -.2915708    .0352803
 Diseases of the respira..  |  -.0277771   .0915074    -0.30   0.762    -.2078135    .1522593
 Diseases of the digesti..  |   -.279813   .1090472    -2.57   0.011    -.4943581   -.0652679
 Diseases of the musculo..  |  -.2940388   .1185829    -2.48   0.014    -.5273448   -.0607327
 Pregnancy, childbirth a..  |  -.1172595   .1038886    -1.13   0.260    -.3216553    .0871364
 Symptoms, signs and abn..  |   -.540188   .0955076    -5.66   0.000    -.7280947   -.3522813
 Injury, poisoning and c..  |  -.3608253   .1091103    -3.31   0.001    -.5754946    -.146156
 Factors influencing hea..  |   .0528295   .1375516     0.38   0.701    -.2177966    .3234557
                      Other |  -.3751948   .0803828    -4.67   0.000    -.5333442   -.2170455
                            |
                    service |
 PREVENIMS (preventive ..)  |   .2282418   .1137517     2.01   0.046     .0044407    .4520428
       Dental consultation  |    .187277   .1600202     1.17   0.243    -.1275551    .5021091
                            |
                      _cons |   2.121049   .1251103    16.95   0.000       1.8749    2.367197
----------------------------+----------------------------------------------------------------
unidmed                     |
                  var(_cons)|   .2965895   .0481751                        .21546    .4082674
---------------------------------------------------------------------------------------------
*III) Facility-level Effects for Resolve_doubts
(running melogit on estimation sample)

Survey: Mixed-effects logistic regression

Number of strata   =         1                  Number of obs     =     27,337
Number of PSUs     =       319                  Population size   = 68,852.018
                                                Subpop. no. obs   =     25,745
                                                Subpop. size      = 64,704.336
                                                Design df         =        318
                                                F(  22,     297)  =       5.49
                                                Prob > F          =     0.0000

---------------------------------------------------------------------------------------------
                            |             Linearized
             Resolve_doubts |      Coef.   Std. Err.       t    P>|t|     [95% Conf. Interval]
----------------------------+----------------------------------------------------------------
                            |
                  age_quart |
              25-50 Quartile |  -.1056468   .0671555    -1.57   0.117    -.2377722    .0264785
              50-75 Quartile |  -.0469628   .0745427    -0.63   0.529    -.1936219    .0996964
                Top Quartile |   -.007258   .0838846    -0.09   0.931    -.1722969    .1577809
                            |
                   sex_male |   -.020024   .0518374    -0.39   0.700    -.1220117    .0819637
                            |
                  education |
     Completed Primary School |   .0615639   .0639977     0.96   0.337    -.0643486    .1874764
        High School or higher |   .1683225   .0757234     2.22   0.027     .0193404    .3173046
```

```
                          |
               icd10_bin |
Endocrine, nutritional .. |  -.1291846   .0836589    -1.54   0.124    -.2937794    .0354103
Diseases of the respira.. |   -.030711   .0919195    -0.33   0.739    -.2115581    .1501361
Diseases of the digesti.. |  -.2810543   .1088042    -2.58   0.010    -.4951214   -.0669873
Diseases of the musculo.. |  -.2940371   .1177309    -2.50   0.013     -.525667   -.0624072
Pregnancy, childbirth a.. |  -.1172918   .1038053    -1.13   0.259    -.3215239    .0869402
Symptoms, signs and abn.. |  -.5449934   .0955344    -5.70   0.000    -.7329528   -.3570341
Injury, poisoning and c.. |  -.3588143   .1088083    -3.30   0.001    -.5728894   -.1447393
Factors influencing hea.. |   .0482873   .1370837     0.35   0.725    -.2214184    .3179929
                    Other |  -.3767199   .0806631    -4.67   0.000    -.5354206   -.2180191
                          |
                  service |
PREVENIMS (preventive ..) |   .2237131   .1138271     1.97   0.050    -.0002363    .4476626
      Dental consultation |   .1881435   .1598683     1.18   0.240    -.1263897    .5026766
                          |
                fac_pop01 |  -.7013858   .4079466    -1.72   0.087    -1.504001    .1012295
         fac_consultrms01 |  -.2920155   .3627236    -0.81   0.421    -1.005657    .4216258
             fac_diabetes |   .1032776   .0955959     1.08   0.281    -.0848027     .291358
              fac_nurserm |  -.1110546   .1469678    -0.76   0.450    -.4002067    .1780975
            fac_nutrition |   .0866532   .0835638     1.04   0.301    -.0777545     .251061
                    _cons |   2.245155   .1484545    15.12   0.000     1.953078    2.537232
--------------------------+----------------------------------------------------------------
unidmed                   |
                var(_cons)|   .2775229   .0488026                      .1963553    .3922429
-------------------------------------------------------------------------------------------
*IV) Add RE for States for Resolve_doubts
Note: Stage 1 is sampled with replacement; further stages will be ignored for variance
      estimation.

      pweight: <none>
          VCE: linearized
  Single unit: missing
     Strata 1: <one>
         SU 1: entidad
        FPC 1: <zero>
     Weight 1: state_weight
     Strata 2: <one>
         SU 2: unidmed
        FPC 2: <zero>
     Weight 2: facility_weight
     Strata 3: <one>
         SU 3: <observations>
        FPC 3: <zero>
     Weight 3: rescaled_patient_weights
(running melogit on estimation sample)

Survey: Mixed-effects logistic regression

Number of strata   =         1                Number of obs     =     27,337
Number of PSUs     =        32                Population size   = 68,852.018
                                              Subpop. no. obs   =     25,745
                                              Subpop. size      = 64,704.336
                                              Design df         =         31
                                              F(  22,     10)   =      11.27
                                              Prob > F          =     0.0002

-------------------------------------------------------------------------------------------
                          |             Linearized
            Resolve_doubts |      Coef.   Std. Err.      t    P>|t|     [95% Conf. Interval]
--------------------------+----------------------------------------------------------------
```

For peer review only

```
                             |
                   age_quart |
             25-50 Quartile  |  -.1099239   .0645072    -1.70   0.098    -.2414872    .0216394
             50-75 Quartile  |  -.0529968   .0758886    -0.70   0.490    -.2077725     .101779
               Top Quartile  |  -.0151998   .0726482    -0.21   0.836    -.1633668    .1329671
                             |
                    sex_male |  -.0160906   .0625494    -0.26   0.799     -.143661    .1114798
                             |
                   education |
    Completed Primary School |   .0648781   .0520728     1.25   0.222     -.041325    .1710813
       High School or higher |   .1682938   .0598304     2.81   0.008     .0462688    .2903187
                             |
                   icd10_bin |
 Endocrine, nutritional ..   |  -.1281272   .0935731    -1.37   0.181    -.3189709    .0627165
 Diseases of the respira..   |   -.038028   .1186624    -0.32   0.751    -.2800415    .2039855
 Diseases of the digesti..   |  -.2862091   .1537024    -1.86   0.072    -.5996873     .027269
 Diseases of the musculo..   |  -.2985614   .1326061    -2.25   0.032    -.5690134   -.0281094
 Pregnancy, childbirth a..   |  -.1198699   .1062169    -1.13   0.268    -.3365006    .0967608
 Symptoms, signs and abn..   |  -.5383298   .1008745    -5.34   0.000    -.7440647   -.3325948
 Injury, poisoning and c..   |  -.3501022   .1463964    -2.39   0.023    -.6486796   -.0515248
 Factors influencing hea..   |   .0319278   .1117272     0.29   0.777    -.1959413    .2597969
                     Other   |  -.3741038   .0917102    -4.08   0.000    -.5611479   -.1870597
                             |
                     service |
 PREVENIMS (preventive ..)   |   .2284354   .0882579     2.59   0.015     .0484322    .4084386
       Dental consultation   |      .2005   .1554239     1.29   0.207     -.116489    .5174891
                             |
                   fac_pop01 |  -.7551055   .3276321    -2.30   0.028    -1.423316   -.0868956
            fac_consultrms01 |   -.167094   .3567697    -0.47   0.643    -.8947307    .5605427
                fac_diabetes |    .111667   .0980932     1.14   0.264    -.0883954    .3117294
                 fac_nurserm |  -.0246641   .1530908    -0.16   0.873    -.3368948    .2875665
               fac_nutrition |  -.0504709   .1351853    -0.37   0.711    -.3261831    .2252413
                       _cons |   2.322359   .1657151    14.01   0.000     1.984381    2.660337
-----------------------------+----------------------------------------------------------------
entidad                      |
                   var(_cons)|   .1220106   .0330881                      .0701764     .212131
-----------------------------+----------------------------------------------------------------
entidad>unidmed              |
                   var(_cons)|   .1805176   .0319137                      .1258716    .2588877
----------------------------------------------------------------------------------------------
*V) Add state poverty level for Resolve_doubts
(running melogit on estimation sample)

Survey: Mixed-effects logistic regression

Number of strata   =         1                Number of obs     =     27,337
Number of PSUs     =        32                Population size   = 68,852.018
                                              Subpop. no. obs   =     25,745
                                              Subpop. size      = 64,704.336
                                              Design df         =         31
                                              F(  23,      9)   =       9.80
                                              Prob > F          =     0.0006

----------------------------------------------------------------------------------------------
                             |             Linearized
              Resolve_doubts |      Coef.   Std. Err.      t    P>|t|     [95% Conf. Interval]
-----------------------------+----------------------------------------------------------------
                             |
                   age_quart |
             25-50 Quartile  |  -.1098881   .0644738    -1.70   0.098    -.2413833    .0216072
             50-75 Quartile  |  -.0529422   .0758694    -0.70   0.490    -.2076789    .1017945
```

For peer review only

```
               Top Quartile  |  -.0151532   .0726566    -0.21   0.836    -.1633372    .1330308
                             |
                    sex_male |  -.0160394   .0625797    -0.26   0.799    -.1436717    .1115928
                             |
                   education |
     Completed Primary School  |   .0648057   .0520615     1.24   0.223    -.0413743    .1709858
        High School or higher  |   .1683599   .0598359     2.81   0.008     .0463237    .2903961
                             |
                   icd10_bin |
Endocrine, nutritional ..  |  -.1280744   .0935696    -1.37   0.181    -.3189108     .062762
Diseases of the respira..  |  -.0380201    .118674    -0.32   0.751    -.2800573    .2040172
Diseases of the digesti..  |  -.2861059   .1536502    -1.86   0.072    -.5994775    .0272658
Diseases of the musculo..  |   -.298553   .1326011    -2.25   0.032    -.5689948   -.0281112
Pregnancy, childbirth a..  |  -.1198024   .1062616    -1.13   0.268    -.3365243    .0969196
Symptoms, signs and abn..  |  -.5381943   .1008591    -5.34   0.000    -.7438977   -.3324908
Injury, poisoning and c..  |  -.3501081    .146396    -2.39   0.023    -.6486848   -.0515315
Factors influencing hea..  |   .0320651   .1118723     0.29   0.776       -.1961    .2602303
                    Other  |  -.3740188   .0916746    -4.08   0.000    -.5609904   -.1870473
                             |
                     service |
  PREVENIMS (preventive ..)  |   .2284348   .0882488     2.59   0.015     .0484502    .4084194
         Dental consultation  |   .2004468   .1553923     1.29   0.207    -.1164778    .5173714
                             |
                  fac_pop01 |  -.7545314    .327681    -2.30   0.028    -1.422841   -.0862215
           fac_consultrms01 |  -.1695328   .3576691    -0.47   0.639    -.8990036     .559938
               fac_diabetes |   .1119431   .0978927     1.14   0.262    -.0877105    .3115966
                fac_nurserm |  -.0254535    .154103    -0.17   0.870    -.3397486    .2888417
              fac_nutrition |  -.0499346   .1353615    -0.37   0.715    -.3260062    .2261369
            state_poverty01 |  -.0947671   .3001921    -0.32   0.754     -.707013    .5174787
                      _cons |   2.363503   .2054285    11.51   0.000     1.944529    2.782478
----------------------------+----------------------------------------------------------------
entidad                     |
                  var(_cons)|   .1215241    .032831                      .0700435    .2108419
----------------------------+----------------------------------------------------------------
entidad>unidmed             |
                  var(_cons)|   .1805272    .031905                      .1258931     .258871
---------------------------------------------------------------------------------------------
*I) Flat Model with Individual Covariates for Global_rating
Note: Stage 1 is sampled with replacement; further stages will be ignored for variance
      estimation.

      pweight: <none>
          VCE: linearized
  Single unit: missing
     Strata 1: <one>
         SU 1: unidmed
        FPC 1: <zero>
     Weight 1: facility_weight
     Strata 2: <one>
         SU 2: <observations>
        FPC 2: <zero>
     Weight 2: rescaled_patient_weights
(running logit on estimation sample)

Survey: Logistic regression

Number of strata   =         1                Number of obs     =     27,337
Number of PSUs     =       319                Population size   = 68,852.018
                                              Subpop. no. obs   =     25,745
                                              Subpop. size      = 64,704.336
                                              Design df         =        318
```

For peer review only

```
                                                F(  17,    302)    =       2.89
                                                Prob > F           =     0.0001

------------------------------------------------------------------------------------------
                          |             Linearized
            Global_rating |      Coef.   Std. Err.      t    P>|t|     [95% Conf. Interval]
--------------------------+---------------------------------------------------------------
                age_quart |
          25-50 Quartile  |   .0489217    .0536005     0.91   0.362    -.0565347    .1543781
          50-75 Quartile  |    .217218    .0572894     3.79   0.000     .1045039    .3299322
            Top Quartile  |   .2229387    .0676761     3.29   0.001     .0897893    .3560882
                          |
                 sex_male |    .065549     .037209     1.76   0.079     -.007658    .1387559
                          |
                education |
 Completed Primary School |   .1505519    .0574759     2.62   0.009     .0374707     .263633
    High School or higher |   .3580737    .0761552     4.70   0.000     .2082421    .5079053
                          |
                icd10_bin |
Endocrine, nutritional .. |  -.0229643    .0592606    -0.39   0.699    -.1395567    .0936281
Diseases of the respira.. |  -.0762341    .0823095    -0.93   0.355     -.238174    .0857059
Diseases of the digesti.. |  -.1447848    .0953839    -1.52   0.130    -.3324481    .0428785
Diseases of the musculo.. |   .0346319    .0772459     0.45   0.654    -.1173457    .1866095
Pregnancy, childbirth a.. |   .0801048     .077394     1.04   0.301    -.0721642    .2323737
Symptoms, signs and abn.. |   .0791826    .0831464     0.95   0.342    -.0844039    .2427691
Injury, poisoning and c.. |  -.1290744    .0985339    -1.31   0.191    -.3229352    .0647863
Factors influencing hea.. |   .1299533    .0942587     1.38   0.169     -.055496    .3154027
                    Other |  -.0355084    .0696345    -0.51   0.610    -.1725108    .1014941
                          |
                  service |
PREVENIMS (preventive ..) |   .0367234    .0786779     0.47   0.641    -.1180715    .1915183
     Dental consultation  |   .2130081    .1194187     1.78   0.075    -.0219424    .4479586
                          |
                    _cons |  -1.355186    .1089577   -12.44   0.000    -1.569555   -1.140817
------------------------------------------------------------------------------------------
*II) Add RE for Facility for Global_rating
(running melogit on estimation sample)

Survey: Mixed-effects logistic regression

Number of strata   =         1                Number of obs      =     27,337
Number of PSUs     =       319                Population size    = 68,852.018
                                              Subpop. no. obs    =     25,745
                                              Subpop. size       = 64,704.336
                                              Design df          =        318
                                              F(  17,    302)    =       3.55
                                              Prob > F           =     0.0000

------------------------------------------------------------------------------------------
                          |             Linearized
            Global_rating |      Coef.   Std. Err.      t    P>|t|     [95% Conf. Interval]
--------------------------+---------------------------------------------------------------
                          |
                age_quart |
          25-50 Quartile  |   .0347388    .0569718     0.61   0.542    -.0773505    .1468281
          50-75 Quartile  |   .2072743    .0556189     3.73   0.000     .0978469    .3167018
            Top Quartile  |   .1954014    .0614554     3.18   0.002     .0744908    .3163119
                          |
                 sex_male |   .0672075     .038077     1.77   0.079    -.0077073    .1421222
                          |
                education |
```

For peer review only

	Coef.	Std. Err.	t	P>|t|	[95% Conf.	Interval]
Completed Primary School	.1351317	.0523625	2.58	0.010	.0321109	.2381525
High School or higher	.3097884	.0643747	4.81	0.000	.1831343	.4364425
**icd10_bin**						
Endocrine, nutritional ..	-.0404282	.0574374	-0.70	0.482	-.1534335	.0725772
Diseases of the respira..	-.0537951	.0838579	-0.64	0.522	-.2187814	.1111912
Diseases of the digesti..	-.1474168	.1003761	-1.47	0.143	-.344902	.0500683
Diseases of the musculo..	.0180007	.0781801	0.23	0.818	-.1358149	.1718162
Pregnancy, childbirth a..	.0850063	.0768226	1.11	0.269	-.0661384	.236151
Symptoms, signs and abn..	.0493855	.0848675	0.58	0.561	-.1175871	.2163582
Injury, poisoning and c..	-.1015919	.0985322	-1.03	0.303	-.2954493	.0922655
Factors influencing hea..	.1238484	.0922288	1.34	0.180	-.0576073	.3053041
Other	-.0453307	.0695495	-0.65	0.515	-.182166	.0915046
**service**						
PREVENIMS (preventive ..)	.0762168	.0761724	1.00	0.318	-.0736487	.2260823
Dental consultation	.2069281	.123677	1.67	0.095	-.0364005	.4502567
_cons	-1.388976	.1060743	-13.09	0.000	-1.597672	-1.18028
**unidmed**						
var(_cons)	.3686354	.0715938			.2515654	.5401857

*III) Facility-level Effects for Global_rating
(running melogit on estimation sample)

Survey: Mixed-effects logistic regression

Number of strata	=	1	Number of obs	=	27,337
Number of PSUs	=	319	Population size	=	68,852.018
			Subpop. no. obs	=	25,745
			Subpop. size	=	64,704.336
			Design df	=	318
			F(22, 297)	=	3.23
			Prob > F	=	0.0000

Global_rating	Coef.	Linearized Std. Err.	t	P>|t|	[95% Conf.	Interval]
**age_quart**						
25-50 Quartile	.0346015	.0569551	0.61	0.544	-.0774549	.1466578
50-75 Quartile	.2058203	.0556791	3.70	0.000	.0962744	.3153662
Top Quartile	.1928738	.0613832	3.14	0.002	.0721052	.3136424
sex_male	.0683643	.0380877	1.79	0.074	-.0065715	.1433
**education**						
Completed Primary School	.1324671	.0525431	2.52	0.012	.0290911	.2358432
High School or higher	.3057841	.0646895	4.73	0.000	.1785107	.4330575
**icd10_bin**						
Endocrine, nutritional ..	-.04062	.0574806	-0.71	0.480	-.1537103	.0724702
Diseases of the respira..	-.0532555	.0839624	-0.63	0.526	-.2184474	.1119364
Diseases of the digesti..	-.147092	.1004723	-1.46	0.144	-.3447665	.0505824
Diseases of the musculo..	.0169517	.0782781	0.22	0.829	-.1370566	.17096
Pregnancy, childbirth a..	.0855168	.0766488	1.12	0.265	-.065286	.2363196
Symptoms, signs and abn..	.0506526	.0848812	0.60	0.551	-.116347	.2176523
Injury, poisoning and c..	-.1017582	.0985484	-1.03	0.303	-.2956475	.0921311
Factors influencing hea..	.124955	.0923563	1.35	0.177	-.0567516	.3066615

For peer review only

```
                       Other |  -.0451579   .0695521    -0.65   0.517    -.1819983    .0916825
                             |
                     service |
   PREVENIMS (preventive ..) |   .0772609   .0762243     1.01   0.312    -.0727067    .2272285
         Dental consultation |   .2051489   .1237612     1.66   0.098    -.0383453    .4486431
                             |
                  fac_pop01 |  -.2575554    .413633    -0.62   0.534    -1.071358    .5562477
          fac_consultrms01 |   .3334109   .3959264     0.84   0.400    -.4455553    1.112377
               fac_diabetes |    .146368   .0892551     1.64   0.102    -.0292371     .321973
                fac_nurserm |   .0078173     .13793     0.06   0.955    -.2635534    .2791879
              fac_nutrition |   .0520806   .0898294     0.58   0.562    -.1246544    .2288157
                      _cons |  -1.455119   .1263892   -11.51   0.000    -1.703784   -1.206454
-----------------------------+----------------------------------------------------------------
unidmed                      |
                  var(_cons)|   .3628353   .0705424                      .2475066    .5319027
----------------------------------------------------------------------------------------------
*IV) Add RE for States for Global_rating
Note: Stage 1 is sampled with replacement; further stages will be ignored for variance
      estimation.

      pweight: <none>
          VCE: linearized
  Single unit: missing
     Strata 1: <one>
         SU 1: entidad
        FPC 1: <zero>
     Weight 1: state_weight
     Strata 2: <one>
         SU 2: unidmed
        FPC 2: <zero>
     Weight 2: facility_weight
     Strata 3: <one>
         SU 3: <observations>
        FPC 3: <zero>
     Weight 3: rescaled_patient_weights
(running melogit on estimation sample)

Survey: Mixed-effects logistic regression

Number of strata   =         1                  Number of obs     =     27,337
Number of PSUs     =        32                  Population size   = 68,852.018
                                                Subpop. no. obs   =     25,745
                                                Subpop. size      = 64,704.336
                                                Design df         =         31
                                                F(  22,     10)   =       6.24
                                                Prob > F          =     0.0024

----------------------------------------------------------------------------------------------
                             |             Linearized
               Global_rating |      Coef.   Std. Err.      t    P>|t|     [95% Conf. Interval]
-----------------------------+----------------------------------------------------------------
                             |
                   age_quart |
             25-50 Quartile |   .0363162   .0554188     0.66   0.517    -.0767111    .1493436
             50-75 Quartile |   .2075691   .0546569     3.80   0.001     .0960957    .3190425
               Top Quartile |    .199244   .0628679     3.17   0.003     .0710241    .3274639
                             |
                    sex_male |   .0678541   .0436117     1.56   0.130    -.0210925    .1568007
                             |
                   education |
    Completed Primary School |   .1239821   .0543775     2.28   0.030     .0130785    .2348858
```

For peer review only

```
     High School or higher  |   .2998752   .0745267     4.02   0.000      .147877    .4518734
                            |
                 icd10_bin  |
  Endocrine, nutritional .. |  -.0462069   .0662564    -0.70   0.491    -.1813378    .0889239
  Diseases of the respira.. |  -.0563516   .0685583    -0.82   0.417    -.1961773     .083474
  Diseases of the digesti.. |  -.1489402   .0897193    -1.66   0.107    -.3319239    .0340435
  Diseases of the musculo.. |   .0131391   .0812024     0.16   0.873    -.1524743    .1787526
  Pregnancy, childbirth a.. |   .0798801   .1043382     0.77   0.450     -.132919    .2926793
  Symptoms, signs and abn.. |   .0395034   .0893947     0.44   0.662    -.1428183     .221825
  Injury, poisoning and c.. |  -.1059502   .0933654    -1.13   0.265    -.2963702    .0844697
  Factors influencing hea.. |   .1290214   .0980688     1.32   0.198    -.0709913    .3290341
                     Other  |  -.0471156   .0784928    -0.60   0.553    -.2072028    .1129716
                            |
                   service  |
  PREVENIMS (preventive ..) |    .070673    .094898     0.74   0.462    -.1228729    .2642188
       Dental consultation  |   .1922784   .1431519     1.34   0.189    -.0996818    .4842387
                            |
                 fac_pop01  |   .1758157   .4477808     0.39   0.697    -.7374392    1.089071
         fac_consultrms01   |   .0418276   .3516141     0.12   0.906     -.675294    .7589493
              fac_diabetes  |  -.0100024   .0922972    -0.11   0.914    -.1982437     .178239
               fac_nurserm  |   -.083592   .0782994    -1.07   0.294    -.2432847    .0761008
             fac_nutrition  |   .0955035   .1032436     0.93   0.362    -.1150632    .3060701
                     _cons  |  -1.370369   .1658596    -8.26   0.000    -1.708642   -1.032096
----------------------------+----------------------------------------------------------------
entidad                     |
                var(_cons)  |   .2392245   .1050282                      .0977079    .5857086
----------------------------+----------------------------------------------------------------
entidad>unidmed             |
                var(_cons)  |   .2017519   .0374704                      .1381376    .2946617
---------------------------------------------------------------------------------------------
*V) Add state poverty level for Global_rating
(running melogit on estimation sample)

Survey: Mixed-effects logistic regression

Number of strata   =         1                  Number of obs     =     27,337
Number of PSUs     =        32                  Population size   = 68,852.018
                                                Subpop. no. obs   =     25,745
                                                Subpop. size      = 64,704.336
                                                Design df         =         31
                                                F(  23,      9)   =       5.88
                                                Prob > F          =     0.0046

---------------------------------------------------------------------------------------------
                            |             Linearized
             Global_rating  |      Coef.   Std. Err.       t    P>|t|     [95% Conf. Interval]
----------------------------+----------------------------------------------------------------
                            |
                 age_quart  |
           25-50 Quartile   |   .0363813   .0554297     0.66   0.516    -.0766684    .1494309
           50-75 Quartile   |   .2075904   .0546614     3.80   0.001     .0961077    .3190731
             Top Quartile   |    .199295   .0628402     3.17   0.003     .0711316    .3274584
                            |
                  sex_male  |   .0678951   .0436042     1.56   0.130    -.0210363    .1568264
                            |
                 education  |
   Completed Primary School |   .1239033   .0543969     2.28   0.030     .0129602    .2348464
      High School or higher |   .2999678   .0745051     4.03   0.000     .1480136    .4519219
                            |
                 icd10_bin  |
  Endocrine, nutritional .. |  -.0461836    .066243    -0.70   0.491    -.1812871      .08892
```

For peer review only

Diseases of the respira..	-.0563825	.0685725	-0.82	0.417	-.196237	.0834721
Diseases of the digesti..	-.1488815	.0896755	-1.66	0.107	-.331776	.0340129
Diseases of the musculo..	.0131726	.0811984	0.16	0.872	-.1524327	.1787778
Pregnancy, childbirth a..	.0799333	.1043205	0.77	0.449	-.1328296	.2926963
Symptoms, signs and abn..	.0396387	.0893295	0.44	0.660	-.14255	.2218274
Injury, poisoning and c..	-.1060049	.0933765	-1.14	0.265	-.2964475	.0844376
Factors influencing hea..	.1291362	.0980459	1.32	0.197	-.0708297	.3291021
Other	-.0470499	.078471	-0.60	0.553	-.2070927	.1129928
service						
PREVENIMS (preventive ..)	.0706781	.0948876	0.74	0.462	-.1228463	.2642026
Dental consultation	.1923374	.1431533	1.34	0.189	-.0996257	.4843004
fac_pop01	.1758303	.4475757	0.39	0.697	-.7370065	1.088667
fac_consultrms01	.0382857	.3508925	0.11	0.914	-.6773643	.7539357
fac_diabetes	-.0093208	.092181	-0.10	0.920	-.1973252	.1786835
fac_nurserm	-.0847935	.0783051	-1.08	0.287	-.2444979	.0749109
fac_nutrition	.0962518	.1028269	0.94	0.356	-.1134651	.3059686
state_poverty01	-.3154306	.2843108	-1.11	0.276	-.8952863	.264425
_cons	-1.233167	.2230705	-5.53	0.000	-1.688122	-.7782116
entidad						
var(_cons)	.233501	.1086604			.0903863	.6032187
entidad>unidmed						
var(_cons)	.2016778	.0374946			.1380338	.2946665

For peer review only

Holt et al.

STROBE Statement—Checklist of items that should be included in reports of ***cross-sectional studies***

	Item No	Recommendation	Page No
**Title and abstract**	1	(*a*) Indicate the study's design with a commonly used term in the title or the abstract	2
		(*b*) Provide in the abstract an informative and balanced summary of what was done and what was found	2
**Introduction**			
Background/rationale	2	Explain the scientific background and rationale for the investigation being reported	3-4
Objectives	3	State specific objectives, including any prespecified hypotheses	4
**Methods**			
Study design	4	Present key elements of study design early in the paper	4
Setting	5	Describe the setting, locations, and relevant dates, including periods of recruitment, exposure, follow-up, and data collection	4
Participants	6	(*a*) Give the eligibility criteria, and the sources and methods of selection of participants	4
Variables	7	Clearly define all outcomes, exposures, predictors, potential confounders, and effect modifiers. Give diagnostic criteria, if applicable	4-5
Data sources/ measurement	8*	For each variable of interest, give sources of data and details of methods of assessment (measurement). Describe comparability of assessment methods if there is more than one group	4-5
Bias	9	Describe any efforts to address potential sources of bias	5-6
Study size	10	Explain how the study size was arrived at	4
Quantitative variables	11	Explain how quantitative variables were handled in the analyses. If applicable, describe which groupings were chosen and why	5-6
Statistical methods	12	(*a*) Describe all statistical methods, including those used to control for confounding	6
		(*b*) Describe any methods used to examine subgroups and interactions	N/A
		(*c*) Explain how missing data were addressed	6
		(*d*) If applicable, describe analytical methods taking account of sampling strategy	6
		(*e*) Describe any sensitivity analyses	N/A
**Results**			
Participants	13*	(a) Report numbers of individuals at each stage of study—eg numbers potentially eligible, examined for eligibility, confirmed eligible, included in the study, completing follow-up, and analysed	6
		(b) Give reasons for non-participation at each stage	6
		(c) Consider use of a flow diagram	N/A
Descriptive data	14*	(a) Give characteristics of study participants (eg demographic, clinical, social) and information on exposures and potential confounders	6
		(b) Indicate number of participants with missing data for each variable of interest	N/A
Outcome data	15*	Report numbers of outcome events or summary measures	6
Main results	16	(*a*) Give unadjusted estimates and, if applicable, confounder-adjusted estimates and their precision (eg, 95% confidence interval). Make clear which confounders were adjusted for and why they were included	7-8

		(*b*) Report category boundaries when continuous variables were categorized	13
		(*c*) If relevant, consider translating estimates of relative risk into absolute risk for a meaningful time period	N/A
Other analyses	17	Report other analyses done—eg analyses of subgroups and interactions, and sensitivity analyses	N/A
**Discussion**			
Key results	18	Summarise key results with reference to study objectives	8
Limitations	19	Discuss limitations of the study, taking into account sources of potential bias or imprecision. Discuss both direction and magnitude of any potential bias	9
Interpretation	20	Give a cautious overall interpretation of results considering objectives, limitations, multiplicity of analyses, results from similar studies, and other relevant evidence	8-9
Generalisability	21	Discuss the generalisability (external validity) of the study results	10
**Other information**			
Funding	22	Give the source of funding and the role of the funders for the present study and, if applicable, for the original study on which the present article is based	N/A

*Give information separately for exposed and unexposed groups.

Note: An Explanation and Elaboration article discusses each checklist item and gives methodological background and published examples of transparent reporting. The STROBE checklist is best used in conjunction with this article (freely available on the Web sites of PLoS Medicine at http://www.plosmedicine.org/, Annals of Internal Medicine at http://www.annals.org/, and Epidemiology at http://www.epidem.com/). Information on the STROBE Initiative is available at www.strobe-statement.org.

For peer review only - http://bmjopen.bmj.com/site/about/guidelines.xhtml
